# Early axonal degeneration linked to clinical decline in Alzheimer’s disease progression revealed with diffusion MRI

**DOI:** 10.1172/JCI196638

**Published:** 2025-11-27

**Authors:** Zhaoyuan Gong, John P. Laporte, Alexander Y. Guo, Murat Bilgel, Jonghyun Bae, Noam Y. Fox, Angelique de Rouen, Nathan Zhang, Aaliya Taranath, Rafael de Cabo, Josephine M. Egan, Luigi Ferrucci, Mustapha Bouhrara, Alzheimer’s Disease Neuroimaging Initiative

**Affiliations:** 1 National Institute on Aging, National Institutes of Health, Baltimore, Maryland, USA.; 2 The Alzheimer’s Disease Neuroimaging Initiative is detailed in Supplemental Acknowledgments.

**Keywords:** Clinical Research, Neuroscience, Public Health, Biomarkers, Dementia, Neuroimaging

## Abstract

**BACKGROUND:**

Axonal degeneration is believed to be an early hallmark of Alzheimer’s disease (AD). This study investigated the temporal trajectory of axonal loss and its association with cognitive and functional decline using a dMRI-derived axonal density index (ADI).

**METHODS:**

Longitudinal dMRI, CSF, and PET data from the ADNI study were analyzed, including 117 subjects that were cognitively normal (CN) and 88 that were cognitively impaired (CI), consisting of 74 individuals with mild cognitive impairment (MCI) and 14 with AD. Linear mixed-effects models examined group differences and associations between baseline and longitudinal changes in ADI, CSF, or PET biomarkers and clinical outcomes. Results derived from larger CSF (*n* = 527) and PET (tau-PET: *n* = 870; amyloid-PET: *n* = 1,581) datasets are also presented.

**RESULTS:**

Compared with the CN group, the CI group exhibited significantly lower baseline ADI values and steeper longitudinal decline (*P* < 10^–6^). Lower baseline ADI predicted faster cognitive and functional decline in the CI group (MMSE: *P* = 0.03; CDR-SB: *P* < 10^–4^), and longitudinal decreases in ADI were associated with worsening clinical outcomes (MMSE: *P* = 0.001; CDR-SB: *P* < 10^–12^). Compared with CSF and PET biomarkers, ADI demonstrated superior sensitivity in tracking disease progression and matched these biomarkers in predicting future cognitive and functional decline. Furthermore, decreases in ADI were significantly associated with declines in clinical outcomes; this association was observed only with amyloid-PET, but not CSF, biomarkers.

**CONCLUSION:**

Axonal degeneration is an early and clinically meaningful feature of AD. ADI is a promising noninvasive biomarker for early detection, prognosis, and disease monitoring.

**TRIAL REGISTRATION:**

ClinicalTrials.gov NCT00106899.

**FUNDING:**

This work was supported by the National Institute on Aging Intramural Research Program.

## Abstract 2

A new MRI-based biomarker detects early axonal loss linked to cognitive decline, offering a promising tool for predicting dementia risk and monitoring Alzheimer’s disease progression.

## Introduction

Anti-amyloid interventions have failed so far to deliver tangible therapeutic benefits, highlighting the need for a more comprehensive understanding of Alzheimer’s disease (AD) mechanisms (1–3) that extends beyond the traditional amyloid, tau, and neurodegeneration [AT(N)] framework (4, 5). This also emphasizes the importance of identifying reliable biomarkers that can detect AD in its prodromal phase and assessing the risk of progression at an early stage before diagnostic symptoms develop. Accurate and quantitative biomarkers at this early stage are also essential for monitoring disease progression and optimizing therapeutic targets that could potentially slow AD progression or prevent its onset in at-risk individuals. The classical AT(N) biomarker paradigm defines AD based on the development of amyloid-β (Aβ) plaques, neurofibrillary tau tangles, and structural atrophy in brain regions critical for memory and cognition (6). These hallmark pathological features are considered to represent distinct stages of the disease process, with Aβ plaques typically appearing first, followed by tau tangles and eventual structural atrophy. While these biomarkers can be assessed using PET imaging or cerebrospinal fluid (CSF) analysis, these methods are invasive, expensive, or not widely accessible, making them less ideal for routine screening or large-scale population studies. Furthermore, macrostructural changes, such as hippocampal and cortical atrophy, are likely to manifest at more advanced stages of the disease, making them less reliable for early detection and intervention. Importantly, the current AT(N) framework overlooks the complexity of AD as reflected by the involvement of multiple biological pathways beyond amyloid and tau pathogenesis, including neuroinflammation, mitochondrial dysfunction, demyelination, axonal degeneration, synaptic loss, and vascular changes, all of which contribute to the progressive cognitive and functional decline in AD (7, 8). These limitations highlight the need for further expansion of the AT(N) framework, preferentially by the development of noninvasive, sensitive, specific, and cost-effective biomarkers.

Recent evidence suggests that white matter (WM) alterations appear early in AD and other neurodegenerative disorders, contributing to cognitive, motor, and autonomic impairments (9–11). Indeed, brain autopsy studies found that WM degradation is a common feature of AD (11–16), with single-cell transcriptomics studies revealing perturbed WM-related genes (17), reduced major myelin proteins (18), and higher inflammation (19). Reduced WM integrity was observed in a transgenic mouse model of amyloidosis (20), and experimentally induced myelin damage caused accelerated parenchymal amyloidosis, suggesting a role of WM damage in the development of Aβ. For tau pathology, alterations in WM integrity were observed in a transgenic mouse model of fibrillar tau (21, 22), preceding the emergence of tau pathology (21), with clinical investigations suggesting a role of WM hyperintensities in tau pathology in the AD spectrum (23). Additionally, clinical studies in genetic forms of AD, including autosomal dominant AD and Down’s syndrome, highlight impairments in WM integrity decades before the onset of symptoms (9, 24, 25). These collective findings suggest that WM alteration is an important component of AD pathophysiology. WM degeneration disrupts neural pathways, impairing the communication between brain regions whose integrity is essential against cognitive decline and mobility and autonomic dysfunction (26–28). Despite much scientific progress, the temporal sequence of cerebral WM microstructural changes in relation to cognition–function and disease progression remains poorly understood, including in early-onset and sporadic AD. Recent preclinical and clinical studies shed light on the role of myelin in cerebral aging and AD (26–30), but the relevance of axonal integrity deterioration remains unclear. Investigating axonal degeneration in mild cognitive impairment (MCI) is crucial because it represents one of the earliest clinical manifestations along the AD continuum, potentially preceding overt brain atrophy or significant cognitive and functional decline associated with dementia. Understanding these early microstructural changes may reveal mechanisms driving disease progression, identify novel therapeutic targets aimed at preserving WM integrity, and support the development of noninvasive biomarkers to help with early diagnosis and risk stratification (31). Moreover, assessing the degree of axonal degeneration may help in disease phenotyping and in identifying subgroups with specific clinical courses and possibly those that may be more responsive to specific interventions aimed at preserving or restoring WM integrity. This raises a critical question: Are individuals with a clinical diagnosis of cognitive impairment but higher axonal health protected from accelerated cognitive decline and increased dementia risk? Addressing this question requires a reliable and sensitive in vivo measure of axonal integrity.

There is significant interest in leveraging advanced neuroimaging techniques, such as diffusion MRI (dMRI), to noninvasively assess human brain microstructure degeneration, which might precede macrostructural changes and cognitive or functional symptoms by decades (32–34). These methods have the potential to assess WM integrity deterioration with high sensitivity and to provide insight into its role in cognitive decline and neurodegenerative diseases. While various MRI methods can probe cerebral WM microstructural integrity, including relaxation times and diffusion tensor imaging (DTI), only a few methods can specifically quantify axonal density. Axonal density is a quantitative measure that represents the fraction of axonal water relative to the total water content within each brain voxel. It is expected to decline as a result of axonal degeneration and subsequent loss of axonal water. The most clinically adopted techniques are Neurite Orientation Dispersion and Density Imaging (NODDI) and, to a lesser extent, Standard Model Imaging (SMI) (35, 36). Both methods are based on a multicompartment model that distinguishes between water diffusivities within axons and in the extracellular space, providing an estimate of the axonal density index (ADI), a proxy of axonal integrity and health in WM. To address increasing criticisms regarding the physiological reliability of NODDI, we recently introduced a new method, called constrained NODDI (C-NODDI), providing physiologically realistic ADI values in WM that strongly correlate with neurofilament light chain (NfL) concentration level, which itself is a plasma biomarker of axonal degeneration (37), with higher NfL values associated with lower ADI. However, unlike widely adopted but nonspecific DTI, these methods require multishell dMRI data, which are now routinely acquired in clinical investigations, including the Alzheimer’s Disease Neuroimaging Initiative (ADNI).

Using the longitudinal ADNI multishell dMRI, PET, CSF, and cognitive data as well as linear mixed-effects models, we examined the longitudinal changes in ADI as measured using NODDI, C-NODDI, or SMI in patients that were cognitively impaired (CI), including MCI and AD, and those that were cognitively normal (CN). We also evaluated whether baseline ADI level predicts future changes in cognition and function, measured by the Mini-Mental State Examination (MMSE) and Clinical Dementia Rating–Sum of Boxes (CDR-SB) scores, respectively. Furthermore, we investigated the association between longitudinal changes in ADI and longitudinal changes in cognition and function. Finally, we conducted similar analyses using PET and CSF biomarkers of AD pathology and compared results with those obtained by ADI. We hypothesized that (a) C-NODDI provides a powerful noninvasive imaging biomarker to detect axonal degeneration early in the course of AD and to distinguish ADI trajectories between CN and CI, while also predicting cognitive and functional changes with higher or comparable performance as those derived using biomarkers from PET and CSF or other dMRI techniques. (b) Subjects with a clinical diagnosis of mild AD dementia who have higher axonal density exhibit a less longitudinal cognitive and functional decline.

## Results

### Cohort characteristics.

**Figure 1:**
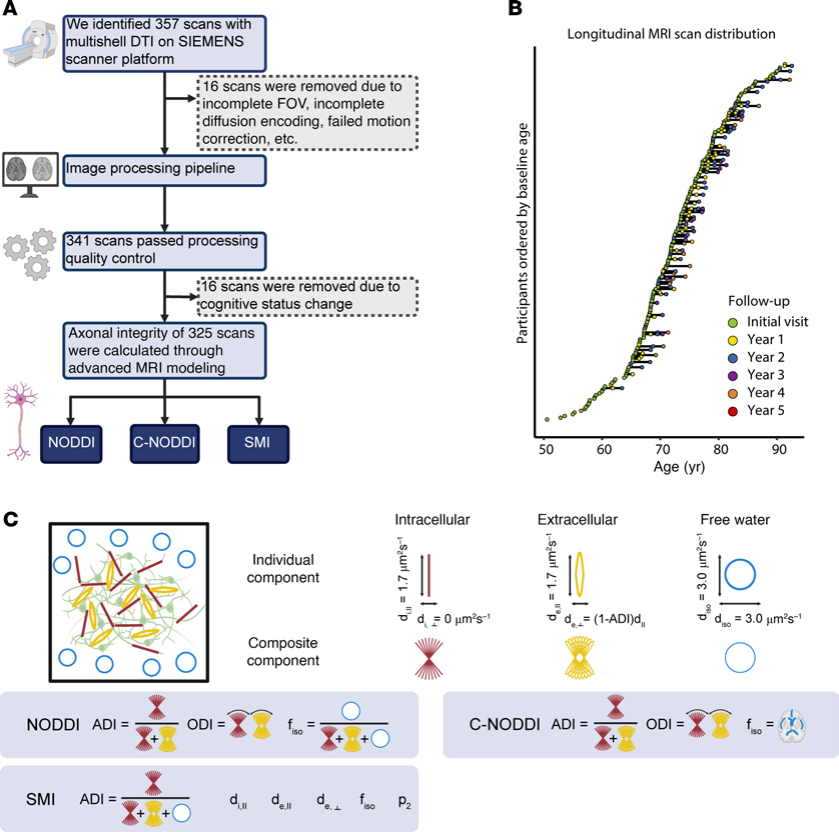
Overview of data acquisition, processing, distribution, and diffusion MRI models. (**A**) The flow chart outlines the data acquisition and processing steps. 357 multishell DTI MRI (dMRI) scans on SIEMENS scanners were downloaded. 16 scans were excluded after failing quality control and preprocessing steps, and another 16 scans were discarded due to changes in the diagnostic group. The final dataset included 325 longitudinal scans from 205 subjects over a maximum span of 4.16 years. (**B**) The longitudinal distribution of dMRI scans is shown, with participants ordered by the age at their first dMRI scan. Time points, from the initial visit to the fifth follow-up, are color-coded. (**C**) Preprocessed images were input into 3 different biophysical models to estimate corresponding ADI maps, a quantitative biomarker of axonal density/integrity. These models are NODDI, C-NODDI, and SMI.

Cohort data availability, longitudinal distribution, and dMRI biophysical models are summarized in Figure 1. Subject characteristics and summary statistics are presented in Table 1. After excluding scans with imaging artifacts or cognitive status changes during longitudinal follow-up, the final cohort included 205 participants and 325 available multishell dMRI measurements (Figure 1A). Among the participants, 117 were CN and 88 were CI, including 74 MCI and 14 AD. A total of 82 participants underwent longitudinal assessment, with 50 participants having 1 follow-up, 25 having 2 follow-ups, and 6 having 3 follow-ups from baseline (Figure 1B). Differences in NODDI, C-NODDI, and SMI models are illustrated in Figure 1C. As expected, the CN group predominantly had a global CDR of 0, while the CI group predominantly had a global CDR of 0.5. The differences between CI and CN at baseline were statistically tested by 2-tailed *t* test for age, χ^2^ test for sex and global CDR, and Wilcoxon’s rank-sum test for WM hyperintensity (WMH) burden and were significantly different (*P* = 0.017 for age, *P* = 0.008 for sex, *P* <10^–46^ for global CDR, and *P* = 0.001 for WMH) (Table 1). At baseline, WMH burden was significantly higher in the CI group (median 0.17% and mean 0.41% of total brain volume) compared with the CN group (median 0.1% and mean 0.22%). Overall, WMH burden in our cohort was low, consistent with expected values for this population. The participants in the CI group are older and more likely to be male compared with participants in the CN group. Additionally, male participants had longer follow-up durations than female participants (Table 1). Each multishell dMRI scan was processed using SMI, NODDI, and C-NODDI techniques to generate corresponding ADI values (ADI_NODDI_, ADI_C-NODDI_, and ADI_SMI_) in whole-brain WM, which served as the region of interest in this study. The details of the image processing pipeline are described in Supplemental Figure 1; supplemental material available online with this article; https://doi.org/10.1172/JCI196638DS1

### Trajectories of ADI over time in CN and CI groups.

**Figure 2:**
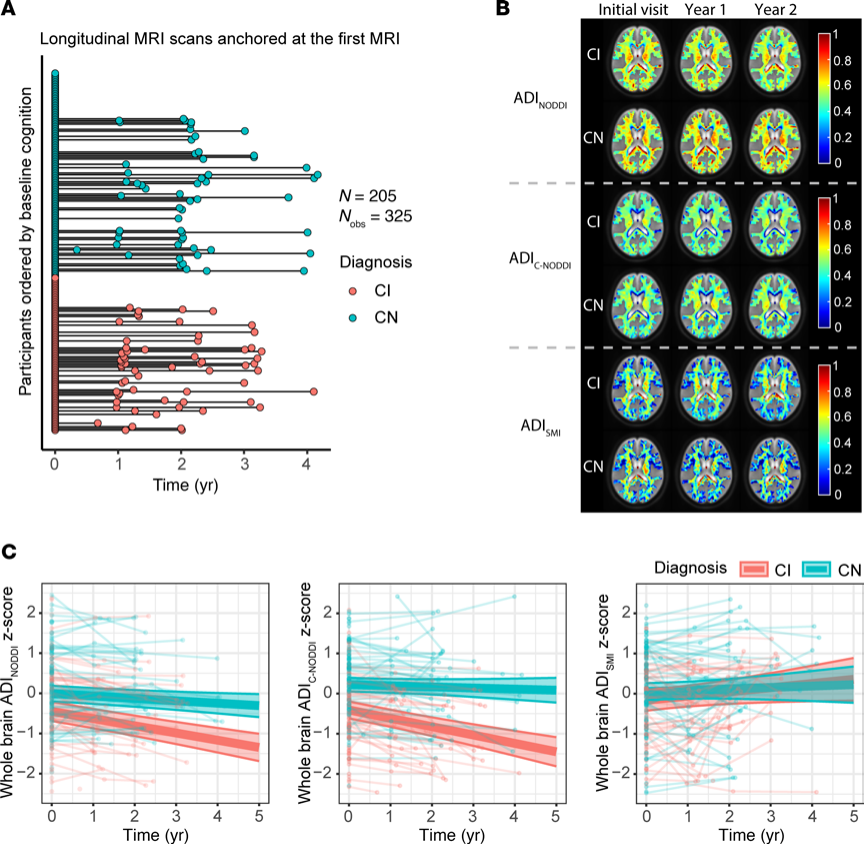
Characterization of longitudinal trajectories of axonal integrity in CN and CI subjects. (**A**) The longitudinal distribution of dMRI scans anchored at the first scan of each subject, with CN and CI groups color-coded. Both the CN and CI groups have similar longitudinal distributions, with many of the participants having over 3 years of follow-up dMRI measurements from baseline. (**B**) Representative ADI maps derived using the NODDI, C-NODDI, or SMI analyses, for 1 CN and 1 CI participant. Images are shown for the middle brain slice at 3 time points. (**C**) Results of the linear mixed-effects model of the association between whole-brain WM ADI and time (in years) given by, ADI_ij_ ~ β_0_ + β_age_ × age_i_ + β_sex_ × sex_i_ + β_time_ × time_ij_ + β_diagnosis_ × diagnosis_i_ + β_time×diagnosis_ × time_ij_ × diagnosis_i_ + b_i_ + ε_ij_. Results are shown for each diagnosis group. CN and CI exhibit significant differences in axonal density/integrity, as measured using ADI_NODDI_ or ADI_C-NODDI_. While the CN group maintained a relatively constant axonal density/integrity over time, the CI group exhibited decreases in ADI_NODDI_ and ADI_C-NODDI_, that is, decreased axonal density/integrity, over time. In contrast, ADI_SMI_ showed a slight increase over time. Full statistical results are shown in Supplemental Table 1.

The longitudinal distribution of dMRI data anchored at their baseline scan is shown in Figure 2A. Representative examples of ADI_NODDI_, ADI_C-NODDI_, and ADI_SMI_ maps from 1 CN subject and 1 CI subject at baseline and at 1- and 2-year follow-ups are displayed in Figure 2B. Derived ADI maps from NODDI and C-NODDI showed lower regional values in the CI subject compared with the CN subject. Moreover, the CN subject exhibited minimal regional variation in ADI over time, while the CI subject demonstrated noticeable decreases in regional values. Interestingly, ADI_SMI_ values in the CI subject were slightly higher than in the CN subject and showed minimal change over time in both groups. As expected, ADI_NODDI_ values were substantially higher than those derived from C-NODDI or SMI, with values exceeding 70% in several cerebral WM structures, which are not physiologically plausible, as discussed previously (37).

Linear mixed-effects models were used to quantitatively analyze longitudinal trajectories of whole-brain WM ADI values as a function of time from baseline. Model equations are provided in the corresponding figure legends. Each model included a Time × Diagnosis term to examine whether ADI trajectories differed between CN and CI groups, adjusting for relevant covariates (Figure 2C). The results revealed that the CI group exhibited significantly lower ADI values compared with the CN group, with a larger effect size and stronger significance observed for C-NODDI (β_diagnosis-CN_ = 0.63, *P* < 10^–6^) compared with NODDI (β_diagnosis-CN_ = 0.44, *P* = 0.001), consistent with lower axonal density/integrity in CI. ADI_SMI_ did not show significant differences between groups (β_diagnosis-CN_ = 0.22, *P* = 0.14). In NODDI and C-NODDI, time since baseline MRI was significantly and negatively associated with ADI values, demonstrating progressive reductions in axonal density/integrity over time. Importantly, the Time × Diagnosis interaction was significant and positively associated with both ADI_NODDI_ and ADI_C-NODDI_, indicating a steeper decline of axonal density in CI compared with CN (Figure 2C). Similarly, the effect size and significance were notably greater for C-NODDI (β_time_ = –0.21, *P* < 10^–8^; β_time×diagnosis-CN_ = 0.18, *P* < 10^–4^) compared with NODDI (β_time_ = –0.18, *P* < 10^–9^; β_time×diagnosis-CN_ = 0.12, *P* = 0.001), highlighting the higher sensitivity of C-NODDI in differentiating ADI trajectories between CN and CI over time (Figure 2C). No significant time × diagnosis interaction was observed for ADI_SMI_ (β_time_ = 0.11, *P* = 0.046; β_time×diagnosis-CN_ = –0.07, *P* = 0.31). Full regression results are provided in Supplemental Table 1.

### Association between baseline ADI and prospective changes in cognition and function.

**Figure 3:**
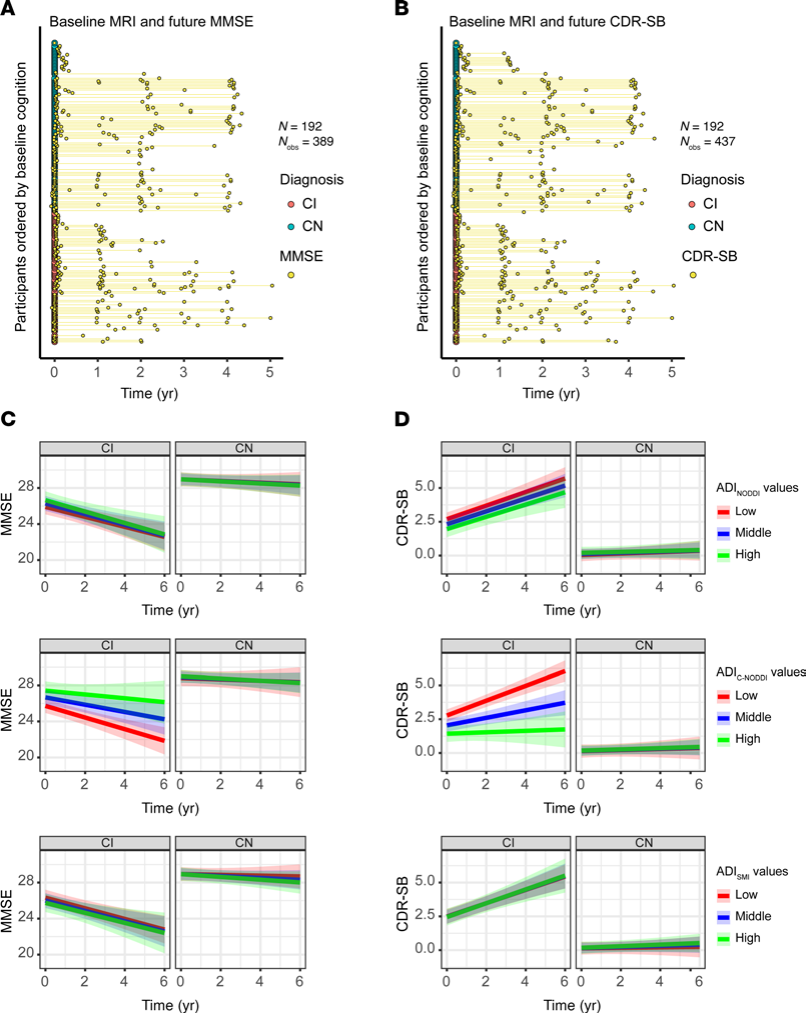
Baseline MRI measurements of axonal density/integrity, as measured using the ADI, predict prospective changes in cognition and function, as measured using the MMSE and CDR-SB scores. Analyses were conducted using the following linear mixed-effects models: MMSE/CDR-SB_ij_ ~ β_0_ + β_age_ × age_i_ + β_sex_ × sex_i_ + β_time_ × time_ij_ + β_diagnosis_ × diagnosis_i_ + β_ADI_ × ADI_i_ + β_time×ADI_ × time_ij_ × ADI_i_ + β_time×diagnosis_ × time_ij_ × diagnosis_i_ + β_ADI×diagnosis_ × ADI_i_ × diagnosis_i_ + β_time×diagnosis×ADI_ × time_ij_ × diagnosis_i_ × ADI_i_ + b_i_ + ε_ij_. Low, middle, and high values are the 25th, 50th, and 75th quantiles of the ADI *z* score. (**A** and **B**) The longitudinal distributions of MMSE and CDR-SB anchored at the first dMRI scan of each subject, with CN and CI subjects color-coded. Both the CN and CI groups have similar longitudinal distributions, with many of the participants having over 4 years of cognitive measurement follow-up from baseline. (**C**) Predicted longitudinal changes in MMSE for CN and CI groups based on ADI_NODDI_, ADI_C-NODDI_, or ADI_SMI_ values. (**D**) Predicted longitudinal changes in CDR-SB for CN and CI groups based on ADI_NODDI_, ADI_C-NODDI_, or ADI_SMI_ values. While the CN group exhibits relatively stable MMSE and CDR-SB, the CI group shows significant decline in MMSE and an increase in CDR-SB scores. Among ADI biomarkers, ADI_C-NODDI_ uniquely predicts differential trajectories within CI subjects, with higher baseline ADI_C-NODDI_ values predicting slower decline in cognition and function (middle row, green prediction lines). These findings highlight the importance of maintaining axonal integrity and the sensitivity of ADI_C-NODDI_ as an imaging biomarker for axonal integrity determination and prediction of cognitive and functional decline. Full results are shown in Supplemental Table 2.

To investigate the association between baseline ADI and prospective changes in cognition and function, linear mixed-effects models were used, incorporating a 3-way interaction term (time × diagnosis × ADI) and adjusting for relevant covariates (Figure 3). Cognitive and functional changes were assessed using the MMSE (Figure 3A) and CDR-SB (Figure 3B). Thirteen participants were excluded from this analysis due to missing cognitive data (6 CN and 7 CI for MMSE; 5 CN and 8 CI for CDR-SB), either because they had no follow-up cognitive assessments or their first cognitive score was collected more than 0.1 years prior to baseline MRI (Figure 3, A and B, and Supplemental Figure 2). The final cohort included 192 participants (111 CN and 81 CI for MMSE; 112 CN and 80 CI for CDR-SB). The average time difference between the first MMSE or CDR-SB measurement and the baseline MRI was 0.067 years (median: 0.023, SD: 0.167, range: –0.082 to 1.61) for MMSE and 0.068 years (median: 0.023, SD: 0.174, range: –0.082 to 1.74) for CDR-SB. The mean prospective follow-up duration referenced to baseline MRI was 1.69 years (median: 1.28, SD: 1.61, range: –0.058 to 5.04) for MMSE and 1.96 years (median: 1.99, SD: 1.60, range: –0.044 to 5.04) for CDR-SB. Analysis revealed that, as expected, the CN group maintained stable MMSE scores over time, while the CI group exhibited a progressive decline (Figure 3C). Notably, this decline was steeper in participants with lower baseline ADI_C-NODDI_ (Figure 3C, red prediction line). This association was statistically significant for C-NODDI (β_time×ADI_ = 0.32, *P* = 0.03) but not for NODDI (β_time×ADI_ = –0.06, *P* = 0.63) nor SMI (β_time×ADI_ = 0.02, *P* = 0.85). Similarly, while CDR-SB scores remained stable in the CN group, the CI group exhibited a prospective increase in CDR-SB scores, indicating increased functional decline over time (Figure 3D). However, the increase was steeper in participants with lower ADI_C-NODDI_ (Figure 3D, red prediction line), whereas participants with higher ADI_C-NODDI_ values (Figure 3D, green prediction line) maintained relatively stable CDR-SB scores despite a clinical diagnosis of cognitive impairment. Compared with NODDI (β_time×ADI_ = –0.04, *P* = 0.6) and SMI (β_time×ADI_ = 0.01, *P* = 0.86), C-NODDI demonstrated superior sensitivity in association with CDR-SB (β_time×ADI_ = –0.37, *P* < 10^–4^), supporting its potential as a prognostic biomarker for predicting dementia progression in individuals with cognitive impairment. Full results of the regression analyses are provided in Supplemental Table 2.

### Association between longitudinal changes in ADI and cognition/function.

**Figure 4:**
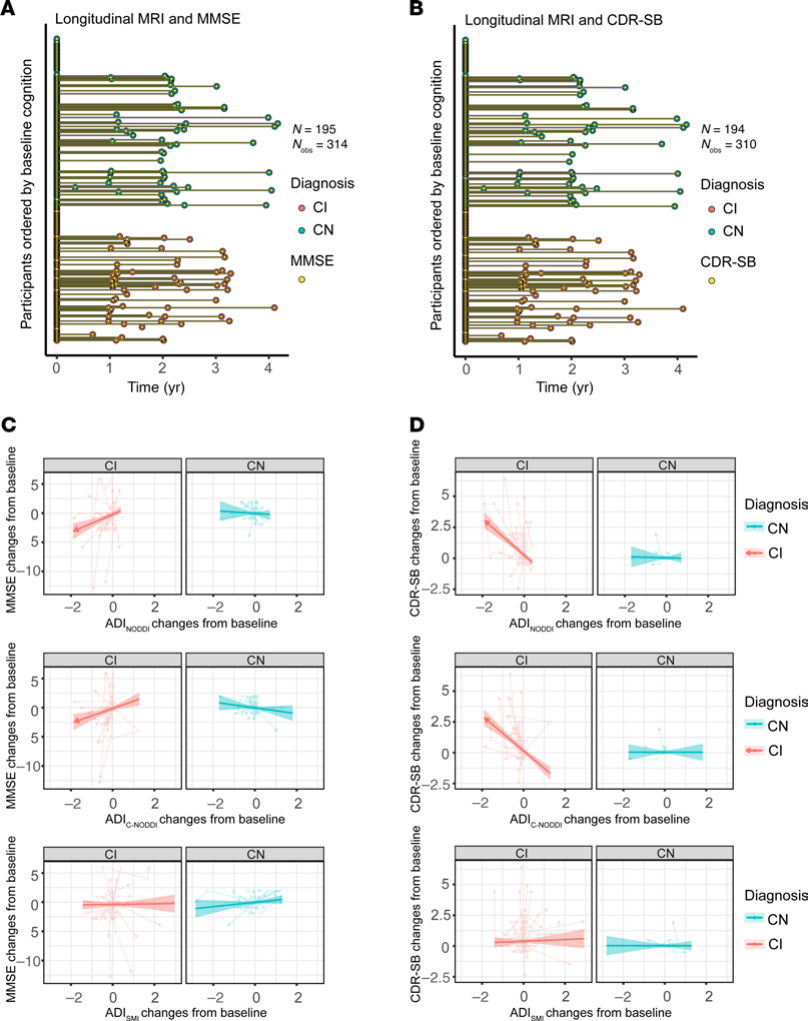
Changes from baseline in axonal integrity, as measured using the ADI, are associated with changes from baseline in cognition and function, as measured using the MMSE and CDR-SB scores. Analyses were conducted using the following linear mixed-effects regression given by, MMSE/CDR-SB changes_ij_ ~ β_0_ + β_sex_ × sex_i_ + β_age_ × age_ij_ + β_diagnosis_ × diagnosis_i_ + β_ADI_ _changes_ × ADI changes_ij_ + β_diagnosis×ADI_ _changes_ × diagnosis_i_ × ADI changes_ij_ + β_baseline_ _ADI_ × baseline ADI_i_ + β_diagnosis×baseline_ _ADI_ × diagnosis_i_ × baseline ADI_i_ +b_i_ + ε_ij_. To isolate the within-subject effect, longitudinal ADI values are split into baseline values and changes from baseline. (**A** and **B**) The longitudinal distribution of MMSE or CDR-SB scores in relation to the longitudinal MRI scans. To enable linear mixed-effects modeling, the MMSE and CDR-SB measurements were aligned to their closest dMRI scans (see Supplemental Figure 3 for original data distribution). (**C** and **D**) Individual trajectories show how changes in ADI from baseline are associated with changes in MMSE or CDR-SB from baseline. Arrow directions indicate forward time progression. Arrows are used to indicate significant directional interpretation within this cohort. For instance, decreases in ADI_NODDI_ and ADI_C-NODDI_ are associated with decreases in MMSE or increases in CDR-SB. The converse — that increases in ADI_NODDI_ and ADI_C-NODDI_ correspond to increases in MMSE or decreases in CDR-SB — is not supported by the raw data; thus, arrows on the fitted lines are directional rather than bidirectional. Decreases in ADI_NODDI_ and ADI_C-NODDI_ are associated with declines in MMSE and increases in CDR-SB for CI subjects, with ADI_C-NODDI_ and ADI_NODDI_ outperforming ADI_SMI_ in detecting such associations. Changes in ADI, that is, in axonal density/integrity, are not associated with changes in MMSE or CDR-SB for CN subjects. These findings underscore the implication of axonal integrity in cognition and further emphasize the sensitivity of ADI_C-NODDI_ as an imaging biomarker for axonal integrity determination. Full results are shown in Supplemental Table 3.

To investigate the association between longitudinal changes in ADI from baseline and changes in cognition and function from baseline, as measured using MMSE (Figure 4A) or CDR-SB (Figure 4B) changes, linear mixed-effects models were used, including an interaction term (diagnosis × ADI changes) and adjusting for relevant covariates. ADI changes were calculated by subtracting the baseline ADI from each subsequent ADI value for each subject. Ten participants (5 CN and 5 CI) were excluded from the MMSE analysis and 11 participants (5 CN and 6 CI) were excluded from the CDR-SB analysis due to missing cognitive data. The final cohort included 195 participants (112 CN and 83 CI) for MMSE, with a mean follow-up interval of 0.955 years (median: 0, SD: 1.263, range: 0–4.167 years), and 194 participants (112 CN and 82 CI) for CDR-SB, with a mean follow-up interval of 0.935 years (median: 0, SD: 1.259, range: 0–4.167 years). While the CN group showed minimal associations between longitudinal changes from baseline in ADI and changes from baseline in MMSE or CDR-SB scores, the CI group demonstrated that declines in ADI from baseline were associated with decreased MMSE scores and increased CDR-SB scores (Figure 4, C and D). This suggests that progressive axonal degeneration is associated with cognitive and functional decline and increased dementia risk among individuals diagnosed with cognitive impairment. This association was statistically significant for ADI_NODDI_ (MMSE: β_ADI_ _changes_ = 1.53, *P* < 10^–4^; β_diagnosis-CN×ADI_ _changes_ = –1.77, *P* = 0.005; CDR-SB: β_ADI_ _changes_ = –1.47, *P* < 10^–14^; β_diagnosis-CN×ADI_ _changes_ = 1.42, *P* < 10^–5^) and ADI_C-NODDI_ (MMSE: β_ADI_ _changes_ = 1.25, *P* = 0.001; β_diagnosis-CN×ADI_ _changes_ = –1.75, *P* = 0.001; CDR-SB: β_ADI_ _changes_ = –1.42; *P* < 10^–12^; β_diagnosis-CN×ADI_ _changes_ = 1.42, *P* < 10^–7^). These results showed that decreases in ADI were strongly linked to cognitive and functional decline, with ADI_NODDI_ and ADI_C-NODDI_ outperforming ADI_SMI_. Full results of the regression analyses are presented in Supplemental Table 3.

### Comparison of ADI with CSF and PET biomarkers of AD pathology performance.

Subsets of participants with available multishell dMRI data and CSF or PET biomarkers of AD pathology were used to compare the performance of ADI_C-NODDI_, CSF-Aβ_42/40_, CSF-tau, CSF-pTau_181_, amyloid-PET, and tau-PET in differentiating CN and CI trajectories and predicting cognitive and functional decline.

**Figure 5:**
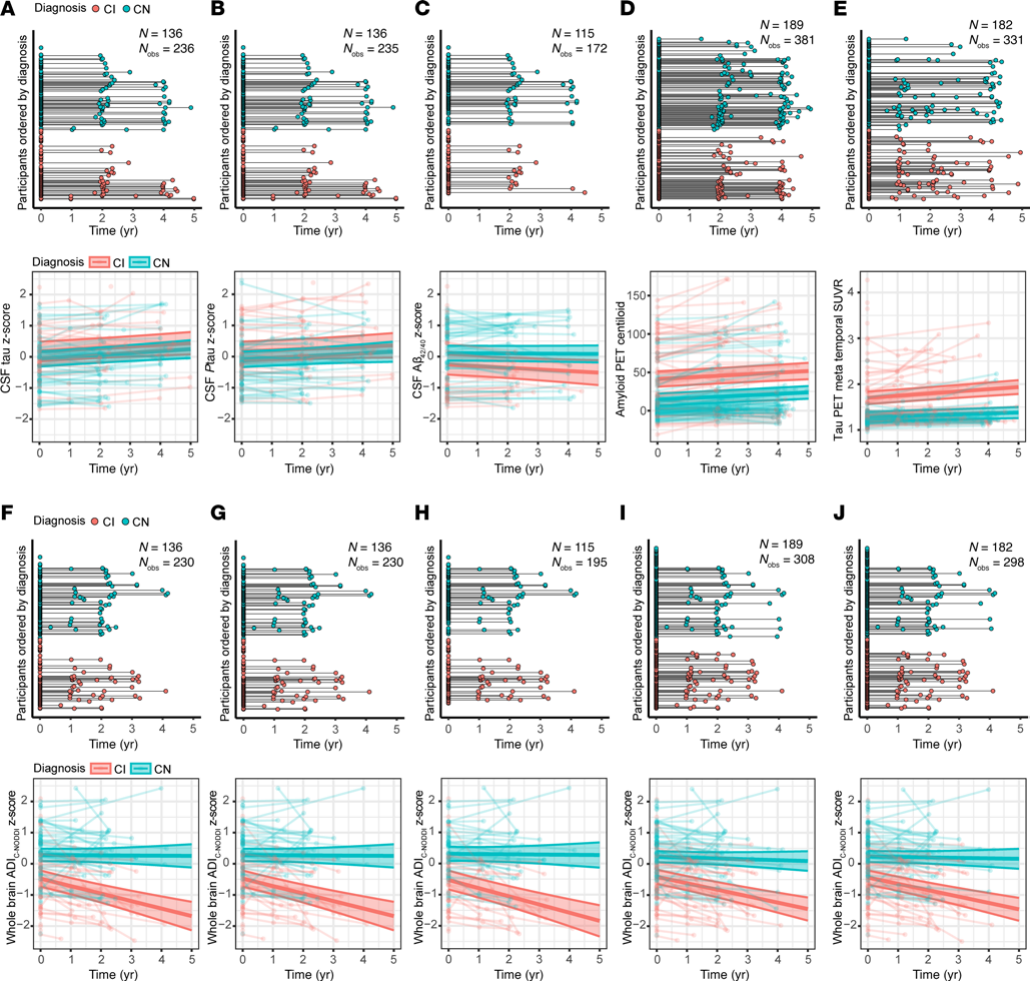
Comparison of CSF and PET biomarkers and ADI_C-NODDI_ in differentiating longitudinal trajectories between the CN and CI groups. Analyses were restricted to participants with both CSF or PET biomarkers and dMRI. The comparison was restricted to ADI_C-NODDI_, as it is the best-performing dMRI biomarker in differentiating axonal degeneration trajectories between CN and CI in the previous analyses. (**A**–**E**) Longitudinal data distributions for the 3 CSF biomarkers of AD pathology, tau, pTau_181_, and Aβ_42/40_, and the 2 PET biomarkers of AD pathology, amyloid-PET and tau-PET. Except for tau-PET, the trajectories of these CSF and PET biomarkers show no significant differentiation over time between the CN and CI groups. (**F**–**J**) Longitudinal dMRI data distribution for the ADI_C-NODDI_ biomarker from the same participants included in the above CSF or PET analyses, for each CSF or PET biomarker. In contrast to CSF and PET biomarkers, the ADI_C-NODDI_ trajectories reveal significant differentiation between diagnosis groups, demonstrating the superior sensitivity of ADI_C-NODDI_ in detecting group differences over time compared with CSF and PET biomarkers of AD pathology. Full results are shown in Supplemental Table 4.

Figure 5A and F, B and G, C and H, D and I, and E and J directly compare the performance of CSF or PET biomarkers with ADI_C-NODDI_ in differentiating CN and CI trajectories using matched subjects and follow-up durations. As shown in Figure 5, the CI group exhibited significantly lower ADI_C-NODDI_ values compared with the CN group, indicating lower axonal density in CI. Furthermore, ADI_C-NODDI_ trajectories revealed a steeper decline in axonal density in CI compared with CN. In contrast, while, as expected, CSF-tau (β_diagnosis-CN_ = –0.24, *P* = 0.19), CSF-pTau_181_ (β_diagnosis-CN_ = –0.25, *P* = 0.16), amyloid-PET (β_diagnosis-CN_ = –0.27, *P* < 10^–5^), and tau-PET (β_diagnosis-CN_ = –0.41, *P* < 10^–6^) were elevated and CSF-Aβ_42/40_ (β_diagnosis-CN_ = 0.37, *P* = 0.04) was reduced in CI compared with CN, only tau-PET (β_time_ = 0.047, *P* < 10^–7^; β_time×diagnosis-CN_ = –0.028, *P* = 0.006) showed a significant, but marginal, differentiation in longitudinal trajectories between groups. Expanding this analysis to include all available PET and CSF biomarkers data revealed similar patterns with, again, only tau-PET achieving significant group-level differentiation (Supplemental Figure 4). Full results of these regression analyses are presented in Supplemental Table 4.

**Figure 6:**
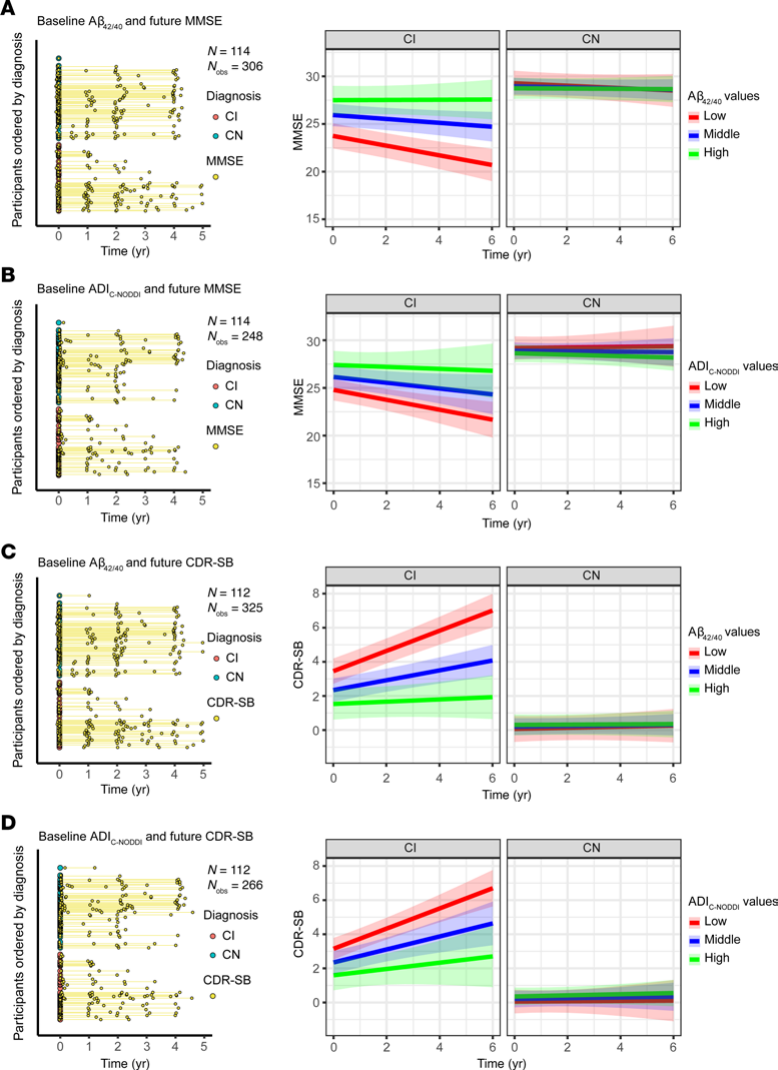
Comparison of baseline CSF Aβ_42/40_ and ADI_C-NODDI_ in predicting longitudinal changes in cognition and dementia risk in CN and CI groups. Low, middle, and high values are the 25th, 50th, and 75th quantiles of the biomarker *z* score. The left panels show the longitudinal distributions of MMSE and CDR-SB anchored at the first CSF collection or dMRI scan of each subject, with CN and CI subjects color-coded. (**A**) Relationship between baseline Aβ_42/40_ levels and prospective MMSE scores, showing that a low Aβ_42/40_ ratio is significantly associated with faster MMSE decline in the CI group, but not in the CN group. (**B**) Relationship between baseline ADI_C-NODDI_ and future MMSE scores, where lower ADI_C-NODDI_ is also linked to faster MMSE decline in the CI group. (**C**) Relationship between baseline Aβ_42/40_ and prospective CDR-SB scores, with lower Aβ_42/40_ ratios significantly predicting a faster increase in CDR-SB in the CI group, but not in the CN group. (**D**) Lower baseline ADI_C-NODDI_ is significantly associated with a faster increase in CDR-SB in the CI group, but not in the CN group. This analysis shows that both the Aβ_42/40_ and ADI_C-NODDI_ biomarkers exhibit similar performances in predicting prospective cognitive and functional decline. Full results are shown in Supplemental Table 5.

**Figure 7:**
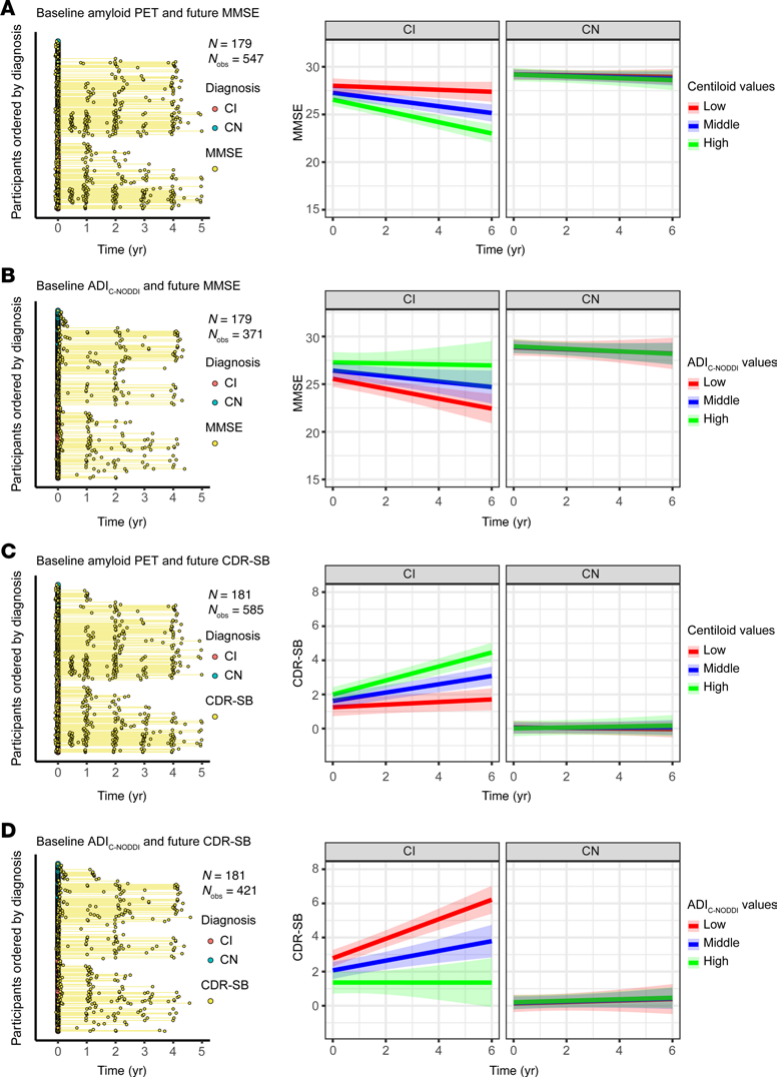
Comparison of baseline amyloid-PET and ADI_C-NODDI_ in predicting longitudinal changes in cognition and dementia risk in CN and CI groups. Low, middle, and high values are the 25th, 50th, and 75th quantiles of the biomarker after Order-Norm transformation. The left panels show the longitudinal distributions of MMSE and CDR-SB anchored at the first PET or dMRI scan of each subject, with CN and CI subjects color-coded. (**A**) Relationship between baseline amyloid-PET levels and prospective MMSE scores, showing that a higher amyloid-PET level is significantly associated with faster MMSE decline in the CI group, but not in the CN group. (**B**) Relationship between baseline ADI_C-NODDI_ and future MMSE scores, where lower ADI_C-NODDI_ is also linked to faster MMSE decline in the CI group. (**C**) Relationship between baseline amyloid-PET and prospective CDR-SB scores, with higher amyloid-PET levels significantly predicting a faster increase in CDR-SB in the CI group, but not in the CN group. (**D**) Lower baseline ADI_C-NODDI_ is significantly associated with a faster increase in CDR-SB in the CI group, but not in the CN group. This analysis shows that both the amyloid-PET and ADI_C-NODDI_ biomarkers exhibit similar performances in predicting prospective cognitive and functional decline. Full results are shown in Supplemental Table 5.

**Figure 8:**
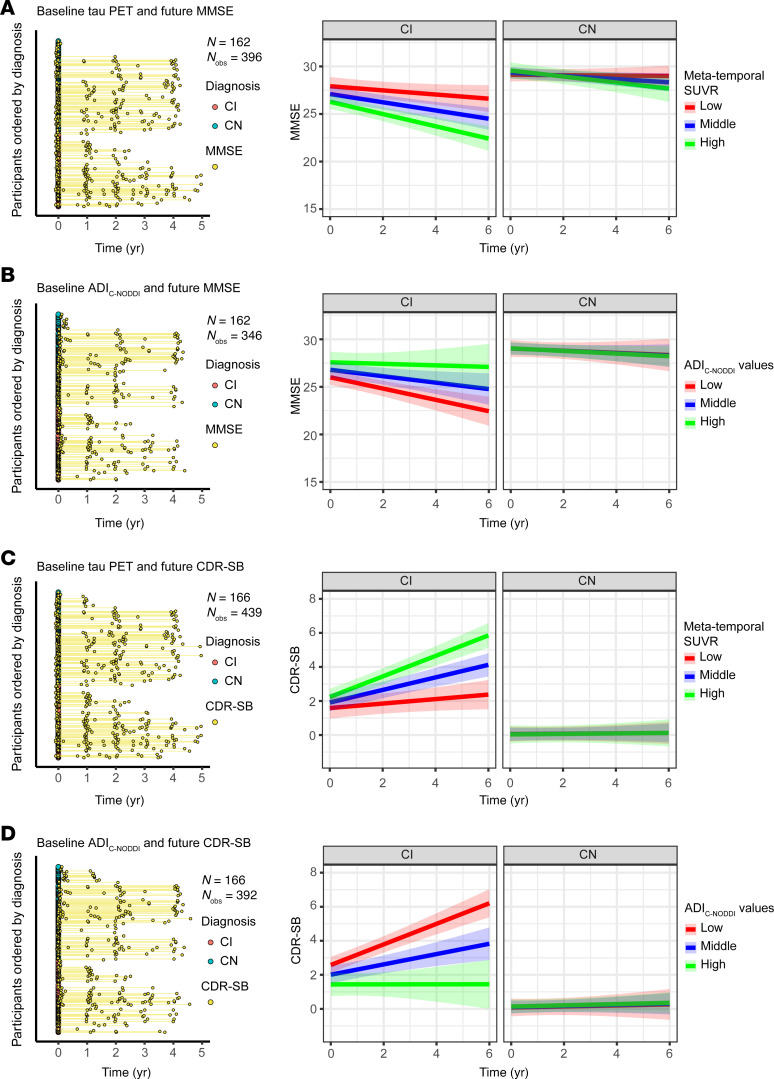
Comparison of baseline tau-PET and ADI_C-NODDI_ in predicting longitudinal changes in cognition and dementia risk in CN and CI groups. Low, middle, and high values are the 25th, 50th, and 75th quantiles of the biomarker after Order-Norm transformation. The left panels show the longitudinal distributions of MMSE and CDR-SB anchored at the first PET or dMRI scan of each subject, with CN and CI subjects color-coded. (**A**) Relationship between baseline tau-PET levels and prospective MMSE scores, showing that a higher tau-PET level is significantly associated with faster MMSE decline in the CI group, but not in the CN group. (**B**) Relationship between baseline ADI_C-NODDI_ and future MMSE scores, where lower ADI_C-NODDI_ is also linked to faster MMSE decline in the CI group. (**C**) Relationship between baseline tau-PET and prospective CDR-SB scores, with higher tau-PET levels significantly predicting a faster increase in CDR-SB in the CI group, but not in the CN group. (**D**) Lower baseline ADI_C-NODDI_ is significantly associated with a faster increase in CDR-SB in the CI group, but not in the CN group. This analysis shows that both the tau-PET and ADI_C-NODDI_ biomarkers exhibit similar performances in predicting prospective cognitive and functional decline. Full results are shown in Supplemental Table 5.

Figure 6, Figure 7, and Figure 8 show that baseline ADI_C-NODDI_, CSF-Aβ_42/40_, amyloid-PET, and tau-PET, the best-performing biomarkers from Figure 5, provided comparable performance in predicting prospective cognitive and functional decline among CI participants: individuals with lower baseline ADI_C-NODDI_ and CSF-Aβ_42/40_ or higher baseline levels of amyloid-PET and tau-PET were significantly associated with greater cognitive and functional decline. Full results of the regression analyses are presented in Supplemental Table 5. Similar results were obtained for CSF and PET biomarkers where the full cohort data were used (Supplemental Figures 5–7).

**Figure 9:**
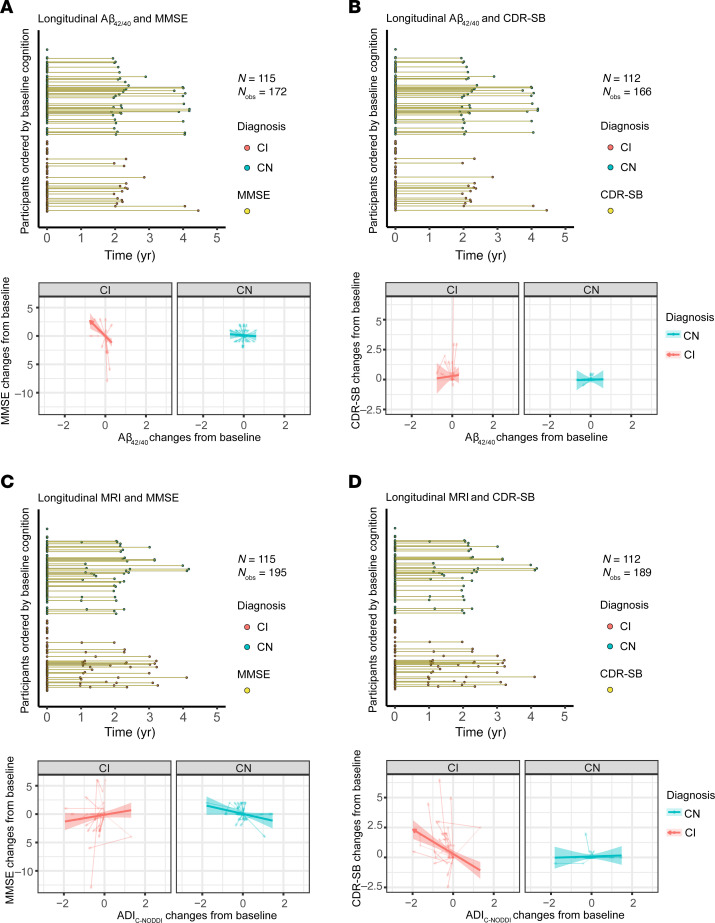
Comparison of the associations between changes from baseline in Aβ_42/40_ or ADI_C-NODDI_ and changes from baseline in MMSE or CDR-SB. (**A** and **B**) The longitudinal distribution of MMSE or CDR-SB scores aligned with the nearest Aβ_42/40_ measurements for linear mixed-effects modeling. Decreases in Aβ_42/40_ are significantly associated with increased MMSE in the CI group but not in the CN group. Changes in CDR-SB were not significantly associated with changes in Aβ_42/40_ in either group. (**C** and **D**) The longitudinal distribution of MMSE or CDR-SB scores aligned with the nearest ADI_C-NODDI_ measurements for linear mixed-effects modeling. While changes in ADI_C-NODDI_ were not significantly associated with changes in MMSE, decreases in ADI_C-NODDI_ were significantly associated with increases in CDR-SB in the CI group. Full results are shown in Supplemental Table 6.

**Figure 10:**
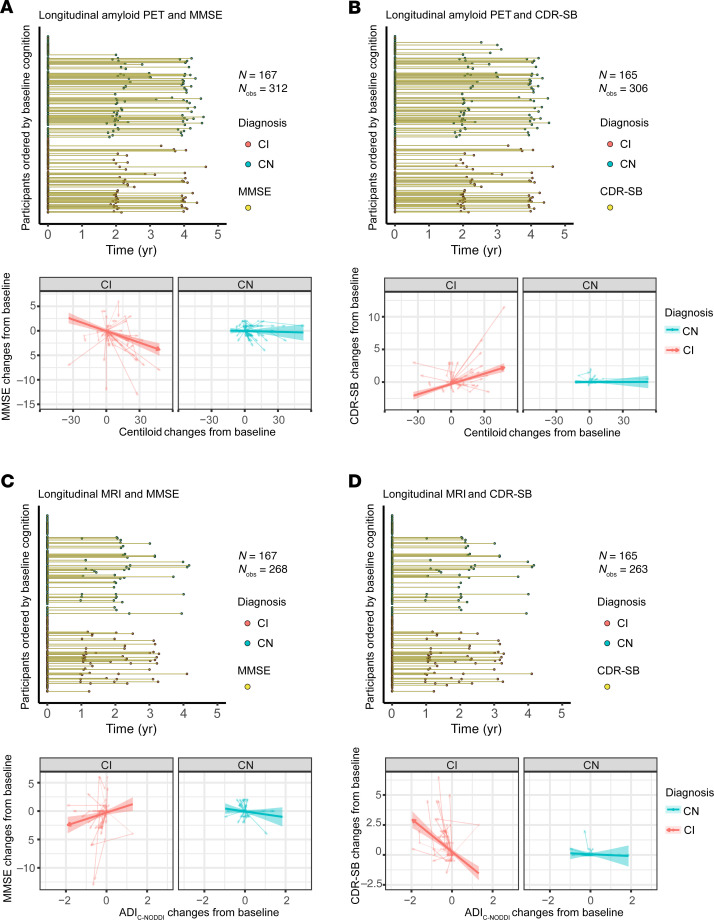
Comparison of the associations between changes from baseline in amyloid-PET or ADI_C-NODDI_ and changes from baseline in MMSE or CDR-SB. (**A** and **B**) The longitudinal distribution of MMSE or CDR-SB scores aligned with the nearest amyloid-PET measurements for linear mixed-effects modeling. Increases in amyloid-PET Centiloid value are significantly associated with decreased MMSE or increased CDR-SB in the CI group but not in the CN group. (**C** and **D**) The longitudinal distribution of MMSE or CDR-SB scores aligned with the nearest ADI_C-NODDI_ measurements for linear mixed-effects modeling. Decreases in ADI_C-NODDI_ were significantly associated with increases in CDR-SB and decreases in MMSE in the CI group. Full results are shown in Supplemental Table 6.

**Figure 11:**
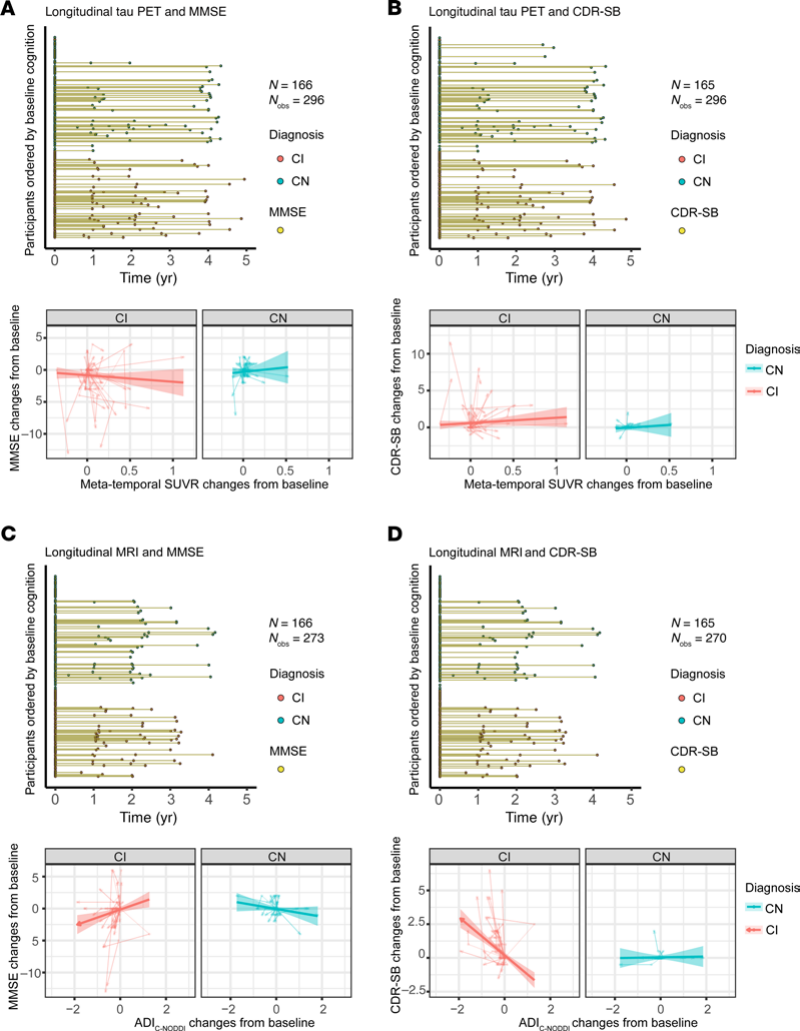
Comparison of the associations between changes from baseline in tau-PET or ADI_C-NODDI_ and changes from baseline in MMSE or CDR-SB. (**A** and **B**) The longitudinal distribution of MMSE or CDR-SB scores aligned with the nearest tau-PET measurements for linear mixed-effects modeling. Changes in tau-PET metatemporal SUVR value were not significantly associated with changes in MMSE or CDR-SB. (**C** and **D**) The longitudinal distribution of MMSE or CDR-SB scores aligned with the nearest ADI_C-NODDI_ measurements for linear mixed-effects modeling. Decreases in ADI_C-NODDI_ were significantly associated with decreases in MMSE and increases in CDR-SB in the CI group. Full results are shown in Supplemental Table 6.

Finally, while the CN group showed minimal association between changes from baseline in ADI_C-NODDI_ and changes from baseline in MMSE or CDR-SB scores, the CI group showed that decreased ADI_C-NODDI_ values are associated with decreased MMSE or increased CDR-SB scores, indicating that axonal degeneration was associated with decreased cognition and function in the CI group (Figure 9, Figure 10, and Figure 11). In contrast, the CI group showed that decreased Aβ_42/40_ values were unexpectedly associated with increased MMSE (β_changes_ = –3.58, *P* = 0.00017) (Figure 9). This is likely due to the variability in CSF collection. However, this unexpected association was drastically attenuated when using the full CSF data set (β_changes_ = –0.824, *P* = 0.016) (Supplemental Figure 8). Furthermore, in both the limited and full datasets, increases in amyloid-PET were significantly associated with decreased MMSE or increased CDR-SB in the CI group (Figure 10 and Supplemental Figure 9). Finally, changes in tau-PET did not exhibit significant associations with changes in MMSE and CDR-SB (Figure 11). However, significant associations were observed when considering the full tau-PET imaging data (β = –1.58, *P* < 10^–6^) (Supplemental Figure 10). Full results of these regression analyses are presented in Supplemental Table 6.

### Sensitivity analysis, excluding AD participants.

As a sensitivity analysis, we repeated the analyses in Figures 2–4 after excluding the 14 participants with a clinical diagnosis of AD from the CI group, therefore restricting the CI group to MCI participants only. Conclusions remained relatively unchanged and can be found in Supplemental Tables 7–9 and Supplemental Figures 11–13.

## Discussion

### Summary of main findings.

This study leveraged longitudinal multishell dMRI data to investigate the role of axonal degeneration in individuals that were CN and CI (including MCI and those with AD). By integrating dMRI, PET imaging, and CSF biomarkers, and cognitive assessments from the ADNI3 study, we report 4 main findings: (a) axonal density/integrity, as measured using ADI, shows a significantly steeper decline in individuals that are CI compared with those serving as CN controls. (b) Baseline ADI is predictive of future cognitive and functional decline. (c) The ADI derived using the C-NODDI model from multishell dMRI outperforms other diffusion models (NODDI and SMI) in predictive accuracy and offers comparable or superior prognostic value relative to PET and CSF biomarkers, while being noninvasive and spatially resolved. (d) Declines in ADI are associated with deterioration in cognitive and functional measures. In contrast, only the amyloid-PET biomarker demonstrated such longitudinal associations.

### Axonal degeneration as a potential core feature of sporadic AD.

Our results provide evidence supporting the role of axonal degeneration in the progression of cognitive and functional impairment in MCI and AD. Postmortem histological studies indicate that WM degeneration, including axonal loss, is a primary consequence of aging and AD, associated with cognitive decline, motor impairments, and neurodegenerative disorders (38). These alterations in WM microstructure have been observed in early-onset autosomal dominant inherited AD occurring years before the symptom onset (9). Such WM alterations are believed to be associated with primary AD pathology and microglia activity in the brain (19). By probing 1 specific component of WM, our work revealed lower axonal density in patients with cognitive impairment, in agreement with previous cross section–based work that used NODDI (39–42). Moreover, axonal density, as measured using our ADI imaging biomarker, predicted future cognitive and functional decline, and longitudinal changes in ADI were also associated with concurrent changes in cognitive performance. These results provide evidence that progressive axonal degeneration is likely a key driver of cognitive deterioration in the early stages of AD and highlight the potential of ADI as a sensitive, noninvasive biomarker for tracking disease progression.

Axonal degeneration is a complex, multistep process that is primarily driven by disruptions in cellular homeostasis, energy deficits, and altered intracellular signaling pathways (43–45). At the molecular level, it involves the breakdown of key components of the axon, such as microtubules and neurofilaments, which are crucial for maintaining structural integrity and efficient axonal transport. Impaired axonal transport, often due to mitochondrial dysfunction or the accumulation of toxic proteins like tau, leads to the accumulation of waste products and energy depletion, further exacerbating degeneration (46–48). Additionally, the activation of signaling pathways, including those mediated by calpains and other proteases, can result in the cleavage of structural proteins and the destabilization of the axonal cytoskeleton, accelerating the process of axonal disintegration (49, 50). These molecular events, which can be triggered by neuroinflammation and oxidative stress, contribute to the progressive loss of axonal function and, ultimately, neuronal death, brain atrophy, and concomitant cognitive and motor dysfunctions. Our results indicate that patients who have a clinical diagnosis of cognitive impairment but also have a greater axonal density are spared from accelerated cognitive and functional decline. These original results underscore the importance of axonal health and call for further investigations into axonal degeneration as a potential core feature of AD.

### Clinical implications and significance.

The findings of this study have important implications for both clinical research and our broader understanding of AD pathophysiology. Detection of axonal degeneration, measured through ADI, prior to significant cognitive decline or overt brain atrophy, underscores its potential as a critical early marker of disease progression. This is particularly relevant for identifying individuals in the prodromal or preclinical stages of AD, when therapeutic interventions are more likely to be effective. One of the most compelling findings is that ADI effectively differentiated longitudinal trajectories between CN and CI individuals, establishing its potential as a monitoring biomarker. This capability will allow clinicians to track disease progression dynamically and identify patients who are undergoing steeper axonal density decline, enabling more timely interventions. Furthermore, our observation that some CI individuals exhibited relatively high ADI levels and slower cognitive decline suggests that axonal density may serve as an indicator of cognitive resilience or reserve. These findings support the use of ADI not only to help with disease monitoring and early diagnosis but also for disease phenotyping and patient stratification. The degree of axonal degeneration could help identify subgroups with distinct clinical trajectories and those more likely to respond to targeted therapies. This has important implications for clinical trial design, as it may improve inclusion criteria, reduce heterogeneity, and increase the likelihood of detecting treatment effects. In terms of intervention, our results reinforce the importance of developing treatments specifically aimed at preserving or restoring axonal integrity, and more broadly, WM integrity. Whether through pharmacological agents, lifestyle modifications (e.g., physical activity, diet), or cognitive training, interventions that enhance or maintain axonal health could delay or mitigate the progression of cognitive symptoms. ADI offers a quantifiable imaging biomarker to monitor the effectiveness of these strategies over time, which could accelerate the development and validation of WM-targeted therapies. Finally, the observation that participants that were CN and MCI had similar CSF biomarker profiles, but significantly different ADI levels, highlights the added value of dMRI-based markers. Higher axonal integrity in the presence of AD pathology may reflect a protective factor, contributing to delayed symptom onset. This supports the notion that ADI could serve as a marker of cognitive reserve and may ultimately guide the development of more personalized, stage-specific treatment strategies. Our results underscore the utility of ADI, particularly when derived using C-NODDI, as a sensitive and clinically meaningful biomarker for early detection, disease monitoring, patient stratification, and therapeutic targeting in AD.

We note that ADI did not significantly predict future cognitive or functional decline in CN participants over the observed time frame. ADI may be most sensitive as a prodromal marker of decline. However, we also acknowledge that the relatively short follow-up period and the inclusion of only nonconverters, who maintained the CN status throughout the study period, likely limited our ability to detect subtle, slow-progressing changes in the unimpaired group. Moreover, while MMSE and CDR-SB are widely used to stage and characterize cognitive impairment, they may not provide sufficient dynamic range to capture early cognitive and functional changes in CN individuals. Further studies with longer follow-up and broader prognostic designs are essential to fully evaluate ADI’s potential as a preclinical biomarker.

### Comparison with CSF and PET biomarkers of AD pathology.

Another major finding of our investigation is that our biomarker of axonal density, ADI_C-NODDI_, provided similar or superior results to PET and CSF biomarkers in differentiating CN from CI as well as predicting cognitive and functional decline. While PET imaging remains a key modality for detecting amyloid and tau pathology with regional specificity, it is limited by high costs, invasiveness, radiation exposure, and relatively low spatial resolution for detecting subtle microstructural changes, particularly in small brain regions. In contrast, MRI allows for direct assessment of microstructural changes in the brain, including myelin damage and axonal loss, which can occur early in the disease and reflect ongoing neurodegeneration (51–54). In contrast, CSF biomarkers, while informative, represent biochemical changes that may occur downstream of initial neurodegenerative events and may not fully capture the complexity of neurodegenerative processes, particularly in the early stages of AD when axonal degeneration may precede significant changes in CSF composition. Although not the focus of current work, dMRI provides high spatial resolution, enabling the detection of topographical patterns of axonal density across different brain regions. These regional patterns are often differentially linked to cognitive decline and disease progression, offering more granular information than CSF biomarkers, which reflect a global concentration of proteins. The ability to capture this detailed, localized information in dMRI allows for more precise differentiation between individuals serving as healthy controls and those with early AD pathology, enhancing its predictive power for cognitive decline and dementia risk, as shown in our and others’ recent work (39, 55). Furthermore, dMRI is noninvasive and easily repeatable, allowing longitudinal monitoring without invasive procedures like lumbar puncture and without radiation exposure associated with PET. The noninvasive nature of dMRI makes it a more patient-friendly and accessible option for repeated assessments, which is advantageous in clinical settings requiring ongoing evaluation. While PET imaging provides valuable topographical insights into brain pathology, its cost, invasiveness, and limited accessibility pose challenges. Finally, while there are growing efforts to develop plasma biomarkers for AD, which offer advantages of accessibility and scalability (56–60), they still require further validation and cannot provide the same anatomical specificity as imaging-based approaches. In this context, ADI_C-NODDI_ could serve as a complementary biomarker, offering spatially resolved, mechanistically informative insights into neurodegeneration. Further work is needed to validate ADI in multicohort data with diverse populations and to explore its integration with PET and fluid biomarkers to enhance early diagnosis, disease staging and phenotyping, and treatment monitoring in AD (32, 39, 55, 61).

### Improving the AT(N) framework.

The AT(N) framework, which categorizes AD biomarkers into amyloid, tau, and neurodegeneration, has proven instrumental in advancing our understanding of the disease’s pathophysiology (6). However, recent studies have begun to emphasize the role of WM integrity, particularly axonal degeneration, as an important factor in the neurodegenerative process (11). While the neurodegeneration component of the AT(N) framework currently focuses on markers such as hippocampal atrophy and CSF NfL, our work suggests that axonal degeneration may play a pivotal role in early disease stages, before significant cortical atrophy becomes evident. Notably, alterations in WM integrity, as measured through established MRI techniques, have been associated with both amyloid and tau pathology (54, 62, 63), providing a potential bridge between the pathological hallmarks of AD and the clinical manifestation of cognitive impairment. Incorporating axonal degeneration, as measured using C-NODDI, into the AT(N) framework could offer new insights into the timing and progression of neurodegeneration, potentially enhancing early detection and therapeutic intervention strategies. Furthermore, combined with protein biomarkers, this imaging biomarker has the potential to improve the AT(N) framework in accurate prediction of disease progression and patient stratification in clinical trials (34, 39, 64).

### Strengths and limitations.

Changes in axonal density were markedly depicted using our C-NODDI dMRI method compared with other state-of-the-art techniques, including NODDI and SMI. While compelling, these biophysical tissue models involve a high-dimensional inverse problem, making derived parameters, especially ADI, sensitive to the impact of experimental conditions and system noise. However, C-NODDI provides physiologically realistic ADI_C-NODDI_ values that exhibit a stronger correlation with the NfL, a plasma biomarker of axonal degeneration (37). As expected, lower ADI_C-NODDI_ values corresponded to higher NfL levels. This technical advancement and our current results underscore the utility of ADI_C-NODDI_ as a powerful, noninvasive, and cost-effective biomarker to help elucidate the mechanisms of axonal degeneration and monitor cognitive decline in MCI and AD.

While our study offers promising insights into the role of axonal degeneration in the AD spectrum and demonstrates the utility of our C-NODDI dMRI method in detecting early axonal changes and their implication in cognitive decline, there are limitations to consider. First, although our results show that C-NODDI–derived ADI performs similarly or superiorly to CSF and PET biomarkers, dMRI still relies on model assumptions that may limit its accuracy in certain populations, particularly in those with advanced disease or comorbid conditions. The sensitivity of our method to detect subtle changes in axonal integrity may also be influenced by the quality of the MRI data, as multishell diffusion imaging is prone to noise and artifacts, which could potentially affect the interpretation of results. Therefore, sophisticated analyses and expertise, as used here, are required to mitigate these potential issues. Furthermore, we only considered whole-brain WM regions in our analysis. However, C-NODDI offers valuable spatial information that can allow for the examination of the spatial distribution of axonal degeneration, even subtle changes, across different regions of the WM, helping to capture early signs of degeneration. Nevertheless, despite focusing on whole-brain WM, C-NODDI was able to depict global variations in axonal integrity, enhancing our understanding of how these changes may be linked to cognitive and functional decline. Future studies focusing on the spatial pattern and involving other cognitive assessments are warranted. Additionally, while our study focuses on longitudinal changes, the relatively short duration of the follow-up period may not fully capture the long-term trajectory of axonal degeneration and its relationship with cognitive and functional decline, particularly in the preclinical stage of AD. With the continuing progress of the ADNI study, we hope to enhance our results when future follow-up visits become available. Furthermore, the generalizability of our findings may be limited by the sample size, and the specific cohort used may not fully represent the broader spectrum of AD and other neurodegenerative diseases. Moreover, male participants had a longer follow-up than female ones; therefore, a bias toward male participants may have influenced the overall results of this study. Lastly, while dMRI offers a noninvasive and repeatable means of tracking disease progression, it remains resource-intensive compared with CSF biomarkers, requiring specialized equipment and expertise. However, we remain hopeful about the expanding accessibility of dMRI to the general population with the technical advancement in low-field portable MRI. Despite these limitations, our original findings highlight the implication of axonal degeneration in AD and the potential of C-NODDI to provide valuable insights into the early stages of AD, underscoring the need for further validation in larger, more diverse cohorts with extended follow-up periods.

## Methods

### Sex as a biological variable.

Our study included both male and female participants. Sex and age were used as covariates in all statistical analyses. The statistical results for all covariates can be found in Supplemental Tables 1–9.

### Participants.

Participants were drawn from the ADNI database (https://adni.loni.usc.edu/). The ADNI initiative was started in 2003 under Michael W. Weiner and was a private–public partnership funded by private companies as well as the NIH and the National Institute on Aging (NIA) for the purpose of developing clinical, imaging, genetic, and biochemical biomarkers for early detection of AD. Inclusion criteria were participants who had multishell diffusion scans on Siemens scanners. Baseline and longitudinal dMRI scans were obtained across all available participants. A subset of these participants also had CSF fluid biomarkers. For more information about the participants, see https://adni.loni.usc.edu/data-samples/adni-data/

### dMRI imaging and processing.

After accessing the ADNI3 multishell diffusion dataset, we applied the following inclusion criteria: scan description listed as “axial MB DTI” and acquisition performed on Siemens scanners. A total of 357 scans met these criteria, while only 9 scans acquired on Philips scanners were excluded. The Philips scans were excluded to minimize scanner-related bias and to avoid potential challenges associated with data harmonization. The included scans span acquisition dates from June 5, 2017 to October 20, 2022. Each participant underwent whole-brain dMRI scans using a 3T Siemens scanner. MAGNETOM Skyra, MAGNETOM Prisma, and MAGNETOM Prisma^fit^ were used depending on the scanning site. Multishell dMRI data were collected with a repetition time of 3,400 ms and an echo time of 71 ms. Each scan included 127 separate diffusion-weighted images: 13 with *b* = 0 s/mm^2^, 6 with *b* = 500 s/mm^2^, 48 with *b* = 1,000 s/mm^2^, and 60 with *b* = 2,000 s/mm^2^. The dMRI scans were preprocessed. First, the raw imaging dataset was processed in Python to convert individual DICOM images into a common 4D NIfTI format. Images were subsequently denoised using the MP-PCA MATLAB toolbox. Brain extraction and linear registration of each diffusion-weighted image to the b_0_ image were performed using the FMRIB Software Library (FSL) (65). Eddy current–induced distortions and participant movements were corrected using FSL, and ADI maps were then derived using the NODDI, C-NODDI, or SMI models (35–37). Lastly, derived parameter maps were registered to the JHU-ICBM-T_2_-2mm template. WM voxels were defined based on a threshold of a WM mask with a probability higher than 95%. The whole-brain WM ADI values were then calculated.

### PET imaging and processing.

We obtained processed PET imaging summaries in tabular format from the ADNI site. Amyloid-PET data were obtained from the UC Berkeley – Amyloid PET 6mm Res analysis [ADNI1,GO,2,3,4] file, and tau-PET data were obtained from the UC Berkeley – Tau PET PVC 6mm Res analysis [ADNI2,3,4] file. Because amyloid-PET scans were acquired with 2 different tracers, we selected Centiloid values as the region of interest. For the tau-PET dataset, which involved a single tracer, we selected the metatemporal standardized uptake value ratio (SUVR) as the region of interest. These regions are commonly used in PET imaging analyses. Detailed processing methods for PET imaging can be found on the ADNI website (https://adni.loni.usc.edu/data-samples/adni-data/neuroimaging/pet/).

### CSF biomarkers.

Lumbar CSF samples were collected and stored at –80°C at the ADNI Biomarker Core at the University of Pennsylvania School of Medicine. The established CSF biomarkers of Aβ ratio (Aβ_42/40_), total tau protein (tau), and phosphorylated-tau (pTau_181_) were obtained from a subset of ADNI participants who had multishell dMRI scans. All CSF biomarker values were obtained on the ADNI site from the UPENN CSF Biomarkers Roche Elecsys [ADNI1,GO,2,3] CSV file, and detailed assay methods can be found in the ADNI file UPENN CSF Biomarkers Roche Elecsys Methods [ADNI1,GO,2,3]. Further collection details can be found through ADNI Documentation (https://adni.loni.usc.edu/help-faqs/adni-documentation/).

### Cognitive scores.

Detailed inclusion and diagnostic criteria for each diagnosis group can be found in the ADNI3 Protocol (https://adni.loni.usc.edu/wp-content/themes/freshnews-dev-v2/documents/consentForms/ADNI3_ProtocolVersion3.1_20201204.pdf). The MMSE and CDR-SB scores were used in this study to assess global cognition and dementia risk over time. MMSE and CDR-SB values were drawn from the ADNIMERGE – Key ADNI tables merged into one table [ADNI1,GO,2,3] file. Further information on the cognitive assessment can be found on the ADNI site (https://adni.loni.usc.edu/data-samples/adni-data/clinical-assessments/).

### Statistics.

All statistical analyses were conducted using RStudio 4.3. Linear mixed-effects regression models were used to analyze longitudinal multishell dMRI, PET, CSF, and cognitive data, with the following primary objectives: (a) to examine longitudinal changes in axonal density, as measured by ADI_NODDI_, ADI_C-NODDI_, or ADI_SMI_; (b) to investigate whether baseline ADI predicts future changes in cognitive function, as assessed by MMSE and CDR-SB scores; and (c) to assess the association between longitudinal changes from baseline in ADI and longitudinal changes from baseline in cognitive/functional outcomes, specifically MMSE and CDR-SB scores.

Secondary analyses were conducted using CSF biomarkers (Aβ_42/40_, total tau, and pTau_181_) and PET biomarkers (amyloid-PET and tau-PET), available for a subset of participants who had both multishell dMRI data and CSF or PET measures. To ensure fair comparisons across fluid and imaging modalities, these analyses were restricted to participants with complete multishell dMRI data and a maximum follow-up duration of 5 years. For simplicity and based on prior results indicating superior performance, the comparison was limited to CSF Aβ_42/40_, amyloid-PET, tau-PET, and ADI_C-NODDI_.

To further evaluate the discriminatory power of CSF and PET biomarkers in differentiating longitudinal trajectories between the CN and CI groups, we repeated analysis 1 using the full dataset of CSF and PET biomarkers available at the time. Similarly, the full data set was used to rerun analyses 2 and 3 to further assess the predictive power of CSF and PET biomarkers of cognitive and functional decline and the association between changes in CSF or PET biomarkers and changes in MMSE or CDR-SB scores. These analyses were necessary to support the subsequent discussion.

To ensure that findings were not solely driven by the inclusion of AD patients, analyses were repeated after excluding AD patients from the CI group. Additionally, to explore whether axonal integrity contributes to cognitive reserve, baseline differences between the CN and MCI groups were compared across each CSF and PET biomarker, as well as ADI_C-NODDI_, adjusting for age and sex. Results are presented in Supplemental Tables S7–S9.

All models included interaction terms with diagnostic group (CN vs. CI) to test for group differences, while controlling for relevant covariates, namely, age and sex. Detailed model specifications are provided in the corresponding figure legends. For all tests, an uncorrected *P* value of less than 0.05 was considered significant.

### Study approval.

All participants signed consent forms, and the study design was approved by the IRB of the NIA. Institutional approvals and study governance information are available through the ADNI data access portal: https://adni.loni.usc.edu/data-samples/adni-data/#AccessData

### Data availability.

Data values reported in this manuscript are provided in the Supporting Data Values file. Analysis codes for each figure are available from GitHub (https://github.com/mrpadunit/JCI_paper_Early_axonal_degeneration; commit ID 52798217ed0ba257604b81339d20ccbe8729d620). Tabular data from the ADNI can be directly accessed from the site with approval from the ADNI committee. Data used in preparation of this article were obtained from the ADNI database (https://adni.loni.usc.edu).

## Author contributions

ZG conceived the study, designed the study, performed experiments and analyses, and wrote and edited the manuscript. JPL, M Bilgel, and AYG performed experiments, helped with data analyses, and edited the manuscript. JB, NYF, ADR, NZ, and AT edited the manuscript. RDC, JME, LF provided intellectual discussion and edited the manuscript. M Bouhrara conceived the study, designed experiments, and wrote and edited the manuscript. All authors provided comments on the final manuscript.

## Funding support

This work is the result of NIH funding, in whole or in part, and is subject to the NIH Public Access Policy. Through acceptance of this federal funding, the NIH has been given a right to make the work publicly available in PubMed Central. Private sector contributions to the ADNI are facilitated by the Foundation for the National Institutes of Health (www.fnih.org).

- NIH, Intramural Research Program.
- NIH, U01 AG024904 to the ADNI, for data collection and sharing.
- Department of Defense, W81XWH-12-2-0012 to the ADNI, for data collection and sharing.

Funding to the ADNI (all following funders):

- NIA.
- National Institute of Biomedical Imaging and Bioengineering.
- AbbVie.
- Alzheimer’s Association.
- Alzheimer’s Drug Discovery Foundation.
- Araclon Biotech.
- BioClinica Inc.
- Biogen.
- Bristol-Myers Squibb.
- CereSpir Inc.
- Cogstate.
- Eisai Inc.
- Elan Pharmaceuticals Inc.
- Eli Lilly and Company.
- EuroImmun.
- F. Hoffmann-La Roche Ltd. and its affiliated company Genentech Inc.
- Fujirebio.
- GE Healthcare.
- IXICO Ltd.
- Janssen Alzheimer Immunotherapy Research & Development LLC.
- Johnson & Johnson Pharmaceutical Research & Development LLC.
- Lumosity.
- Lundbeck.
- Merck & Co. Inc.
- Meso Scale Diagnostics LLC.
- NeuroRx Research.
- Neurotrack Technologies.
- Novartis Pharmaceuticals Corporation.
- Pfizer Inc.
- Piramal Imaging.
- Servier.
- Takeda Pharmaceutical Company.
- Transition Therapeutics.
- Canadian Institutes of Health Research, for support of ADNI clinical sites in Canada.

## Supplementary Material

Supplemental data

ICMJE disclosure forms

Supporting data values

## Figures and Tables

**Table 1:**
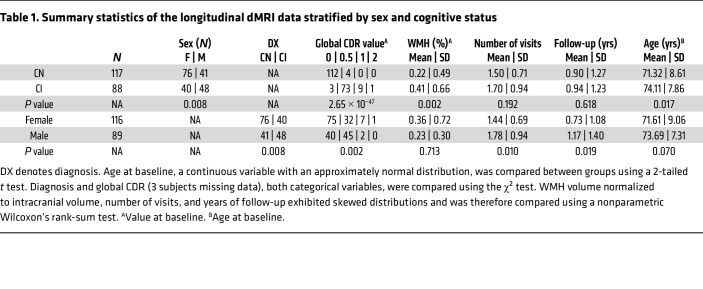
Summary statistics of the longitudinal dMRI data stratified by sex and cognitive status

## Version 1 11/27/2025

In-Press Preview

## Version 2 02/02/2026

Electronic publication
