# Supplementary material for: Early axonal degeneration linked to clinical decline in Alzheimer’s disease progression revealed with diffusion MRI
Source: J Clin Invest. 2025 Nov 27;136(3):e196638. doi: 10.1172/JCI196638 (PMC12867157; doi:10.1172/JCI196638)
Supplement: Supplemental data [file jci-136-196638-s229.pdf]

Supplementary Material

Early axonal degeneration linked to clinical decline in Alzheimer's disease progression revealed with diffusion MRI

Zhaoyuan Gong, John P. Laporte, Alexander Y. Guo, Murat Bilgel, Jonghyun Bae, Noam Y. Fox, Angelique de Rouen, Nathan Zhang, Aaliya Taranath, Rafael de Cabo, Josephine M. Egan, Luigi Ferrucci, Mustapha Bouhrara, and *for the Alzheimer's Disease Neuroimaging Initiative*

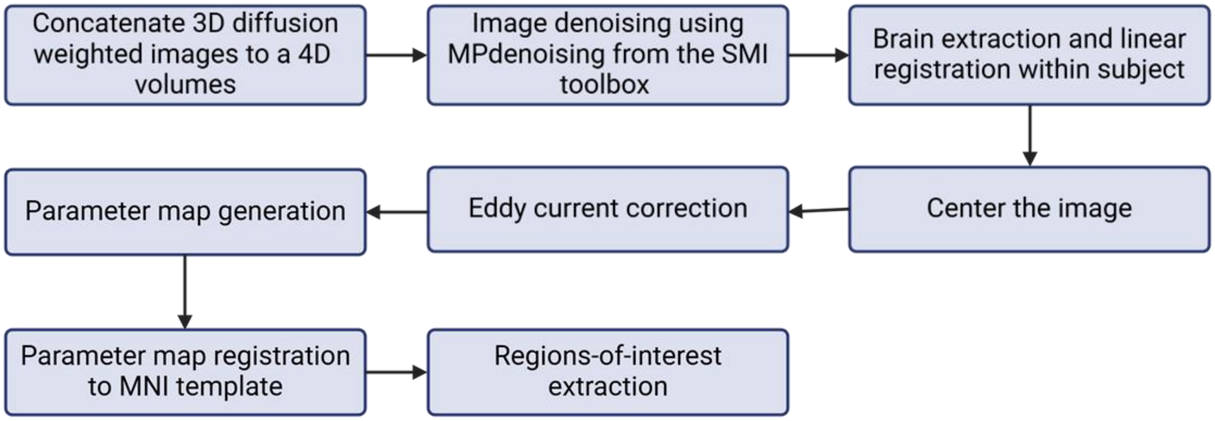

Figure S1. Flowchart of the diffusion magnetic resonance imaging (dMRI) processing pipeline. After downloading DICOM files from the ADNI site, 3D dMRI were concatenated into a 4D volume. Image denoising was performed using the *MPdenoising.m* MATLAB function from https://github.com/NYU-DiffusionMRI/mppca_denoise. Brain extraction was performed on the dMRI obtained at b = 0 s^2/mm (aka., b0 image), and all dMRI were brain-masked and linearly registered to the corresponding b0 image. To facilitate later nonlinear registration to the MNI template, all images were also linearly transformed to center the field of view. Eddy current correction was applied using the *eddy* command in FSL. Due to the lack of reverse phase encoding data, susceptibility-induced off-resonance fields were not corrected. Preprocessed data were subsequently fitted to three biophysical diffusion models (NODDI, C-NODDI, and SMI) to derive the corresponding Axonal Density Indeces (ADIs). For each model, the resulting ADI map wes nonlinearly registered to the MNI template using FSL, and the whole brain white matter region-of-interest (ROI) value was then calculated.

Table S1. Linear mixed-effects model results for Figure 2.

The model equation is specified as:

$$\mathrm{ADI}_{\mathrm{ij}} \sim \beta_0 + \beta_{\mathrm{Age}} \times \mathrm{Age}_{\mathrm{i}} + \beta_{\mathrm{sex}} \times \mathrm{Sex}_{\mathrm{i}} + \beta_{\mathrm{Time}} \times \mathrm{Time}_{\mathrm{ij}} + \beta_{\mathrm{Diagnosis}} \times \mathrm{Diagnosis}_{\mathrm{i}} + \beta_{\mathrm{Time\times Diagnosis}} \times \mathrm{Time}_{\mathrm{ij}} \times \mathrm{Diagnosis}_{\mathrm{i}} + \mathrm{b}_{\mathrm{i}} + \epsilon_{\mathrm{ij}},$$

where $\mathrm{ADI}_{\mathrm{ij}}$ represents the longitudinal ADI value for subject i at time point j; $\mathrm{Age}_{\mathrm{i}}$ is the age at the first MRI measurement for subject i; $\mathrm{Sex}_{\mathrm{i}}$ denotes subject sex; $\mathrm{Time}_{\mathrm{ij}}$ is the time since the first MRI measurement for subject i at time point j; $\mathrm{Diagnosis}_{\mathrm{i}}$ indicates the cognitive diagnosis either as cognitive normal (CN) or cognitively impaired (CI, which includes MCI and AD); $\mathrm{Time}_{\mathrm{ij}} \times \mathrm{Diagnosis}_{\mathrm{i}}$ is the interaction term representing differential time trajectories of ADI for two diagnosis groups; b_{i} is the random intercept for subject i; ϵ_{ij} is the residual error. $\mathrm{Age}_{\mathrm{i}}$ was standardized using baseline mean and standard deviation, while $\mathrm{Time}_{\mathrm{ij}}$ is in the original unit (yrs.) for easier interpretation. Whole brain white matter $\mathrm{ADI}_{\mathrm{NODDI}}$, $\mathrm{ADI}_{\mathrm{C\text{-}NODDI}}$, and $\mathrm{ADI}_{\mathrm{SMI}}$ values were used. Full statistical results are presented below.

	$\mathrm{ADI}_{\mathrm{NODDI}}$ trajectory				
	Estimate	Std. Error	df	t value	Pr(>|t|)
(Intercept)	-4.21e-01	1.22e-01	2.07e+02	-3.43e+00	7.17e-04
Age	-2.64e-01	6.58e-02	2.06e+02	-4.01e+00	8.39e-05
Time	-1.80e-01	2.69e-02	1.26e+02	-6.68e+00	6.78e-10
Diagnosis-CN	4.44e-01	1.35e-01	2.09e+02	3.30e+00	1.15e-03
Sex-Male	3.95e-01	1.33e-01	2.02e+02	2.98e+00	3.27e-03
Diagnosis-CN × Time	1.19e-01	3.57e-02	1.27e+02	3.35e+00	1.07e-03

	ADI$_{C\text{-}NODDI}$ trajectory				
	Estimate	Std. Error	df	t value	Pr(>|t|)
(Intercept)	-3.56e-01	1.08e-01	2.14e+02	-3.31e+00	1.10e-03
Age	-4.86e-01	5.78e-02	2.11e+02	-8.40e+00	6.57e-15
Time	-2.08e-01	3.30e-02	1.37e+02	-6.31e+00	3.60e-09
Diagnosis-CN	6.29e-01	1.19e-01	2.18e+02	5.30e+00	2.89e-07
Sex-Male	3.48e-04	1.16e-01	2.04e+02	3.00e-03	9.98e-01
Diagnosis-CN × Time	1.81e-01	4.37e-02	1.38e+02	4.13e+00	6.18e-05

	ADI$_{SMI}$ trajectory				
	Estimate	Std. Error	df	t value	Pr(>|t|)
(Intercept)	-1.48e-01	1.32e-01	2.22e+02	-1.12e+00	2.63e-01
Age	-1.62e-01	7.05e-02	2.16e+02	-2.30e+00	2.24e-02
Time	1.05e-01	5.20e-02	1.52e+02	2.02e+00	4.55e-02
Diagnosis-CN	2.17e-01	1.46e-01	2.30e+02	1.49e+00	1.38e-01
Sex-Male	-1.22e-02	1.41e-01	2.03e+02	-8.70e-02	9.31e-01
Diagnosis-CN × Time	-7.04e-02	6.88e-02	1.53e+02	-1.02e+00	3.08e-01

Diagnosis was referenced to the CI group, and sex was referenced to females. P-values were not corrected for multiple comparisons.

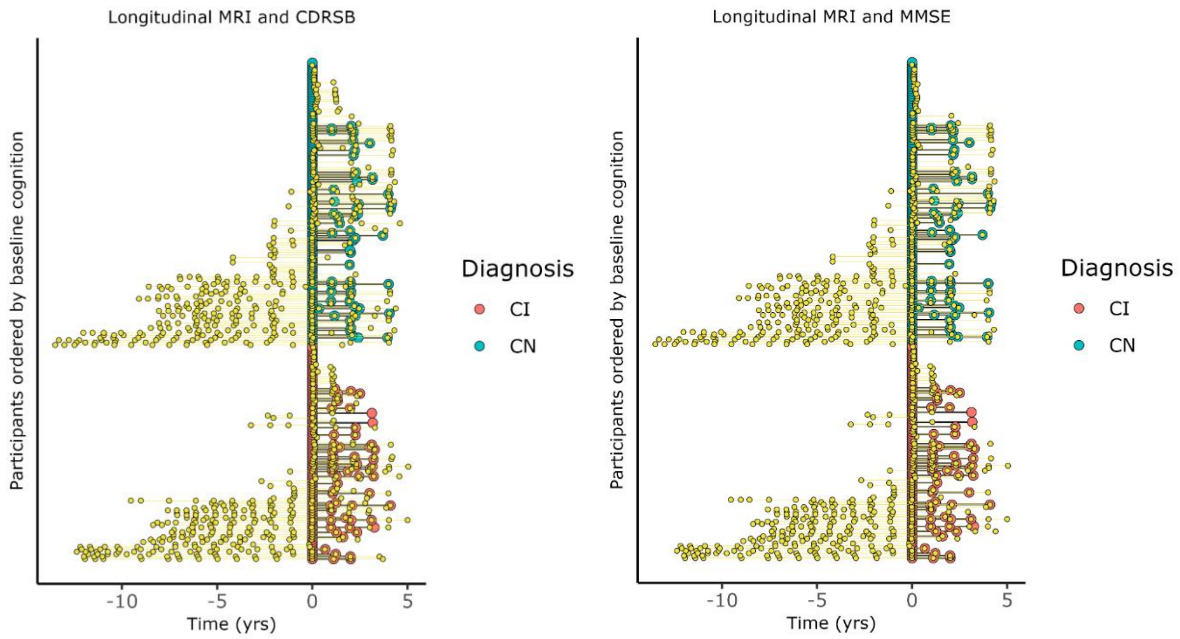

Figure S2. Available longitudinal diffusion MRI (dMRI) measurements and longitudinal cognitive measurements. Cognitive scores (MMSE or CDR-SB) that occurred after the MRI measurements, or within 0.1 years prior to the MRI measurements, were retained for the analyses presented in the manuscript Figure 3.

Table S2. Linear mixed-effects model results for Figure 3.

The model equation is specified as:

$$\mathrm{MMSE/CDR\text{-}SB}_{ij} \sim \beta_0 + \beta_{Age} \times \mathrm{Age}_i + \beta_{sex} \times \mathrm{Sex}_i + \beta_{Time} \times \mathrm{Time}_{ij} + \beta_{Diagnosis} \times \mathrm{Diagnosis}_i + \beta_{ADI} \times \mathrm{ADI}_i + \beta_{Time\times ADI} \times \mathrm{Time}_{ij} \times \mathrm{ADI}_i + \beta_{Time\times Diagnosis} \times \mathrm{Time}_{ij} \times \mathrm{Diagnosis}_i + \beta_{ADI\times Diagnosis} \times \mathrm{ADI}_i \times \mathrm{Diagnosis}_i + \beta_{Time\times Diagnosis\times ADI} \times \mathrm{Time}_{ij} \times \mathrm{Diagnosis}_i \times \mathrm{ADI}_i + b_i + \epsilon_{ij},$$

where $\mathrm{MMSE/CDR\text{-}SB}_{ij}$ represents the longitudinal MMSE or CDR-SB scores for subject i at time point j; Age_i is the age at the baseline MRI measurement for subject i; Sex_i denotes the subject sex; Time_{ij} is the time of cognitive assessment since the baseline MRI measurement for subject i at time point j; $\mathrm{Diagnosis}_i$ indicates the cognitive diagnosis either as cognitive normal (CN) or cognitively impaired (CI, includes MCI and AD) for subject i; and ADI_i is the baseline axonal density index. The model includes all two-way interactions among Time_{ij}, $\mathrm{Diagnosis}_i$, and ADI_i, as well as the three-way interaction term. b_i is the random intercept for subject i and ϵ_{ij} is the residual error. Age_i and ADI_i were standardized using baseline mean and standard deviation, while Time_{ij} was kept in the original unit (yrs.) for easier interpretation. Whole brain white matter $\mathrm{ADI}_{\mathrm{NODDI}}$, $\mathrm{ADI}_{\mathrm{C\text{-}NODDI}}$, and $\mathrm{ADI}_{\mathrm{SMI}}$ values were used. Full statistical results are presented below.

	$\mathrm{ADI}_{\mathrm{NODDI}}$ predicts prospective MMSE				
	Estimate	Std. Error	df	t value	Pr(>|t|)
(Intercept)	2.63e+01	4.16e-01	2.12e+02	6.32e+01	2.20e-139
Age	-1.39e-01	2.21e-01	1.99e+02	-6.30e-01	5.29e-01
Sex-Male	3.44e-01	4.35e-01	1.86e+02	7.91e-01	4.30e-01
Diagnosis-CN	2.68e+00	4.56e-01	2.26e+02	5.88e+00	1.46e-08
Time	-5.95e-01	1.29e-01	2.32e+02	-4.63e+00	6.18e-06
Whole brain ADI	5.99e-01	3.08e-01	2.22e+02	1.94e+00	5.33e-02
Diagnosis-CN × Time	4.90e-01	1.57e-01	2.31e+02	3.11e+00	2.10e-03
Diagnosis-CN × ADI	-5.96e-01	4.46e-01	2.23e+02	-1.33e+00	1.83e-01
Time × ADI	-6.29e-02	1.32e-01	2.37e+02	-4.77e-01	6.34e-01
Diagnosis-CN × Time × ADI	4.39e-02	1.65e-01	2.33e+02	2.67e-01	7.90e-01

	ADI$_{C-NODDI}$ predicts prospective MMSE				
	Estimate	Std. Error	df	t value	Pr(>|t|)
(Intercept)	2.66e+01	4.04e-01	2.15e+02	6.58e+01	2.96e-144
Age	5.27e-02	2.36e-01	1.92e+02	2.23e-01	8.23e-01
Sex-Male	5.19e-01	4.05e-01	1.82e+02	1.28e+00	2.02e-01
Diagnosis-CN	2.30e+00	4.57e-01	2.27e+02	5.04e+00	9.57e-07
Time	-4.23e-01	1.39e-01	2.39e+02	-3.04e+00	2.62e-03
Whole brain ADI	1.23e+00	3.35e-01	2.21e+02	3.68e+00	2.93e-04
Diagnosis-CN × Time	3.24e-01	1.70e-01	2.36e+02	1.91e+00	5.71e-02
Diagnosis-CN × ADI	-1.09e+00	4.56e-01	2.25e+02	-2.39e+00	1.77e-02
Time × ADI	3.17e-01	1.48e-01	2.42e+02	2.15e+00	3.27e-02
Diagnosis-CN × Time × ADI	-3.52e-01	1.80e-01	2.39e+02	-1.96e+00	5.13e-02

	ADI$_{SMI}$ predicts prospective MMSE				
	Estimate	Std. Error	df	t value	Pr(>|t|)
(Intercept)	2.61e+01	4.10e-01	2.12e+02	6.37e+01	4.22e-140
Age	-2.07e-01	2.16e-01	1.99e+02	-9.60e-01	3.38e-01
Sex-Male	3.75e-01	4.24e-01	1.86e+02	8.84e-01	3.78e-01
Diagnosis-CN	2.89e+00	4.51e-01	2.26e+02	6.40e+00	8.78e-10
Time	-5.75e-01	1.34e-01	2.34e+02	-4.29e+00	2.64e-05
Whole brain ADI	-4.15e-01	3.67e-01	2.32e+02	-1.13e+00	2.58e-01
Diagnosis-CN × Time	4.78e-01	1.62e-01	2.32e+02	2.96e+00	3.39e-03
Diagnosis-CN × ADI	4.09e-01	4.59e-01	2.32e+02	8.92e-01	3.74e-01
Time × ADI	2.44e-02	1.32e-01	2.32e+02	1.85e-01	8.54e-01
Diagnosis-CN × Time × ADI	-1.04e-01	1.59e-01	2.31e+02	-6.54e-01	5.14e-01

	ADI$_{NODDI}$ predicts prospective CDR-SB				
	Estimate	Std. Error	df	t value	Pr(>|t|)
(Intercept)	2.31e+00	2.51e-01	2.18e+02	9.22e+00	2.57e-17
Age	1.22e-01	1.30e-01	1.95e+02	9.38e-01	3.50e-01
Sex-Male	-3.16e-01	2.60e-01	1.89e+02	-1.22e+00	2.24e-01
Diagnosis-CN	-2.20e+00	2.73e-01	2.33e+02	-8.05e+00	4.20e-14
Time	4.78e-01	7.14e-02	2.81e+02	6.69e+00	1.23e-10
Whole brain ADI	-5.77e-01	1.86e-01	2.28e+02	-3.11e+00	2.10e-03
Diagnosis-CN × Time	-4.34e-01	8.70e-02	2.80e+02	-4.99e+00	1.07e-06
Diagnosis-CN × ADI	7.04e-01	2.64e-01	2.32e+02	2.66e+00	8.25e-03
Time × ADI	-3.69e-02	6.96e-02	2.87e+02	-5.30e-01	5.97e-01
Diagnosis-CN × Time × ADI	2.28e-02	8.74e-02	2.83e+02	2.61e-01	7.94e-01

	ADI$_{C-NODDI}$ predicts prospective CDR-SB				
	Estimate	Std. Error	df	t value	Pr(>|t|)
(Intercept)	2.10e+00	2.37e-01	2.26e+02	8.85e+00	2.49e-16
Age	-2.01e-02	1.35e-01	1.92e+02	-1.49e-01	8.82e-01
Sex-Male	-4.67e-01	2.33e-01	1.89e+02	-2.01e+00	4.64e-02
Diagnosis-CN	-1.94e+00	2.66e-01	2.38e+02	-7.29e+00	4.63e-12
Time	2.99e-01	7.63e-02	2.88e+02	3.92e+00	1.13e-04
Whole brain ADI	-1.02e+00	1.98e-01	2.34e+02	-5.14e+00	5.78e-07
Diagnosis-CN × Time	-2.57e-01	9.18e-02	2.86e+02	-2.80e+00	5.41e-03
Diagnosis-CN × ADI	1.04e+00	2.63e-01	2.39e+02	3.97e+00	9.41e-05
Time × ADI	-3.72e-01	8.29e-02	2.92e+02	-4.48e+00	1.06e-05
Diagnosis-CN × Time × ADI	3.77e-01	9.94e-02	2.89e+02	3.79e+00	1.81e-04

	ADI_{SMI} predicts prospective CDR-SB				
	Estimate	Std. Error	df	t value	Pr(>|t|)
(Intercept)	2.45e+00	2.52e-01	2.17e+02	9.74e+00	7.87e-19
Age	1.53e-01	1.30e-01	1.94e+02	1.18e+00	2.40e-01
Sex-Male	-3.45e-01	2.59e-01	1.88e+02	-1.33e+00	1.84e-01
Diagnosis-CN	-2.31e+00	2.77e-01	2.31e+02	-8.35e+00	6.30e-15
Time	5.02e-01	7.21e-02	2.81e+02	6.96e+00	2.34e-11
Whole brain ADI	-2.85e-02	2.22e-01	2.30e+02	-1.28e-01	8.98e-01
Diagnosis-CN × Time	-4.64e-01	8.77e-02	2.79e+02	-5.29e+00	2.45e-07
Diagnosis-CN × ADI	5.57e-02	2.78e-01	2.33e+02	2.00e-01	8.42e-01
Time × ADI	1.35e-02	7.84e-02	2.78e+02	1.72e-01	8.64e-01
Diagnosis-CN × Time × ADI	1.53e-02	9.27e-02	2.79e+02	1.65e-01	8.69e-01

Diagnosis was referenced to the CI group, and sex was referenced to females. P-values were not corrected for multiple comparisons.

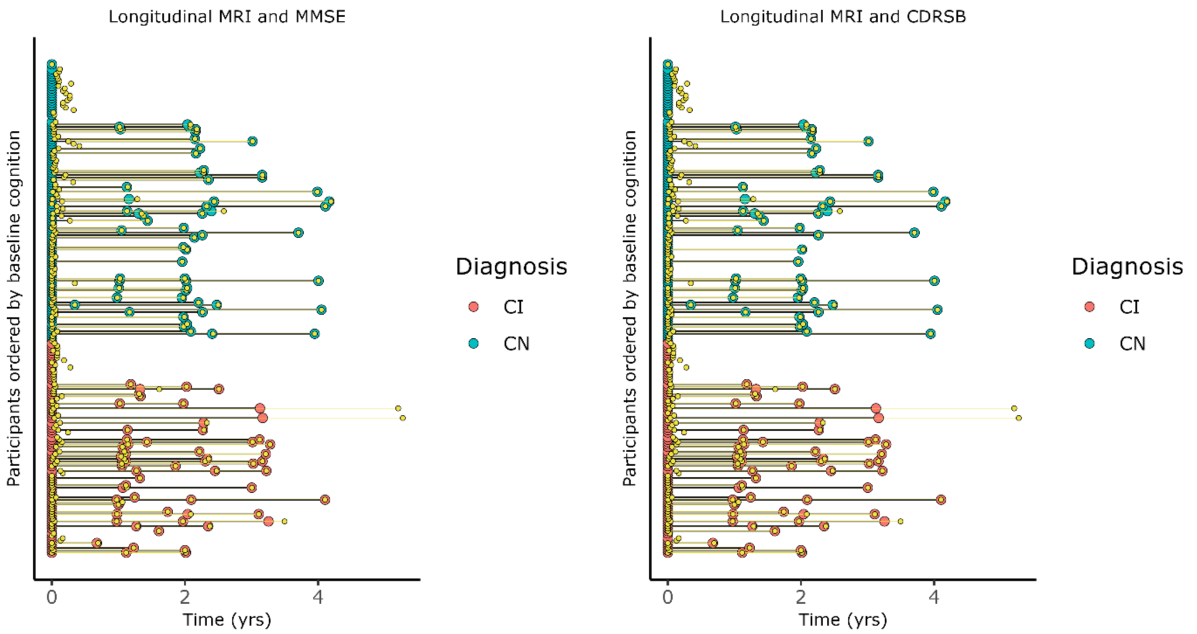

Figure S3. Available longitudinal diffusion MRI (dMRI) measurements and corresponding cognitive measurements (CDR-SB and MMSE). The closest MMSE or CDR-SB measurement to each dMRI measurement was retained for analysis. As visually evident, most cognitive assessments were conducted at or near the time of the MRI measurement. For analysis in Figure 4, the timing of each MMSE or CDR-SB measurement was shifted to align with its nearest dMRI measurement, as outlined in the main manuscript.

Table S3. Linear mixed-effects model results for Figure 4.

The model equation is specified as:

$$\text{MMSE/CDR-SB changes}_{\text{ij}} \sim \beta_0 + \beta_{\text{sex}} \times \text{Sex}_{\text{i}} + \beta_{\text{Age}} \times \text{Age}_{\text{ij}} + \beta_{\text{Diagnosis}} \times \text{Diagnosis}_{\text{i}} + \beta_{\text{ADI changes}} \times \text{ADI changes}_{ij} + \beta_{\text{Diagnosis}\times\text{ADI changes}} \times \text{Diagnosis}_{\text{i}} \times \text{ADI changes}_{ij} + \beta_{\text{Baseline ADI}} \times \text{Baseline ADI}_{\text{i}} + \beta_{\text{Diagnosis}\times\text{Baseline ADI}} \times \text{Diagnosis}_{\text{i}} \times \text{Baseline ADI}_{\text{i}} + \text{b}_{\text{i}} + \epsilon_{\text{ij}},$$

where $\text{MMSE/CDR-SB changes}_{\text{ij}}$ is the longitudinal MMSE or CDR-SB changes for subject i at time point j from subject i's baseline value; Age_{ij} is the age at the MRI measurement for subject i at the time point j; Sex_{i} denotes the subject sex; ADI changes_{ij} is the change in ADI from baseline ADI for subject i at time point j; Baseline ADI_{i} is the baseline ADI value for subject i; $\text{Diagnosis}_{\text{i}}$ is the cognitive diagnosis either as cognitive normal (CN) or cognitively impaired (CI, includes MCI and AD) for subject i; b_{i} is the random intercept for subject i, and ϵ_{ij} is the residual error. Two-way interaction terms between diagnosis and ADI changes were included to examine how changes in ADI from baseline are associated with changes in MMSE/CDR-SB from baseline scores. Age_{ij} and ADI_{ij} values were standardized using the baseline mean and standard deviation. ADI $\text{changes}_{\text{ij}}$ were computed as the difference between the standardized ADI at time j and the subject's baseline ADI. Whole brain white matter $\text{ADI}_{\text{NODDI}}$, $\text{ADI}_{\text{C-NODDI}}$, and ADI_{SMI} values were used. Full statistical results are presented below.

	$\text{ADI}_{\text{NODDI}}$ changes associate with MMSE				
	Estimate	Std. Error	df	t value	Pr(>|t|)
(Intercept)	-1.79e-01	1.94e-01	2.57e+02	-9.25e-01	3.56e-01
Sex-Male	1.60e-01	2.01e-01	2.34e+02	7.97e-01	4.26e-01
Age	-1.28e-01	1.03e-01	2.62e+02	-1.24e+00	2.16e-01
Diagnosis-CN	1.55e-01	2.08e-01	2.50e+02	7.45e-01	4.57e-01
ADI changes	1.53e+00	3.77e-01	3.05e+02	4.07e+00	6.07e-05
ADI baseline	2.80e-02	1.34e-01	2.22e+02	2.08e-01	8.35e-01
Diagnosis-CN × ADI changes	-1.77e+00	6.37e-01	3.03e+02	-2.78e+00	5.77e-03
Diagnosis-CN × ADI baseline	-8.78e-02	1.98e-01	2.37e+02	-4.43e-01	6.58e-01

	ADI$_{C\text{-}NODDI}$ changes associate with MMSE				
	Estimate	Std. Error	df	t value	Pr(>|t|)
(Intercept)	-1.03e-01	1.98e-01	2.63e+02	-5.21e-01	6.03e-01
Sex-Male	1.80e-01	1.95e-01	2.33e+02	9.21e-01	3.58e-01
Age	-1.06e-01	1.14e-01	2.58e+02	-9.30e-01	3.53e-01
Diagnosis-CN	8.55e-02	2.20e-01	2.57e+02	3.88e-01	6.98e-01
ADI changes	1.25e+00	3.85e-01	2.97e+02	3.25e+00	1.27e-03
ADI baseline	2.45e-01	1.55e-01	2.33e+02	1.58e+00	1.15e-01
Diagnosis-CN × ADI changes	-1.75e+00	5.32e-01	3.01e+02	-3.29e+00	1.13e-03
Diagnosis-CN × ADI baseline	-3.40e-01	2.11e-01	2.38e+02	-1.61e+00	1.08e-01

	ADI$_{SMI}$ changes associate with MMSE				
	Estimate	Std. Error	df	t value	Pr(>|t|)
(Intercept)	-3.59e-01	1.88e-01	2.41e+02	-1.91e+00	5.68e-02
Sex-Male	1.46e-01	1.98e-01	2.34e+02	7.38e-01	4.61e-01
Age	-1.58e-01	1.01e-01	2.62e+02	-1.56e+00	1.21e-01
Diagnosis-CN	3.61e-01	2.02e-01	2.36e+02	1.79e+00	7.49e-02
ADI changes	4.88e-02	2.55e-01	2.99e+02	1.91e-01	8.48e-01
ADI baseline	5.30e-03	1.59e-01	2.25e+02	3.34e-02	9.73e-01
Diagnosis-CN × ADI changes	3.26e-01	3.69e-01	3.05e+02	8.83e-01	3.78e-01
Diagnosis-CN × ADI baseline	-8.89e-02	2.02e-01	2.37e+02	-4.40e-01	6.60e-01

	ADI_{NODDI} changes associate with CDR-SB				
	Estimate	Std. Error	df	t value	Pr(>|t|)
(Intercept)	2.40e-01	9.20e-02	2.44e+02	2.61e+00	9.64e-03
Sex-Male	-4.48e-02	9.46e-02	2.19e+02	-4.73e-01	6.36e-01
Age	6.80e-02	4.89e-02	2.54e+02	1.39e+00	1.66e-01
Diagnosis-CN	-2.12e-01	9.79e-02	2.38e+02	-2.17e+00	3.13e-02
ADI changes	-1.47e+00	1.78e-01	2.92e+02	-8.24e+00	5.91e-15
ADI baseline	3.28e-02	6.25e-02	1.97e+02	5.26e-01	6.00e-01
Diagnosis-CN × ADI changes	1.42e+00	3.10e-01	3.02e+02	4.57e+00	7.26e-06
Diagnosis-CN × ADI baseline	-2.92e-02	9.23e-02	2.23e+02	-3.16e-01	7.52e-01

	$ADI_{C\text{-}NODDI}$ changes associate with CDR-SB				
	Estimate	Std. Error	df	t value	Pr(>|t|)
(Intercept)	1.38e-01	9.39e-02	2.52e+02	1.47e+00	1.43e-01
Sex-Male	-5.97e-02	9.09e-02	2.17e+02	-6.57e-01	5.12e-01
Age	1.11e-02	5.39e-02	2.51e+02	2.06e-01	8.37e-01
Diagnosis-CN	-9.59e-02	1.04e-01	2.46e+02	-9.25e-01	3.56e-01
ADI changes	-1.42e+00	1.82e-01	3.00e+02	-7.79e+00	1.07e-13
ADI baseline	-2.12e-01	7.21e-02	2.10e+02	-2.94e+00	3.68e-03
Diagnosis-CN × ADI changes	1.42e+00	2.59e-01	3.02e+02	5.48e+00	9.05e-08
Diagnosis-CN × ADI baseline	2.03e-01	9.77e-02	2.24e+02	2.08e+00	3.87e-02

	ADI_{SMI} changes associate with CDR-SB				
	Estimate	Std. Error	df	t value	Pr(>|t|)
(Intercept)	3.70e-01	9.42e-02	2.23e+02	3.93e+00	1.14e-04
Sex-Male	-1.61e-02	9.88e-02	2.15e+02	-1.63e-01	8.71e-01
Age	8.12e-02	5.13e-02	2.56e+02	1.58e+00	1.15e-01
Diagnosis-CN	-3.55e-01	1.01e-01	2.18e+02	-3.52e+00	5.32e-04
ADI changes	6.87e-02	1.34e-01	3.02e+02	5.13e-01	6.09e-01
ADI baseline	-1.60e-01	7.75e-02	2.01e+02	-2.07e+00	3.98e-02
Diagnosis-CN × ADI changes	-6.80e-02	1.94e-01	2.99e+02	-3.50e-01	7.27e-01
Diagnosis-CN × ADI baseline	1.83e-01	1.01e-01	2.24e+02	1.81e+00	7.15e-02

Diagnosis is referenced to CI and Sex is referenced to Female. All p-values are not corrected for multiple comparisons.

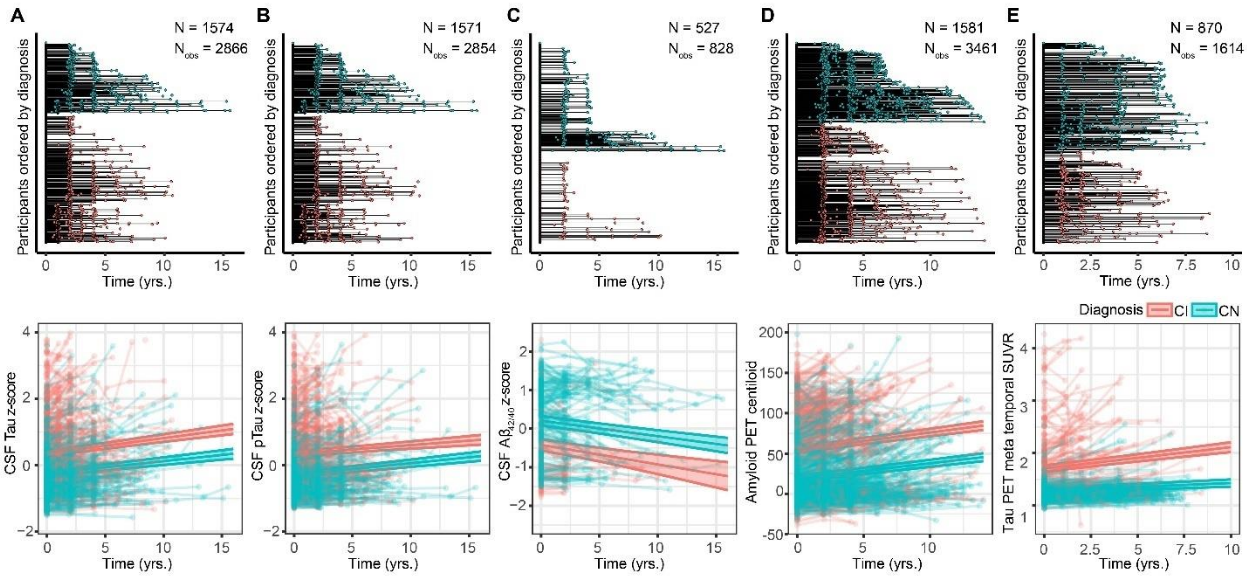

Figure S4. CSF and PET biomarkers longitudinal trajectories of the cognitively normal (CN) and cognitively impaired (CI) groups. Analyses included all available CSF and PET data available at the time of our analysis. Only tau-PET achieved significant group-level differentiation.

Table S4. Linear mixed-effects model results for Figure 5.

The model equation is specified as:

$$\mathrm{ADI/PET/CSF_{ij}} \sim \beta_0 + \beta_{\mathrm{Age}} \times \mathrm{Age_i} + \beta_{\mathrm{sex}} \times \mathrm{Sex_i} + \beta_{\mathrm{Time}} \times \mathrm{Time_{ij}} + \beta_{\mathrm{Diagnosis}} \times \mathrm{Diagnosis_i} + \beta_{\mathrm{Time \times Diagnosis}} \times \mathrm{Time_{ij}} \times \mathrm{Diagnosis_i} + \mathrm{b_i} + \epsilon_{\mathrm{ij}},$$

where $\mathrm{ADI/PET/CSF_{ij}}$ represents the longitudinal ADI, CSF or PET biomarkers value for subject i at time point j; $\mathrm{Age_i}$ is the age at the first MRI measurement, CSF or PET analysis for subject i; $\mathrm{Sex_i}$ denotes subject sex; $\mathrm{Time_{ij}}$ is the time since the first MRI measurement, CSF, or PET analysis for subject i at time point j, $\mathrm{Diagnosis_i}$ is the cognitive diagnosis either as cognitive normal (CN) or cognitively impaired (CI, which includes MCI and AD) for subject i; $\mathrm{Time_{ij}} \times \mathrm{Diagnosis_i}$ models differential time trajectories by diagnosis group. $\mathrm{b_i}$ is the random intercept for subject i and ϵ_{ij} is the residual error. $\mathrm{Age_i}$ was standardized using baseline mean and standard deviation, while $\mathrm{Time_{ij}}$ remained in original units (yrs.) for easier interpretation. Whole brain white matter $\mathrm{ADI_{C\text{-}NODDI}}$ values, CSF biomarkers (tau, $\mathrm{ptau_{181}}$, $\mathrm{A\beta_{42/40}}$), and PET imaging biomarkers (amyloid-PET, tau-PET) were used. Full statistical results are presented below.

	tau trajectory				
	Estimate	Std. Error	df	t value	Pr(>|t|)
(Intercept)	1.62e-01	1.60e-01	1.36e+02	1.01e+00	3.14e-01
Age	3.41e-01	8.56e-02	1.34e+02	3.98e+00	1.13e-04
Time	5.26e-02	2.10e-02	1.03e+02	2.51e+00	1.36e-02
Diagnosis-CN	-2.39e-01	1.78e-01	1.38e+02	-1.34e+00	1.82e-01
Sex-Male	-7.92e-02	1.78e-01	1.33e+02	-4.44e-01	6.57e-01
Time×Diagnosis-CN	9.56e-03	2.80e-02	1.03e+02	3.41e-01	7.34e-01

	$ptau_{181}$ trajectory				
	Estimate	Std. Error	df	t value	Pr(>|t|)
(Intercept)	1.50e-01	1.62e-01	1.39e+02	9.20e-01	3.59e-01
Age	3.47e-01	8.64e-02	1.36e+02	4.01e+00	1.00e-04
Time	3.93e-02	2.81e-02	1.06e+02	1.40e+00	1.65e-01
Diagnosis-CN	-2.54e-01	1.81e-01	1.42e+02	-1.41e+00	1.62e-01
Sex-Male	-3.03e-02	1.80e-01	1.33e+02	-1.69e-01	8.66e-01
Time×Diagnosis-CN	9.83e-03	3.76e-02	1.06e+02	2.61e-01	7.94e-01

	$A\beta_{42/40}$ trajectory				
	Estimate	Std. Error	df	t value	Pr(>|t|)
(Intercept)	-2.47e-01	1.63e-01	1.12e+02	-1.52e+00	1.31e-01
Age	-3.88e-01	8.52e-02	1.11e+02	-4.56e+00	1.34e-05
Time	-5.17e-02	2.65e-02	5.66e+01	-1.95e+00	5.63e-02
Diagnosis-CN	3.68e-01	1.78e-01	1.13e+02	2.07e+00	4.08e-02
Sex-Male	9.12e-02	1.79e-01	1.11e+02	5.10e-01	6.11e-01
Time×Diagnosis-CN	4.62e-02	3.08e-02	5.65e+01	1.50e+00	1.39e-01

	Amyloid-PET trajectory				
	Estimate	Std. Error	df	t value	Pr(>|t|)
(Intercept)	4.10e+01	5.13e+00	1.90e+02	7.99e+00	1.28e-13
Age	1.15e+01	2.67e+00	1.86e+02	4.32e+00	2.58e-05
Time	2.08e+00	4.77e-01	1.94e+02	4.35e+00	2.21e-05
Diagnosis-CN	-2.69e+01	5.55e+00	1.91e+02	-4.84e+00	2.63e-06
Sex-Male	-1.14e+00	5.38e+00	1.85e+02	-2.12e-01	8.32e-01
Time×Diagnosis-CN	-1.91e-01	5.95e-01	1.94e+02	-3.20e-01	7.49e-01

	Tau-PET trajectory				
	Estimate	Std. Error	df	t value	Pr(>|t|)
(Intercept)	1.70e+00	6.93e-02	1.78e+02	2.45e+01	5.18e-59
Age	5.41e-03	3.65e-02	1.77e+02	1.48e-01	8.82e-01
Time	4.72e-02	7.81e-03	1.49e+02	6.05e+00	1.14e-08
Diagnosis-CN	-4.14e-01	7.52e-02	1.80e+02	-5.51e+00	1.26e-07
Sex-Male	-1.17e-01	7.34e-02	1.76e+02	-1.59e+00	1.13e-01
Time×Diagnosis-CN	-2.84e-02	1.02e-02	1.49e+02	-2.78e+00	6.09e-03

	$ADI_{C\text{-}NODDI}$ trajectory matching tau cohort				
	Estimate	Std. Error	df	t value	Pr(>|t|)
(Intercept)	-4.59e-01	1.32e-01	1.42e+02	-3.47e+00	6.78e-04
Age	-4.33e-01	7.05e-02	1.38e+02	-6.14e+00	8.10e-09
Time	-2.41e-01	4.23e-02	1.07e+02	-5.69e+00	1.12e-07
Diagnosis-CN	7.58e-01	1.48e-01	1.46e+02	5.14e+00	8.86e-07
Sex-Male	6.22e-02	1.45e-01	1.33e+02	4.28e-01	6.69e-01
Time×Diagnosis-CN	2.35e-01	5.55e-02	1.07e+02	4.23e+00	4.93e-05

	$ADI_{C\text{-}NODDI}$ trajectory matching $ptau_{181}$ cohort				
	Estimate	Std. Error	df	t value	Pr(>|t|)
(Intercept)	-4.59e-01	1.32e-01	1.42e+02	-3.47e+00	6.78e-04
Age	-4.33e-01	7.05e-02	1.38e+02	-6.14e+00	8.10e-09
Time	-2.41e-01	4.23e-02	1.07e+02	-5.69e+00	1.12e-07
Diagnosis-CN	7.58e-01	1.48e-01	1.46e+02	5.14e+00	8.86e-07
Sex-Male	6.22e-02	1.45e-01	1.33e+02	4.28e-01	6.69e-01
Time×Diagnosis-CN	2.35e-01	5.55e-02	1.07e+02	4.23e+00	4.93e-05

	$ADI_{C\text{-}NODDI}$ trajectory matching $A\beta_{42/40}$ cohort				
	Estimate	Std. Error	df	t value	Pr(>|t|)
(Intercept)	-5.05e-01	1.53e-01	1.19e+02	-3.30e+00	1.29e-03
Age	-3.64e-01	7.94e-02	1.14e+02	-4.59e+00	1.14e-05
Time	-2.63e-01	4.49e-02	8.92e+01	-5.85e+00	8.00e-08
Diagnosis-CN	8.40e-01	1.68e-01	1.21e+02	5.00e+00	1.98e-06
Sex-Male	3.90e-02	1.66e-01	1.12e+02	2.35e-01	8.15e-01
Time×Diagnosis-CN	2.55e-01	5.93e-02	8.94e+01	4.30e+00	4.29e-05

	$ADI_{C\text{-}NODDI}$ trajectory matching amyloid-PET cohort				
	Estimate	Std. Error	df	t value	Pr(>|t|)
(Intercept)	-3.67e-01	1.16e-01	1.96e+02	-3.15e+00	1.89e-03
Age	-4.60e-01	6.08e-02	1.93e+02	-7.56e+00	1.61e-12
Time	-2.07e-01	3.31e-02	1.33e+02	-6.26e+00	5.00e-09
Diagnosis-CN	6.46e-01	1.26e-01	2.00e+02	5.10e+00	7.71e-07
Sex-Male	-5.80e-03	1.22e-01	1.87e+02	-4.76e-02	9.62e-01
Time×Diagnosis-CN	1.79e-01	4.40e-02	1.34e+02	4.07e+00	7.90e-05

	$ADI_{C\text{-}NODDI}$ trajectory matching tau-PET cohort				
	Estimate	Std. Error	df	t value	Pr(>|t|)
(Intercept)	-3.95e-01	1.16e-01	1.89e+02	-3.39e+00	8.39e-04
Age	-4.69e-01	6.11e-02	1.87e+02	-7.66e+00	9.45e-13
Time	-2.04e-01	3.27e-02	1.30e+02	-6.25e+00	5.36e-09
Diagnosis-CN	6.75e-01	1.27e-01	1.93e+02	5.32e+00	2.79e-07
Sex-Male	2.50e-02	1.22e-01	1.81e+02	2.05e-01	8.38e-01
Time×Diagnosis-CN	1.89e-01	4.42e-02	1.31e+02	4.29e+00	3.50e-05

Diagnosis is referenced to CI and Sex is referenced to Female. All p-values are not corrected for multiple comparisons.

Table S5. Linear mixed-effects model results for Figures 6, 7 and 8.

The model equation is specified as:

$$\mathrm{MMSE/CDR\text{-}SB}_{ij} \sim \beta_0 + \beta_{Age} \times \mathrm{Age}_i + \beta_{sex} \times \mathrm{Sex}_i + \beta_{Time} \times \mathrm{Time}_{ij} + \beta_{Diagnosis} \times \mathrm{Diagnosis}_i + \beta_{Biomarker} \times \mathrm{Biomarker}_i + \beta_{Time\times Biomaker} \times \mathrm{Time}_{ij} \times \mathrm{Biomarker}_i + \beta_{Time\times Diagnosis} \times \mathrm{Time}_{ij} \times \mathrm{Diagnosis}_i + \beta_{Biomarker\times Diagnosis} \times \mathrm{Biomarker}_i \times \mathrm{Diagnosis}_i + \beta_{Time\times Diagnosis\times Biomarker} \times \mathrm{Time}_{ij} \times \mathrm{Diagnosis}_i \times \mathrm{Biomarker}_i + b_i + \epsilon_{ij},$$

where $\mathrm{MMSE/CDR\text{-}SB}_{ij}$ represents the longitudinal MMSE or CDR-SB scores for subject i at time point j; Age_i is the age at the baseline CSF, PET or MRI measure for subject i; Sex_i is the sex for subject i; Time_{ij} is the time since the baseline CSF, PET or MRI measure for subject i at time point j; $\mathrm{Diagnosis}_i$ is the cognitive diagnosis either as cognitive normal (CN) or cognitively impaired (CI, which includes MCI and AD) for subject i. The model includes all two-way interactions and the three-way interaction among Time_{ij}, $\mathrm{Diagnosis}_i$, $\mathrm{Biomarker}_i$. b_i is the random intercept for subject i and ϵ_{ij} is the residual errors. Age_i and $\mathrm{Biomarker}_i$ were standardized using the baseline mean and standard deviation, while Time_{ij} was kept in its original units (yrs.) for easier interpretation. Biomarkers included whole brain white matter $\mathrm{ADI}_{\mathrm{C\text{-}NODDI}}$, CSF-$\mathrm{A}\beta_{42/40}$, amyloid-PET and tau-PET, the best performing biomarkers. Full statistical results are presented below.

Figure 6:

	$\mathrm{A}\beta_{42/40}$ predicts prospective MMSE				
	Estimate	Std. Error	df	t value	Pr(>|t|)
(Intercept)	2.58e+01	5.84e-01	1.04e+02	4.41e+01	2.22e-69
Age	-3.00e-01	3.21e-01	9.41e+01	-9.34e-01	3.53e-01
Sex-Male	8.40e-01	6.25e-01	9.46e+01	1.34e+00	1.82e-01
Diagnosis-CN	3.25e+00	6.49e-01	1.10e+02	5.01e+00	2.14e-06
Time	-2.32e-01	1.05e-01	1.87e+02	-2.22e+00	2.77e-02
Biomarker	2.05e+00	4.57e-01	1.07e+02	4.48e+00	1.88e-05
Diagnosis-CN × Time	1.66e-01	1.32e-01	1.86e+02	1.25e+00	2.13e-01
Diagnosis-CN × Biomarker	-2.34e+00	6.25e-01	1.09e+02	-3.74e+00	2.94e-04
Time × Biomarker	2.83e-01	9.45e-02	1.87e+02	3.00e+00	3.08e-03

Diagnosis-CN × Time × Biomarker	-2.22e-01	1.29e-01	1.85e+02	-1.73e+00	8.56e-02

	$ADI_{C\text{-}NODDI}$ predicts prospective MMSE				
	Estimate	Std. Error	df	t value	Pr(>|t|)
(Intercept)	2.61e+01	5.81e-01	1.21e+02	4.49e+01	3.37e-77
Age	-1.67e-01	3.06e-01	1.04e+02	-5.46e-01	5.86e-01
Sex-Male	1.38e+00	6.10e-01	1.03e+02	2.27e+00	2.55e-02
Diagnosis-CN	2.84e+00	6.76e-01	1.26e+02	4.21e+00	4.83e-05
Time	-3.17e-01	1.65e-01	1.50e+02	-1.91e+00	5.74e-02
Biomarker	2.02e+00	4.71e-01	1.23e+02	4.30e+00	3.50e-05
Diagnosis-CN × Time	2.95e-01	2.09e-01	1.47e+02	1.41e+00	1.59e-01
Diagnosis-CN × Biomarker	-2.45e+00	6.72e-01	1.24e+02	-3.64e+00	3.95e-04
Time × Biomarker	3.23e-01	1.65e-01	1.50e+02	1.96e+00	5.24e-02
Diagnosis-CN × Time × Biomarker	-4.06e-01	2.12e-01	1.46e+02	-1.91e+00	5.76e-02

	$A\beta_{42/40}$ predicts prospective CDR-SB				
	Estimate	Std. Error	df	t value	Pr(>|t|)
(Intercept)	2.43e+00	3.39e-01	1.05e+02	7.17e+00	1.06e-10
Age	2.39e-01	1.82e-01	9.08e+01	1.31e+00	1.93e-01
Sex-Male	-5.16e-01	3.52e-01	9.26e+01	-1.47e+00	1.46e-01
Diagnosis-CN	-2.25e+00	3.76e-01	1.11e+02	-6.00e+00	2.50e-08
Time	3.17e-01	6.37e-02	2.12e+02	4.98e+00	1.34e-06
Biomarker	-1.03e+00	2.73e-01	1.09e+02	-3.77e+00	2.63e-04
Diagnosis-CN × Time	-2.97e-01	8.00e-02	2.09e+02	-3.72e+00	2.60e-04
Diagnosis-CN × Biomarker	1.16e+00	3.59e-01	1.11e+02	3.22e+00	1.68e-03
Time × Biomarker	-2.81e-01	6.10e-02	2.12e+02	-4.61e+00	6.99e-06
Diagnosis-CN × Time × Biomarker	2.68e-01	7.91e-02	2.08e+02	3.39e+00	8.28e-04

	ADI$_{C\text{-}NODDI}$ predicts prospective CDR-SB				
	Estimate	Std. Error	df	t value	Pr(>|t|)
(Intercept)	2.38e+00	3.40e-01	1.26e+02	7.00e+00	1.36e-10
Age	1.97e-01	1.74e-01	1.03e+02	1.13e+00	2.60e-01
Sex-Male	-8.28e-01	3.41e-01	1.03e+02	-2.43e+00	1.70e-02
Diagnosis-CN	-2.19e+00	3.93e-01	1.32e+02	-5.58e+00	1.29e-07
Time	3.93e-01	1.06e-01	1.74e+02	3.71e+00	2.76e-04
Biomarker	-1.20e+00	2.80e-01	1.31e+02	-4.27e+00	3.76e-05
Diagnosis-CN × Time	-3.72e-01	1.29e-01	1.72e+02	-2.89e+00	4.39e-03
Diagnosis-CN × Biomarker	1.43e+00	3.85e-01	1.33e+02	3.71e+00	3.02e-04
Time × Biomarker	-3.13e-01	1.05e-01	1.73e+02	-2.97e+00	3.40e-03
Diagnosis-CN × Time × Biomarker	3.32e-01	1.31e-01	1.71e+02	2.53e+00	1.22e-02

Diagnosis is referenced to CI and Sex is referenced to Female. All p-values are not corrected for multiple comparisons.

Figure 7:

	amyloid-PET predicts prospective MMSE				
	Estimate	Std. Error	df	t value	Pr(>|t|)
(Intercept)	2.73e+01	3.30e-01	1.97e+02	8.27e+01	8.93e-155
Age	-4.82e-03	1.69e-01	1.81e+02	-2.86e-02	9.77e-01
Sex-Male	-3.98e-02	3.24e-01	1.70e+02	-1.23e-01	9.02e-01
Diagnosis-CN	1.91e+00	3.63e-01	2.17e+02	5.26e+00	3.43e-07
Time	-3.47e-01	6.97e-02	3.96e+02	-4.98e+00	9.67e-07
Biomarker	-1.10e+00	2.27e-01	2.09e+02	-4.85e+00	2.41e-06
Diagnosis-CN × Time	2.77e-01	9.33e-02	3.99e+02	2.97e+00	3.19e-03
Diagnosis-CN × Biomarker	1.08e+00	3.60e-01	2.23e+02	3.01e+00	2.90e-03
Time × Biomarker	-3.65e-01	5.76e-02	3.92e+02	-6.33e+00	6.54e-10
Diagnosis-CN × Time × Biomarker	3.25e-01	9.48e-02	3.96e+02	3.43e+00	6.70e-04

	$ADI_{C\text{-}NODDI}$ predicts prospective MMSE				
	Estimate	Std. Error	df	t value	Pr(>|t|)
(Intercept)	2.64e+01	4.29e-01	1.98e+02	6.16e+01	3.65e-131
Age	1.92e-02	2.40e-01	1.77e+02	8.02e-02	9.36e-01
Sex-Male	6.48e-01	4.20e-01	1.68e+02	1.54e+00	1.25e-01
Diagnosis-CN	2.49e+00	4.76e-01	2.11e+02	5.24e+00	3.89e-07
Time	-2.87e-01	1.46e-01	2.28e+02	-1.97e+00	5.03e-02
Biomarker	1.28e+00	3.54e-01	2.07e+02	3.63e+00	3.61e-04
Diagnosis-CN × Time	1.68e-01	1.76e-01	2.25e+02	9.54e-01	3.41e-01
Diagnosis-CN × Biomarker	-1.21e+00	4.72e-01	2.10e+02	-2.56e+00	1.12e-02
Time × Biomarker	3.49e-01	1.59e-01	2.32e+02	2.20e+00	2.89e-02
Diagnosis-CN × Time × Biomarker	-3.67e-01	1.89e-01	2.29e+02	-1.94e+00	5.38e-02

	amyloid-PET predicts prospective CDR-SB				
	Estimate	Std. Error	df	t value	Pr(>|t|)
(Intercept)	1.62e+00	2.21e-01	1.97e+02	7.34e+00	5.56e-12
Age	-8.44e-03	1.11e-01	1.79e+02	-7.62e-02	9.39e-01
Sex-Male	1.54e-01	2.17e-01	1.77e+02	7.10e-01	4.79e-01
Diagnosis-CN	-1.58e+00	2.41e-01	2.11e+02	-6.58e+00	3.67e-10
Time	2.44e-01	3.80e-02	4.24e+02	6.41e+00	3.77e-10
Biomarker	5.57e-01	1.52e-01	2.06e+02	3.67e+00	3.08e-04
Diagnosis-CN × Time	-2.39e-01	5.08e-02	4.25e+02	-4.70e+00	3.60e-06
Diagnosis-CN × Biomarker	-6.02e-01	2.35e-01	2.12e+02	-2.56e+00	1.12e-02
Time × Biomarker	2.49e-01	3.11e-02	4.21e+02	8.01e+00	1.13e-14
Diagnosis-CN × Time × Biomarker	-2.15e-01	4.99e-02	4.23e+02	-4.31e+00	2.00e-05

	ADI$_{C\text{-}NODDI}$ predicts prospective CDR-SB				
	Estimate	Std. Error	df	t value	Pr(>|t|)
(Intercept)	2.07e+00	2.55e-01	2.10e+02	8.14e+00	3.49e-14
Age	-5.45e-03	1.38e-01	1.78e+02	-3.96e-02	9.68e-01
Sex-Male	-4.89e-01	2.44e-01	1.76e+02	-2.00e+00	4.71e-02
Diagnosis-CN	-1.90e+00	2.81e-01	2.23e+02	-6.79e+00	1.03e-10
Time	2.86e-01	8.06e-02	2.77e+02	3.54e+00	4.65e-04
Biomarker	-1.06e+00	2.10e-01	2.22e+02	-5.06e+00	8.96e-07
Diagnosis-CN × Time	-2.42e-01	9.62e-02	2.75e+02	-2.52e+00	1.24e-02
Diagnosis-CN × Biomarker	1.11e+00	2.77e-01	2.25e+02	4.02e+00	7.93e-05
Time × Biomarker	-4.28e-01	8.99e-02	2.81e+02	-4.76e+00	3.13e-06
Diagnosis-CN × Time × Biomarker	4.29e-01	1.06e-01	2.78e+02	4.05e+00	6.60e-05

Diagnosis is referenced to CI and Sex is referenced to Female. All p-values are not corrected for multiple comparisons.

Figure 8:

	tau-PET predicts prospective MMSE				
	Estimate	Std. Error	df	t value	Pr(>|t|)
(Intercept)	2.71e+01	4.02e-01	1.96e+02	6.75e+01	8.53e-138
Age	-1.61e-01	1.98e-01	1.71e+02	-8.13e-01	4.18e-01
Sex-Male	-1.17e-02	3.88e-01	1.61e+02	-3.01e-02	9.76e-01
Diagnosis-CN	2.25e+00	4.45e-01	2.18e+02	5.04e+00	9.60e-07
Time	-4.30e-01	9.68e-02	2.72e+02	-4.45e+00	1.27e-05
Biomarker	-1.22e+00	2.70e-01	2.18e+02	-4.53e+00	9.55e-06
Diagnosis-CN × Time	2.62e-01	1.28e-01	2.74e+02	2.04e+00	4.23e-02
Diagnosis-CN × Biomarker	1.56e+00	4.48e-01	2.28e+02	3.48e+00	5.99e-04
Time × Biomarker	-3.19e-01	9.35e-02	2.82e+02	-3.42e+00	7.29e-04
Diagnosis-CN × Time × Biomarker	9.77e-02	1.45e-01	2.89e+02	6.73e-01	5.01e-01

	$ADI_{C\text{-}NODDI}$ predicts prospective MMSE				
	Estimate	Std. Error	df	t value	Pr(>|t|)
(Intercept)	2.68e+01	4.28e-01	1.97e+02	6.26e+01	8.45e-132
Age	-6.70e-03	2.24e-01	1.75e+02	-2.99e-02	9.76e-01
Sex-Male	4.60e-01	4.08e-01	1.63e+02	1.13e+00	2.61e-01
Diagnosis-CN	2.25e+00	4.83e-01	2.09e+02	4.65e+00	5.83e-06
Time	-3.38e-01	1.41e-01	2.32e+02	-2.40e+00	1.73e-02
Biomarker	1.18e+00	3.50e-01	2.07e+02	3.37e+00	8.88e-04
Diagnosis-CN × Time	2.12e-01	1.75e-01	2.29e+02	1.21e+00	2.28e-01
Diagnosis-CN × Biomarker	-1.16e+00	4.75e-01	2.08e+02	-2.45e+00	1.52e-02
Time × Biomarker	3.83e-01	1.56e-01	2.37e+02	2.46e+00	1.48e-02
Diagnosis-CN × Time × Biomarker	-4.02e-01	1.88e-01	2.33e+02	-2.13e+00	3.39e-02

	tau-PET predicts prospective CDR-SB				
	Estimate	Std. Error	df	t value	Pr(>|t|)
(Intercept)	1.91e+00	2.58e-01	2.02e+02	7.38e+00	3.93e-12
Age	3.08e-02	1.28e-01	1.77e+02	2.42e-01	8.09e-01
Sex-Male	-1.47e-03	2.49e-01	1.69e+02	-5.91e-03	9.95e-01
Diagnosis-CN	-1.87e+00	2.84e-01	2.21e+02	-6.57e+00	3.46e-10
Time	3.69e-01	5.38e-02	3.07e+02	6.86e+00	3.83e-11
Biomarker	4.83e-01	1.72e-01	2.21e+02	2.80e+00	5.52e-03
Diagnosis-CN × Time	-3.56e-01	7.16e-02	3.09e+02	-4.96e+00	1.14e-06
Diagnosis-CN × Biomarker	-4.98e-01	2.81e-01	2.29e+02	-1.77e+00	7.76e-02
Time × Biomarker	3.50e-01	5.32e-02	3.18e+02	6.58e+00	1.89e-10
Diagnosis-CN × Time × Biomarker	-3.48e-01	8.19e-02	3.19e+02	-4.24e+00	2.89e-05

	$ADI_{C\text{-}NODDI}$ predicts prospective CDR-SB				
	Estimate	Std. Error	df	t value	Pr(>|t|)
(Intercept)	2.01e+00	2.62e-01	2.10e+02	7.66e+00	6.79e-13
Age	-2.05e-02	1.36e-01	1.76e+02	-1.51e-01	8.80e-01
Sex-Male	-3.58e-01	2.46e-01	1.71e+02	-1.46e+00	1.47e-01
Diagnosis-CN	-1.90e+00	2.93e-01	2.20e+02	-6.49e+00	5.67e-10
Time	3.03e-01	8.19e-02	2.73e+02	3.70e+00	2.62e-04
Biomarker	-8.54e-01	2.15e-01	2.21e+02	-3.98e+00	9.49e-05
Diagnosis-CN × Time	-2.70e-01	9.89e-02	2.70e+02	-2.73e+00	6.70e-03
Diagnosis-CN × Biomarker	8.97e-01	2.87e-01	2.21e+02	3.12e+00	2.04e-03
Time × Biomarker	-4.50e-01	9.11e-02	2.77e+02	-4.94e+00	1.35e-06
Diagnosis-CN × Time × Biomarker	4.55e-01	1.08e-01	2.73e+02	4.20e+00	3.59e-05

Diagnosis is referenced to CI and Sex is referenced to Female. All p-values are not corrected for multiple comparisons.

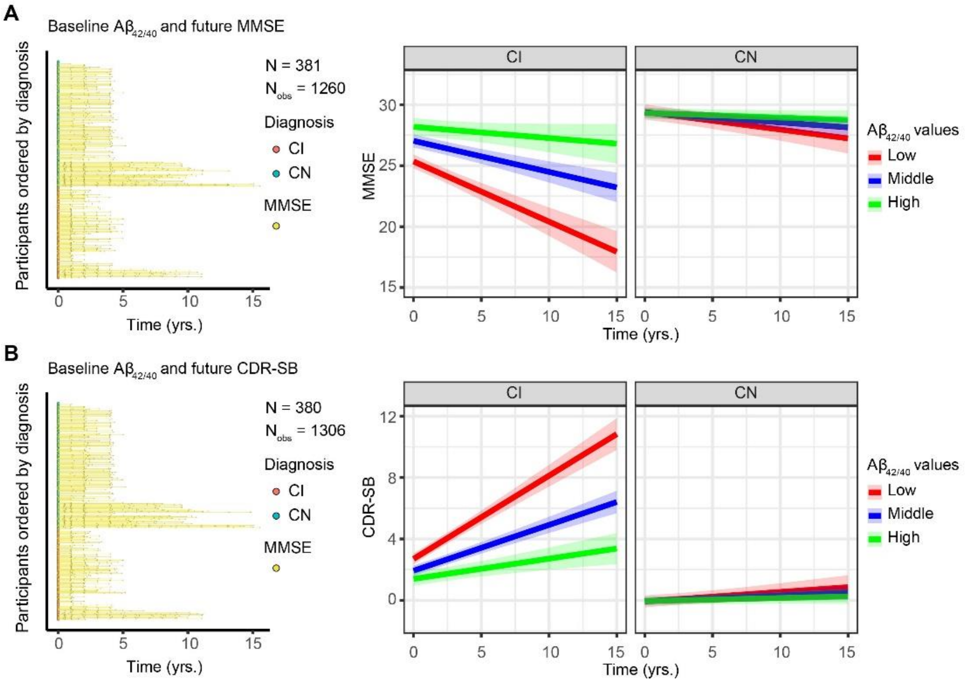

Figure S5. Baseline CSF-A$\beta_{42/40}$ measurements predict prospective changes in cognition and function, as measured using the Mini-Mental State Examination (MMSE) and Clinical Dementia Rating-Sum of Boxes (CDR-SB) scores. Analyses included all available CSF-A$\beta_{42/40}$ available at the time of our analysis. The longitudinal distributions of MMSE and CDR-SB anchored at the first CSF-A$\beta_{42/40}$ measurement of each subject, with cognitively normal (CN) and cognitively impaired (CI) subjects color-coded.

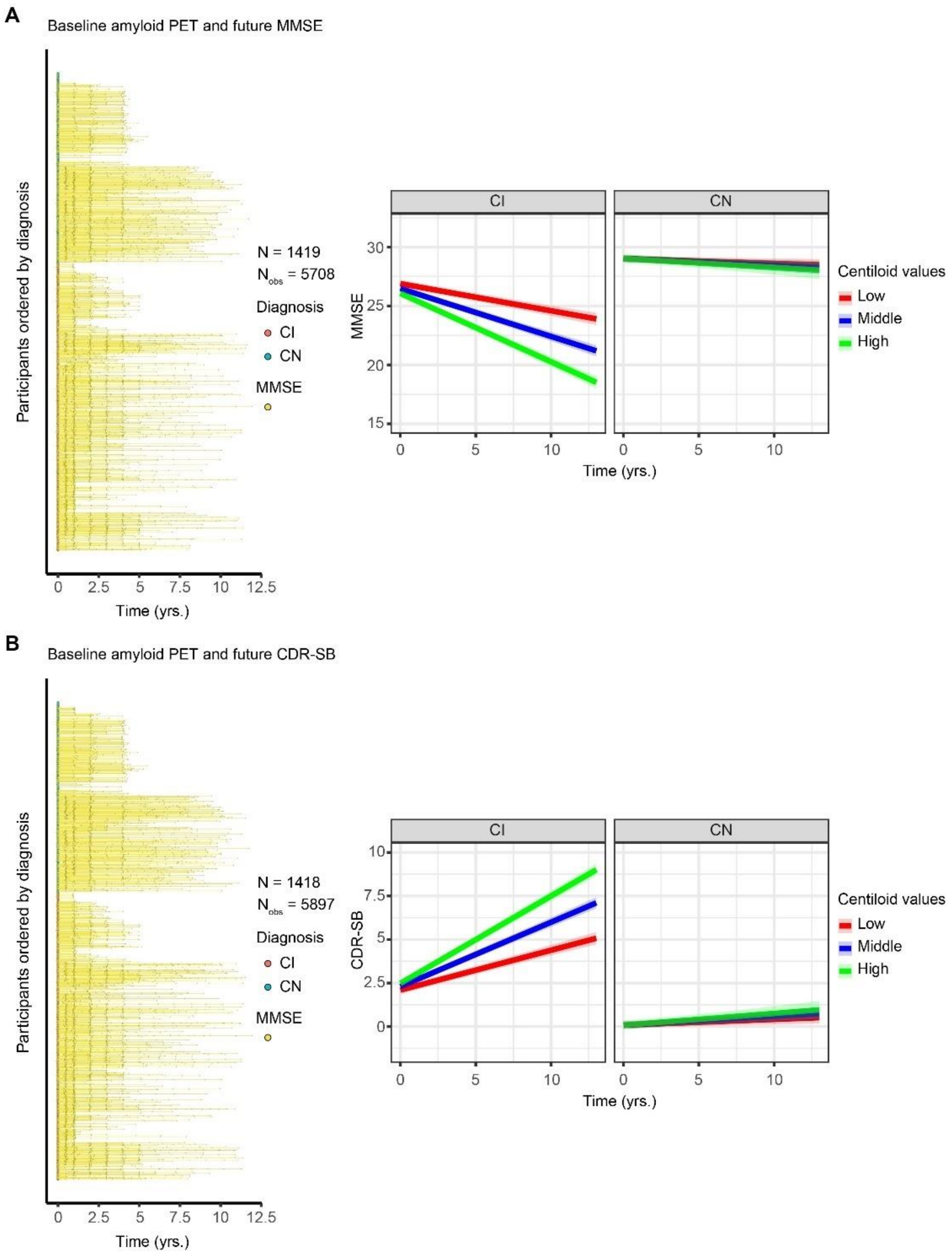

Figure S6. Baseline amyloid-PET measurements predict prospective changes in cognition and function, as measured using the Mini-Mental State Examination (MMSE) and Clinical Dementia Rating-Sum of Boxes (CDR-SB) scores. Analyses included all available amyloid-PET available at the time of our analysis. The longitudinal distributions of MMSE and CDR-SB anchored at the first amyloid-PET measurement of each subject, with cognitively normal (CN) and cognitively impaired (CI) subjects color-coded.

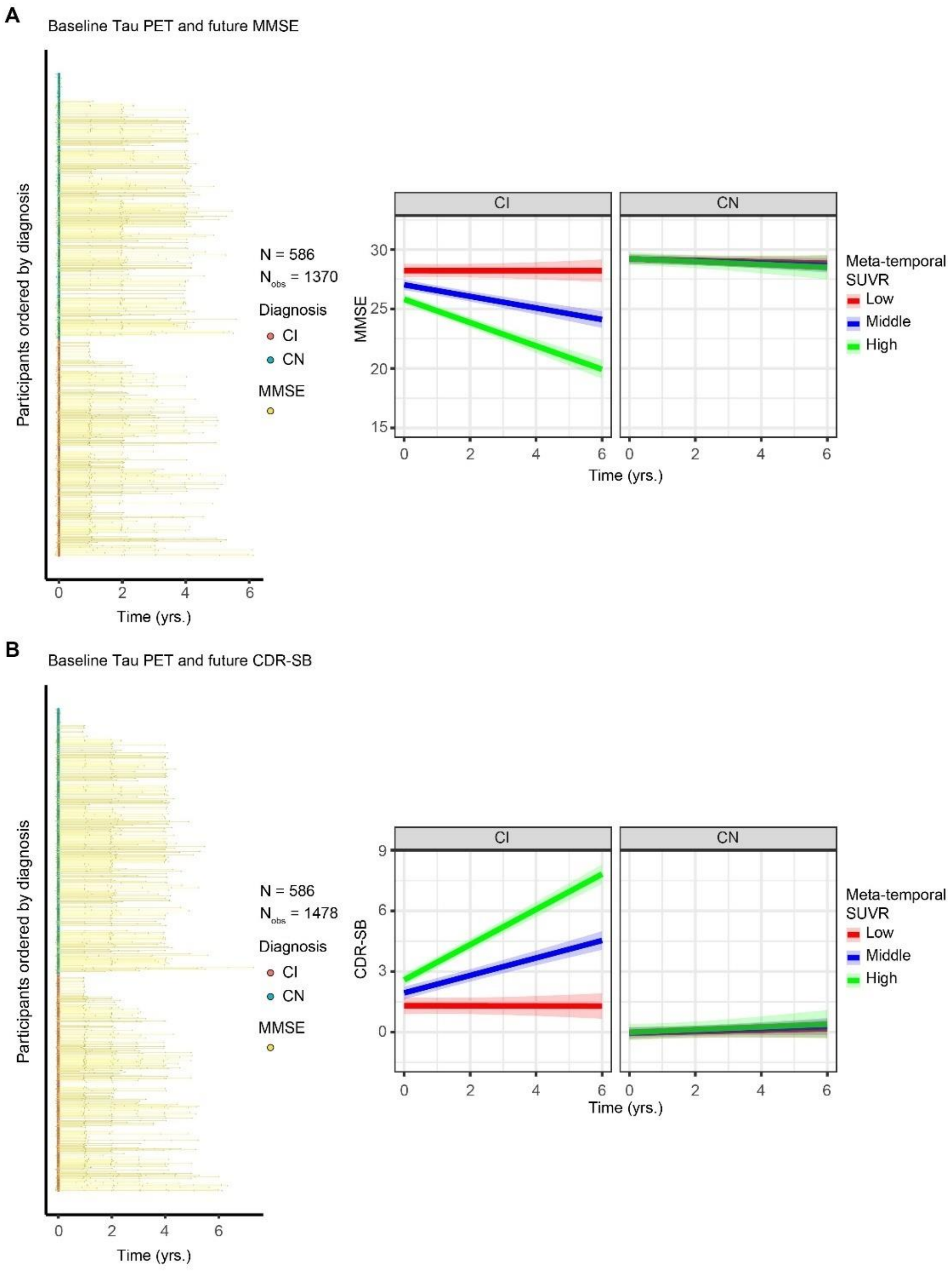

Figure S7. Baseline tau-PET measurements predict prospective changes in cognition and function, as measured using the Mini-Mental State Examination (MMSE) and Clinical Dementia Rating-Sum of Boxes (CDR-SB) scores. Analyses included all available tau-PET available at the time of our analysis. The longitudinal distributions of MMSE and CDR-SB anchored at the first tau-PET measurement of each subject, with cognitively normal (CN) and cognitively impaired (CI) subjects color-coded.

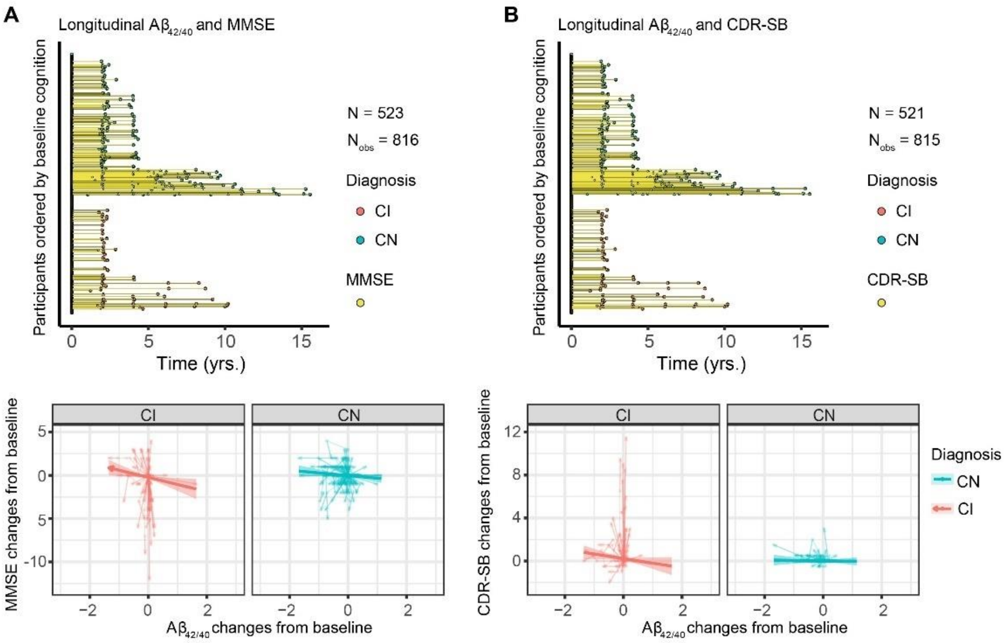

Figure S8. Associations between changes from baseline in CSF-A$\beta_{42/40}$ and changes from baseline in MMSE (A) or CDR-SB (B). The top row shows the longitudinal distribution of MMSE or CDR-SB scores aligned with the nearest CSF-A$\beta_{42/40}$ measurements for linear mixed-effects modeling. Resuls were derived from all availailabe CSF-A$\beta_{42/40}$ measurements at the time of analysis.

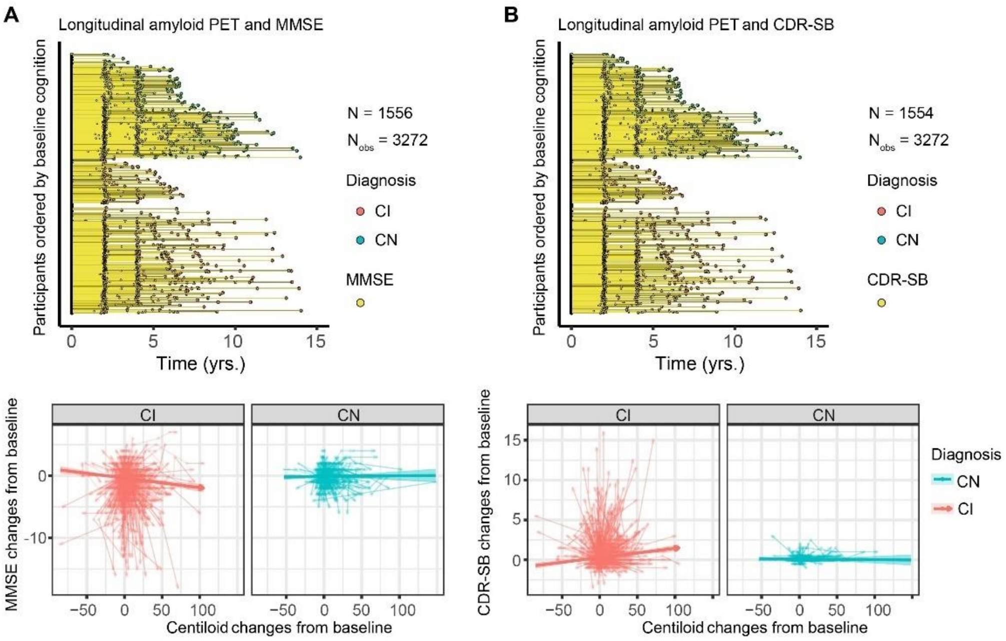

Figure S9. Associations between changes from baseline in amyloid-PET and changes from baseline in MMSE (A) or CDR-SB (B). The top row shows the longitudinal distribution of MMSE or CDR-SB scores aligned with the nearest amyloid-PET measurements for linear mixed-effects modeling. Resuls were derived from all availailabe amyloid-PET measurements at the time of analysis.

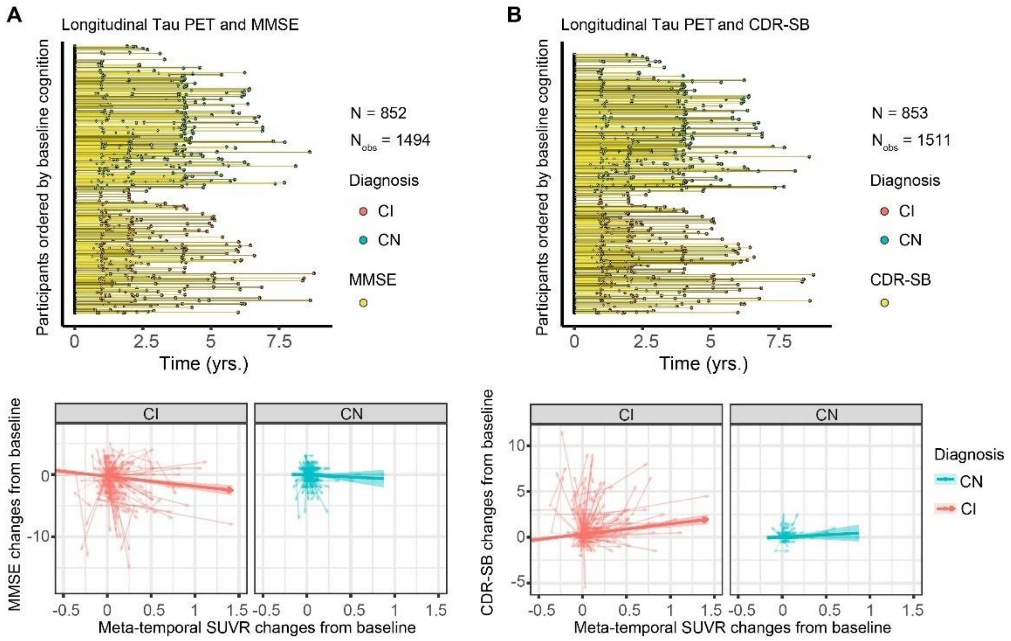

Figure S10. Associations between changes from baseline in tau-PET and changes from baseline in MMSE (A) or CDR-SB (B). The top row shows the longitudinal distribution of MMSE or CDR-SB scores aligned with the nearest tau-PET measurements for linear mixed-effects modeling. Resuls were derived from all availailabe tau-PET measurements at the time of analysis.

Table S6. Linear mixed-effects model results for Figures 9, 10 and 11.

The model equation is specified as:

$$\text{MMSE/CDR-SB changes}_{ij} \sim \beta_0 + \beta_{\text{sex}} \times \text{Sex}_i + \beta_{\text{Age}} \times \text{Age}_{ij} + \beta_{\text{Diagnosis}} \times \text{Diagnosis}_i + \beta_{\text{Biomarker changes}} \times \text{Biomaker changes}_{ij} + \beta_{\text{Diagnosis}\times\text{Biomarker changes}} \times \text{Diagnosis}_i \times \text{Biomarker changes}_{ij} + \beta_{\text{Baseline Biomarker}} \times \text{Baseline Biomarker}_i + \beta_{\text{Diagnosis}\times\text{Baseline Biomarker}} \times \text{Diagnosis}_i \times \text{Baseline Biomarker}_i + b_i + \epsilon_{ij},$$

where MMSE/CDR-SB_{ij} represents the longitudinal MMSE or CDR-SB changes for subject i at time point j from subject i's baseline value; Age_{ij} is the age at the CSF, PET, or MRI measurement for subject i at the time point j; Sex_i is the sex for subject i; $\text{Biomarker changes}_{ij}$ is the difference of Biomarker from its baseline Biomarker for subject i at time point j; $\text{Baseline Biomarker}_i$ is the subject's baseline biomarker value; Diagnosis_i is the cognitive diagnosis either as cognitive normal (CN) or cognitively impaired (CI, which includes MCI and AD) for subject i. b_i is the random intercept for subject i and ϵ_{ij} is the residual error. Two-way interaction terms between diagnosis and biomarker changes were included to assess how biomarker changes relate to cognitive changes. Age_{ij} and Biomarker_{ij} values were standardized using baseline mean and standard deviation, while $\text{Biomarker changes}_{ij}$ were calculated as the difference between the standardized biomarker value at time j and the subject's baseline biomarker value. Biomarkers included whole brain white matter $\text{ADI}_{\text{C-NODDI}}$, CSF-$\text{A}\beta_{42/40}$, amyloid-PET and tau-PET, the best performing biomarkers. Full statistical results are presented below. Full statistical results are presented below.

Figure 9:

	$A\beta_{42/40}$ changes associate with MMSE changes				
	Estimate	Std. Error	df	t value	Pr(>|t|)
(Intercept)	-5.89e-03	1.72e-01	1.64e+02	-3.42e-02	9.73e-01
Sex-Male	-3.02e-01	1.76e-01	1.64e+02	-1.72e+00	8.78e-02
Age	-1.78e-01	8.84e-02	1.64e+02	-2.02e+00	4.53e-02
Diagnosis-CN	1.53e-01	1.84e-01	1.64e+02	8.33e-01	4.06e-01
Biomarker changes	-3.58e+00	9.29e-01	1.64e+02	-3.85e+00	1.68e-04
Biomarker baseline	1.09e-01	1.41e-01	1.64e+02	7.78e-01	4.38e-01
Diagnosis-CN × Biomarker changes	3.31e+00	1.12e+00	1.64e+02	2.95e+00	3.67e-03
Diagnosis-CN × Biomarker baseline	-1.56e-01	1.75e-01	1.64e+02	-8.88e-01	3.76e-01

	$A\beta_{42/40}$ changes associate with CDR-SB changes				
	Estimate	Std. Error	df	t value	Pr(>|t|)
(Intercept)	2.66e-01	1.64e-01	1.58e+02	1.63e+00	1.06e-01
Sex-Male	9.74e-02	1.63e-01	1.58e+02	5.99e-01	5.50e-01
Age	1.27e-01	8.23e-02	1.58e+02	1.55e+00	1.24e-01
Diagnosis-CN	-2.97e-01	1.75e-01	1.58e+02	-1.70e+00	9.11e-02
Biomarker changes	2.57e-01	8.57e-01	1.58e+02	3.00e-01	7.65e-01
Biomarker baseline	-1.56e-01	1.36e-01	1.58e+02	-1.14e+00	2.55e-01
Diagnosis-CN × Biomarker changes	-2.02e-01	1.05e+00	1.58e+02	-1.93e-01	8.47e-01
Diagnosis-CN × Biomarker baseline	1.74e-01	1.66e-01	1.58e+02	1.05e+00	2.94e-01

	$ADI_{C\text{-}NODDI}$ changes associate with MMSE changes				
	Estimate	Std. Error	df	t value	Pr(>|t|)
(Intercept)	-8.32e-02	2.75e-01	1.65e+02	-3.03e-01	7.62e-01
Sex-Male	2.89e-01	2.70e-01	1.36e+02	1.07e+00	2.87e-01
Age	-7.59e-02	1.37e-01	1.53e+02	-5.56e-01	5.79e-01
Diagnosis-CN	1.31e-01	3.12e-01	1.61e+02	4.21e-01	6.74e-01
Biomarker changes	6.09e-01	4.10e-01	1.81e+02	1.48e+00	1.40e-01
Biomarker baseline	4.13e-01	2.04e-01	1.39e+02	2.02e+00	4.48e-02
Diagnosis-CN × Biomarker changes	-1.42e+00	6.09e-01	1.85e+02	-2.33e+00	2.08e-02
Diagnosis-CN × Biomarker baseline	-5.59e-01	2.84e-01	1.36e+02	-1.97e+00	5.12e-02

	$ADI_{C\text{-}NODDI}$ changes associate with CDR-SB changes				
	Estimate	Std. Error	df	t value	Pr(>|t|)
(Intercept)	2.66e-01	1.43e-01	1.56e+02	1.86e+00	6.52e-02
Sex-Male	-2.07e-01	1.35e-01	1.24e+02	-1.53e+00	1.29e-01
Age	6.86e-02	6.99e-02	1.43e+02	9.81e-01	3.28e-01
Diagnosis-CN	-1.96e-01	1.61e-01	1.52e+02	-1.22e+00	2.25e-01
Biomarker changes	-1.03e+00	2.12e-01	1.79e+02	-4.84e+00	2.74e-06
Biomarker baseline	-2.70e-01	1.04e-01	1.19e+02	-2.59e+00	1.08e-02
Diagnosis-CN × Biomarker changes	1.08e+00	3.30e-01	1.81e+02	3.28e+00	1.24e-03
Diagnosis-CN × Biomarker baseline	3.00e-01	1.43e-01	1.23e+02	2.10e+00	3.80e-02

Diagnosis is referenced to CI and Sex is referenced to Female. All p-values are not corrected for multiple comparisons.

Figure 10:

	amyloid-PET changes associate with MMSE changes				
	Estimate	Std. Error	df	t value	Pr(>|t|)
(Intercept)	5.19e-02	2.45e-01	1.89e+02	2.12e-01	8.32e-01
Sex-Male	1.75e-01	2.05e-01	1.81e+02	8.55e-01	3.94e-01
Age	-9.84e-02	1.02e-01	2.09e+02	-9.68e-01	3.34e-01
Diagnosis-CN	-8.02e-02	2.57e-01	1.91e+02	-3.13e-01	7.55e-01
Biomarker changes	-7.85e-02	1.34e-02	3.03e+02	-5.86e+00	1.21e-08
Biomarker baseline	-1.17e-02	3.24e-03	1.83e+02	-3.61e+00	4.01e-04
Diagnosis-CN × Biomarker changes	7.29e-02	2.10e-02	3.04e+02	3.47e+00	5.86e-04
Diagnosis-CN × Biomarker baseline	1.44e-02	6.29e-03	1.85e+02	2.29e+00	2.30e-02

	amyloid-PET changes associate with CDR-SB changes				
	Estimate	Std. Error	df	t value	Pr(>|t|)
(Intercept)	-3.67e-01	1.31e-01	1.67e+02	-2.80e+00	5.67e-03
Sex-Male	9.38e-02	1.07e-01	1.56e+02	8.80e-01	3.80e-01
Age	2.54e-02	5.18e-02	1.74e+02	4.91e-01	6.24e-01
Diagnosis-CN	3.75e-01	1.36e-01	1.67e+02	2.77e+00	6.29e-03
Biomarker changes	5.27e-02	7.13e-03	2.90e+02	7.39e+00	1.59e-12
Biomarker baseline	9.61e-03	1.70e-03	1.54e+02	5.66e+00	7.26e-08
Diagnosis-CN × Biomarker changes	-5.26e-02	1.17e-02	2.95e+02	-4.51e+00	9.56e-06
Diagnosis-CN × Biomarker baseline	-8.82e-03	3.24e-03	1.57e+02	-2.72e+00	7.26e-03

	ADI$_{C\text{-}NODDI}$ changes associate with MMSE changes				
	Estimate	Std. Error	df	t value	Pr(>|t|)
(Intercept)	-1.94e-01	2.29e-01	2.24e+02	-8.46e-01	3.98e-01
Sex-Male	3.87e-01	2.25e-01	1.97e+02	1.72e+00	8.63e-02
Age	-1.87e-01	1.28e-01	2.20e+02	-1.46e+00	1.47e-01
Diagnosis-CN	1.51e-01	2.52e-01	2.20e+02	5.98e-01	5.51e-01
Biomarker changes	1.15e+00	3.97e-01	2.53e+02	2.89e+00	4.13e-03
Biomarker baseline	3.80e-01	1.69e-01	1.99e+02	2.25e+00	2.56e-02
Diagnosis-CN × Biomarker changes	-1.67e+00	6.03e-01	2.55e+02	-2.76e+00	6.16e-03
Diagnosis-CN × Biomarker baseline	-5.51e-01	2.43e-01	2.08e+02	-2.26e+00	2.48e-02

	ADI$_{C\text{-}NODDI}$ changes associate with CDR-SB changes				
	Estimate	Std. Error	df	t value	Pr(>|t|)
(Intercept)	2.29e-01	1.08e-01	2.14e+02	2.11e+00	3.62e-02
Sex-Male	-1.10e-01	1.04e-01	1.82e+02	-1.06e+00	2.92e-01
Age	4.71e-02	6.08e-02	2.14e+02	7.75e-01	4.39e-01
Diagnosis-CN	-1.78e-01	1.19e-01	2.11e+02	-1.50e+00	1.35e-01
Biomarker changes	-1.37e+00	1.87e-01	2.54e+02	-7.34e+00	2.88e-12
Biomarker baseline	-2.24e-01	7.85e-02	1.78e+02	-2.85e+00	4.94e-03
Diagnosis-CN × Biomarker changes	1.29e+00	2.99e-01	2.54e+02	4.33e+00	2.16e-05
Diagnosis-CN × Biomarker baseline	2.57e-01	1.13e-01	1.97e+02	2.27e+00	2.40e-02

Diagnosis is referenced to CI and Sex is referenced to Female. All p-values are not corrected for multiple comparisons.

Figure 11:

	tau-PET changes associate with MMSE changes				
	Estimate	Std. Error	df	t value	Pr(>|t|)
(Intercept)	4.00e-01	4.81e-01	1.93e+02	8.32e-01	4.06e-01
Sex-Male	3.54e-01	2.38e-01	1.79e+02	1.49e+00	1.38e-01
Age	-3.68e-01	1.21e-01	2.08e+02	-3.05e+00	2.55e-03
Diagnosis-CN	-4.53e-01	1.70e+00	1.83e+02	-2.66e-01	7.91e-01
Biomarker changes	-1.01e+00	9.90e-01	2.88e+02	-1.02e+00	3.07e-01
Biomarker baseline	-7.97e-01	2.56e-01	2.10e+02	-3.11e+00	2.10e-03
Diagnosis-CN × Biomarker changes	2.42e+00	2.68e+00	2.87e+02	9.00e-01	3.69e-01
Diagnosis-CN × Biomarker baseline	6.92e-01	1.34e+00	1.78e+02	5.15e-01	6.07e-01

	tau-PET changes associate with CDR-SB changes				
	Estimate	Std. Error	df	t value	Pr(>|t|)
(Intercept)	-5.46e-03	3.10e-01	1.85e+02	-1.76e-02	9.86e-01
Sex-Male	-1.81e-01	1.54e-01	1.71e+02	-1.18e+00	2.41e-01
Age	2.17e-01	7.72e-02	1.96e+02	2.81e+00	5.47e-03
Diagnosis-CN	1.25e+00	1.10e+00	1.76e+02	1.13e+00	2.59e-01
Biomarker changes	7.18e-01	6.33e-01	2.88e+02	1.13e+00	2.58e-01
Biomarker baseline	3.79e-01	1.65e-01	2.04e+02	2.30e+00	2.24e-02
Diagnosis-CN × Biomarker changes	2.29e-02	1.71e+00	2.88e+02	1.34e-02	9.89e-01
Diagnosis-CN × Biomarker baseline	-1.33e+00	8.68e-01	1.71e+02	-1.53e+00	1.28e-01

	$ADI_{C\text{-}NODDI}$ changes associate with MMSE changes				
	Estimate	Std. Error	df	t value	Pr(>|t|)
(Intercept)	-1.17e-01	2.27e-01	2.21e+02	-5.15e-01	6.07e-01
Sex-Male	2.71e-01	2.26e-01	1.93e+02	1.20e+00	2.32e-01
Age	-1.13e-01	1.30e-01	2.20e+02	-8.72e-01	3.84e-01
Diagnosis-CN	7.06e-02	2.57e-01	2.14e+02	2.74e-01	7.84e-01
Biomarker changes	1.25e+00	4.11e-01	2.56e+02	3.04e+00	2.63e-03
Biomarker baseline	2.76e-01	1.73e-01	1.95e+02	1.60e+00	1.12e-01
Diagnosis-CN × Biomarker changes	-1.86e+00	5.82e-01	2.61e+02	-3.20e+00	1.55e-03
Diagnosis-CN × Biomarker baseline	-3.65e-01	2.44e-01	1.96e+02	-1.49e+00	1.37e-01

	$ADI_{C\text{-}NODDI}$ changes associate with CDR-SB changes				
	Estimate	Std. Error	df	t value	Pr(>|t|)
(Intercept)	1.75e-01	1.04e-01	2.11e+02	1.69e+00	9.31e-02
Sex-Male	-5.12e-02	1.03e-01	1.77e+02	-4.97e-01	6.20e-01
Age	-3.32e-03	6.03e-02	2.11e+02	-5.51e-02	9.56e-01
Diagnosis-CN	-1.33e-01	1.17e-01	2.03e+02	-1.13e+00	2.58e-01
Biomarker changes	-1.41e+00	1.89e-01	2.60e+02	-7.49e+00	1.09e-12
Biomarker baseline	-2.03e-01	7.70e-02	1.77e+02	-2.64e+00	9.14e-03
Diagnosis-CN × Biomarker changes	1.44e+00	2.78e-01	2.62e+02	5.19e+00	4.29e-07
Diagnosis-CN × Biomarker baseline	1.85e-01	1.11e-01	1.85e+02	1.66e+00	9.90e-02

Diagnosis is referenced to CI and Sex is referenced to Female. All p-values are not corrected for multiple comparisons.

Table S7. Linear mixed-effects model results for Figure 2 without AD subjects.

The model equation is specified as:

$$ADI_{ij} \sim \beta_0 + \beta_{Age} \times Age_i + \beta_{sex} \times Sex_i + \beta_{Time} \times Time_{ij} + \beta_{Diagnosis} \times Diagnosis_i + \beta_{Time \times Diagnosis} \times Time_{ij} \times Diagnosis_i + b_i + \epsilon_{ij},$$

where ADI_{ij} represents the longitudinal ADI value for subject i at time point j; Age_i is the age at the first MRI measurement for subject i; Sex_i denotes subject sex; $Time_{ij}$ is the time since the first MRI measurement for subject i at time point j; $Diagnosis_i$ indicates the cognitive diagnosis either as cognitive normal (CN) or cognitively impaired (CI, which only includes MCI); $Time_{ij} \times Diagnosis_i$ is the interaction term representing differential time trajectories of ADI for two diagnosis groups; b_i is the random intercept for subject i; ϵ_{ij} is the residual error. Age_i was standarized using baseline mean and standard deviation, while $Time_{ij}$ is in the original unit (yrs.) for easier interpreation. Whole brain white matter ADI_{NODDI}, $ADI_{C\text{-}NODDI}$, and ADI_{SMI} values were used. Full statistical results are presented below.

	ADI_{NODDI} trajectory				
	Estimate	Std. Error	df	t value	Pr(>|t|)
(Intercept)	-3.84e-01	1.33e-01	1.92e+02	-2.89e+00	4.26e-03
Age	-2.83e-01	6.79e-02	1.90e+02	-4.17e+00	4.67e-05
Time	-1.69e-01	3.80e-02	1.00e+02	-4.45e+00	2.23e-05
Diagnosis-CN	3.34e-01	1.41e-01	1.93e+02	2.36e+00	1.92e-02
Sex-Male	4.24e-01	1.38e-01	1.87e+02	3.07e+00	2.47e-03
Diagnosis-CN × Time	1.06e-01	4.56e-02	1.00e+02	2.34e+00	2.15e-02

	$ADI_{C-NODDI}$ trajectory				
	Estimate	Std. Error	df	t value	Pr(>|t|)
(Intercept)	-3.42e-01	1.19e-01	1.99e+02	-2.87e+00	4.57e-03
Age	-5.08e-01	6.09e-02	1.95e+02	-8.33e+00	1.37e-14
Time	-1.62e-01	4.55e-02	1.11e+02	-3.55e+00	5.59e-04
Diagnosis-CN	5.38e-01	1.27e-01	2.02e+02	4.23e+00	3.60e-05
Sex-Male	3.20e-02	1.23e-01	1.89e+02	2.59e-01	7.96e-01
Diagnosis-CN × Time	1.33e-01	5.45e-02	1.11e+02	2.43e+00	1.65e-02

	ADI_{SMI} trajectory				
	Estimate	Std. Error	df	t value	Pr(>|t|)
(Intercept)	-1.89e-01	1.44e-01	2.07e+02	-1.31e+00	1.92e-01
Age	-1.61e-01	7.33e-02	2.00e+02	-2.19e+00	2.94e-02
Time	9.29e-02	6.99e-02	1.26e+02	1.33e+00	1.86e-01
Diagnosis-CN	2.38e-01	1.54e-01	2.13e+02	1.54e+00	1.24e-01
Sex-Male	4.02e-02	1.48e-01	1.89e+02	2.73e-01	7.85e-01
Diagnosis-CN × Time	-5.80e-02	8.37e-02	1.26e+02	-6.93e-01	4.90e-01

Diagnosis was referenced to the MCI group, and sex was referenced to females. P-values were not corrected for multiple comparisons.

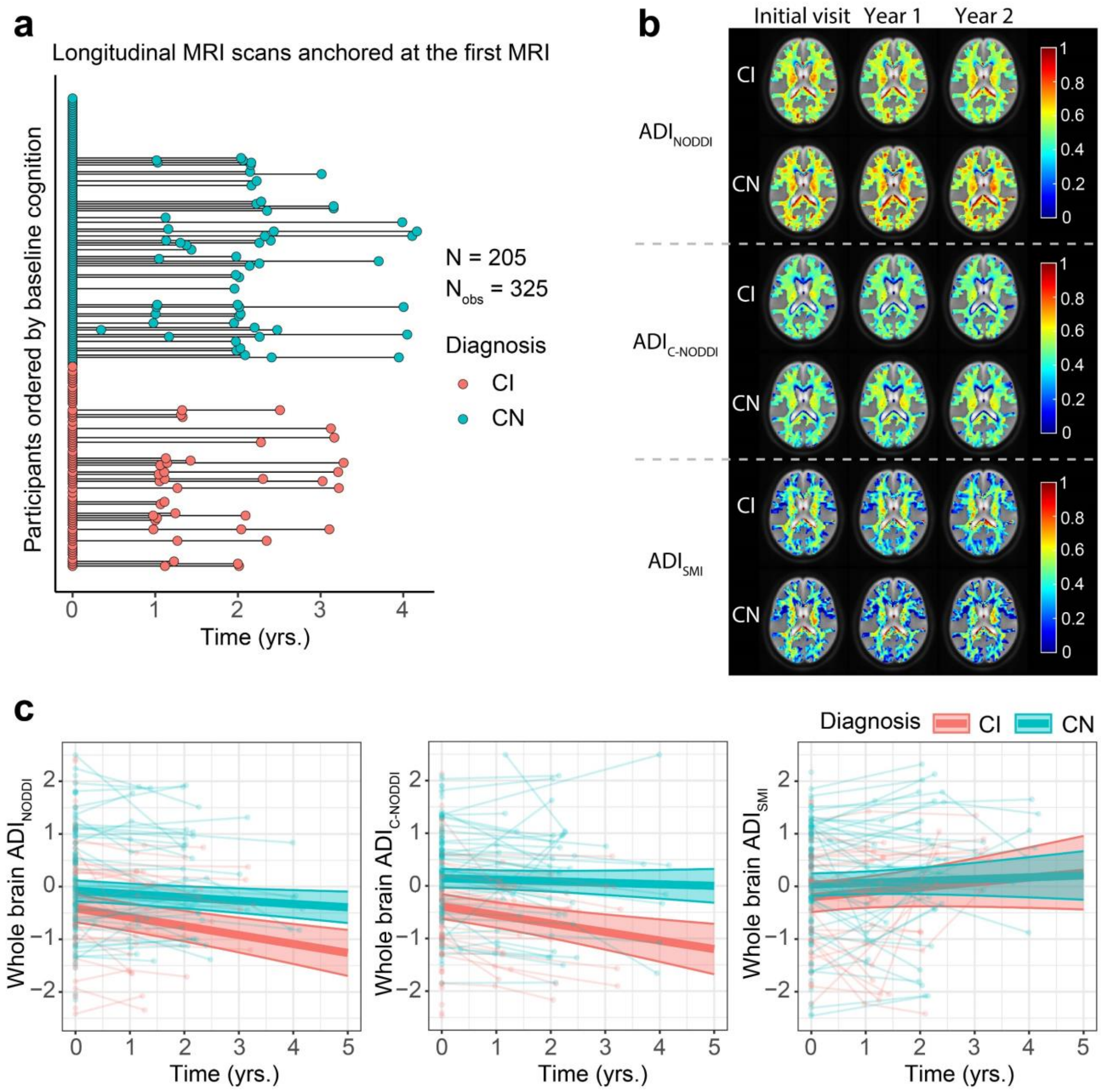

Figure S11. Characterization of longitudinal trajectories of axonal integrity in CN and MCI subjects. Overall, the results are consistent with those in Figure 2, showing slightly reduced effect sizes and statistical significance. $ADI_{C\text{-}NODDI}$ remains the best-performing biomarker for distinguishing differential trajectories between CN and MCI groups.

Table S8. Linear mixed-effects model results for Figure 3 without AD subjects.

The model equation is specified as:

$$\mathrm{MMSE/CDR\text{-}SB}_{ij} \sim \beta_0 + \beta_{Age} \times \mathrm{Age}_i + \beta_{sex} \times \mathrm{Sex}_i + \beta_{Time} \times \mathrm{Time}_{ij} + \beta_{Diagnosis} \times \mathrm{Diagnosis}_i + \beta_{ADI} \times \mathrm{ADI}_i + \beta_{Time\times ADI} \times \mathrm{Time}_{ij} \times \mathrm{ADI}_i + \beta_{Time\times Diagnosis} \times \mathrm{Time}_{ij} \times \mathrm{Diagnosis}_i + \beta_{ADI\times Diagnosis} \times \mathrm{ADI}_i \times \mathrm{Diagnosis}_i + \beta_{Time\times Diagnosis\times ADI} \times \mathrm{Time}_{ij} \times \mathrm{Diagnosis}_i \times \mathrm{ADI}_i + b_i + \epsilon_{ij},$$

where $\mathrm{MMSE/CDR\text{-}SB}_{ij}$ represents the longitudinal MMSE or CDR-SB scores for subject i at time point j; Age_i is the age at the baseline MRI measurement for subject i; Sex_i denotes the subject sex; Time_{ij} is the time of cognitive assessment since the baseline MRI measurement for subject i at time point j; $\mathrm{Diagnosis}_i$ indicates the cognitive diagnosis either as cognitive normal (CN) or cognitively impaired (CI, which only includes MCI) for subject i; and ADI_i is the baseline axonal density index. The model includes all two-way interactions among Time_{ij}, $\mathrm{Diagnosis}_i$, and ADI_i, as well as the three-way interaction term. b_i is the random intercept for subject i and ϵ_{ij} is the residual error. Age_i and ADI_i were standardized using baseline mean and standard deviation, while Time_{ij} was kept in the original unit (yrs.) for easier interpretation. Whole brain white matter $\mathrm{ADI}_{\mathrm{NODDI}}$, $\mathrm{ADI}_{\mathrm{C\text{-}NODDI}}$, and $\mathrm{ADI}_{\mathrm{SMI}}$ values were used. Full statistical results are presented below.

	$\mathrm{ADI}_{\mathrm{NODDI}}$ predicts prospective MMSE				
	Estimate	Std. Error	df	t value	Pr(>|t|)
(Intercept)	2.75e+01	2.66e-01	2.33e+02	1.03e+02	2.40e-196
Age	3.15e-02	1.22e-01	2.14e+02	2.59e-01	7.96e-01
Sex-Male	-2.70e-01	2.64e-01	1.92e+02	-1.02e+00	3.08e-01
Diagnosis-CN	1.66e+00	2.85e-01	2.54e+02	5.85e+00	1.54e-08
Time	-4.53e-01	9.84e-02	2.65e+02	-4.60e+00	6.43e-06
Whole brain ADI	-4.02e-01	2.06e-01	2.52e+02	-1.95e+00	5.23e-02
Diagnosis-CN × Time	3.49e-01	1.19e-01	2.61e+02	2.94e+00	3.60e-03
Diagnosis-CN × ADI	5.63e-01	2.78e-01	2.54e+02	2.03e+00	4.39e-02
Time × ADI	-1.52e-01	9.85e-02	2.75e+02	-1.54e+00	1.24e-01
Diagnosis-CN × Time × ADI	1.22e-01	1.21e-01	2.67e+02	1.01e+00	3.15e-01

	$ADI_{C\text{-}NODDI}$ predicts prospective MMSE				
	Estimate	Std. Error	df	t value	Pr(>|t|)
(Intercept)	2.76e+01	2.71e-01	2.35e+02	1.02e+02	1.27e-196
Age	1.33e-01	1.36e-01	2.03e+02	9.80e-01	3.28e-01
Sex-Male	-2.66e-01	2.58e-01	1.88e+02	-1.03e+00	3.04e-01
Diagnosis-CN	1.54e+00	2.97e-01	2.55e+02	5.19e+00	4.27e-07
Time	-3.40e-01	1.08e-01	2.71e+02	-3.14e+00	1.86e-03
Whole brain ADI	2.08e-01	2.33e-01	2.48e+02	8.93e-01	3.73e-01
Diagnosis-CN × Time	2.44e-01	1.30e-01	2.65e+02	1.88e+00	6.10e-02
Diagnosis-CN × ADI	2.99e-02	2.96e-01	2.52e+02	1.01e-01	9.20e-01
Time × ADI	1.28e-01	1.11e-01	2.82e+02	1.16e+00	2.48e-01
Diagnosis-CN × Time × ADI	-1.75e-01	1.32e-01	2.75e+02	-1.33e+00	1.85e-01

	ADI_{SMI} predicts prospective MMSE				
	Estimate	Std. Error	df	t value	Pr(>|t|)
(Intercept)	2.76e+01	2.64e-01	2.32e+02	1.04e+02	8.98e-197
Age	4.43e-02	1.18e-01	2.10e+02	3.76e-01	7.07e-01
Sex-Male	-2.39e-01	2.55e-01	1.89e+02	-9.37e-01	3.50e-01
Diagnosis-CN	1.64e+00	2.85e-01	2.53e+02	5.77e+00	2.30e-08
Time	-3.21e-01	1.03e-01	2.66e+02	-3.12e+00	2.04e-03
Whole brain ADI	1.34e-01	2.43e-01	2.64e+02	5.53e-01	5.81e-01
Diagnosis-CN × Time	2.26e-01	1.23e-01	2.61e+02	1.83e+00	6.79e-02
Diagnosis-CN × ADI	-6.80e-02	2.96e-01	2.63e+02	-2.30e-01	8.18e-01
Time × ADI	1.70e-01	1.03e-01	2.58e+02	1.65e+00	9.97e-02
Diagnosis-CN × Time × ADI	-2.50e-01	1.23e-01	2.55e+02	-2.03e+00	4.29e-02

	ADI$_{NODDI}$ predicts prospective CDR-SB				
	Estimate	Std. Error	df	t value	Pr(>|t|)
(Intercept)	1.56e+00	1.42e-01	2.29e+02	1.09e+01	1.06e-22
Age	-5.63e-02	6.58e-02	2.01e+02	-8.56e-01	3.93e-01
Sex-Male	-8.60e-03	1.42e-01	1.94e+02	-6.05e-02	9.52e-01
Diagnosis-CN	-1.55e+00	1.51e-01	2.46e+02	-1.03e+01	7.75e-21
Time	3.24e-01	4.44e-02	2.87e+02	7.31e+00	2.69e-12
Whole brain ADI	9.29e-04	1.11e-01	2.43e+02	8.38e-03	9.93e-01
Diagnosis-CN × Time	-2.80e-01	5.33e-02	2.85e+02	-5.26e+00	2.87e-07
Diagnosis-CN × ADI	9.28e-03	1.46e-01	2.47e+02	6.34e-02	9.49e-01
Time × ADI	-5.88e-03	4.23e-02	2.95e+02	-1.39e-01	8.90e-01
Diagnosis-CN × Time × ADI	-6.54e-03	5.20e-02	2.89e+02	-1.26e-01	9.00e-01

	ADI$_{C\text{-}NODDI}$ predicts prospective CDR-SB				
	Estimate	Std. Error	df	t value	Pr(>|t|)
(Intercept)	1.49e+00	1.41e-01	2.37e+02	1.05e+01	1.56e-21
Age	-1.17e-01	7.19e-02	1.99e+02	-1.63e+00	1.04e-01
Sex-Male	-3.84e-02	1.34e-01	1.95e+02	-2.87e-01	7.74e-01
Diagnosis-CN	-1.46e+00	1.53e-01	2.51e+02	-9.57e+00	1.07e-18
Time	2.52e-01	4.90e-02	2.91e+02	5.14e+00	5.04e-07
Whole brain ADI	-2.98e-01	1.22e-01	2.46e+02	-2.43e+00	1.58e-02
Diagnosis-CN × Time	-2.09e-01	5.77e-02	2.89e+02	-3.62e+00	3.47e-04
Diagnosis-CN × ADI	2.37e-01	1.51e-01	2.51e+02	1.57e+00	1.19e-01
Time × ADI	-1.35e-01	5.06e-02	3.00e+02	-2.67e+00	8.02e-03
Diagnosis-CN × Time × ADI	1.39e-01	5.92e-02	2.96e+02	2.36e+00	1.91e-02

	ADI_{SMI} predicts prospective CDR-SB				
	Estimate	Std. Error	df	t value	Pr(>|t|)
(Intercept)	1.53e+00	1.36e-01	2.31e+02	1.12e+01	1.38e-23
Age	-6.46e-02	6.13e-02	2.00e+02	-1.05e+00	2.93e-01
Sex-Male	-1.52e-02	1.32e-01	1.92e+02	-1.16e-01	9.08e-01
Diagnosis-CN	-1.52e+00	1.46e-01	2.50e+02	-1.04e+01	3.13e-21
Time	2.74e-01	4.46e-02	2.92e+02	6.13e+00	2.79e-09
Whole brain ADI	-2.19e-01	1.19e-01	2.51e+02	-1.83e+00	6.79e-02
Diagnosis-CN × Time	-2.35e-01	5.35e-02	2.89e+02	-4.40e+00	1.50e-05
Diagnosis-CN × ADI	1.84e-01	1.46e-01	2.54e+02	1.26e+00	2.08e-01
Time × ADI	-1.28e-01	4.76e-02	2.86e+02	-2.70e+00	7.38e-03
Diagnosis-CN × Time × ADI	1.59e-01	5.57e-02	2.87e+02	2.86e+00	4.58e-03

Diagnosis was referenced to the MCI group, and sex was referenced to females. P-values were not corrected for multiple comparisons.

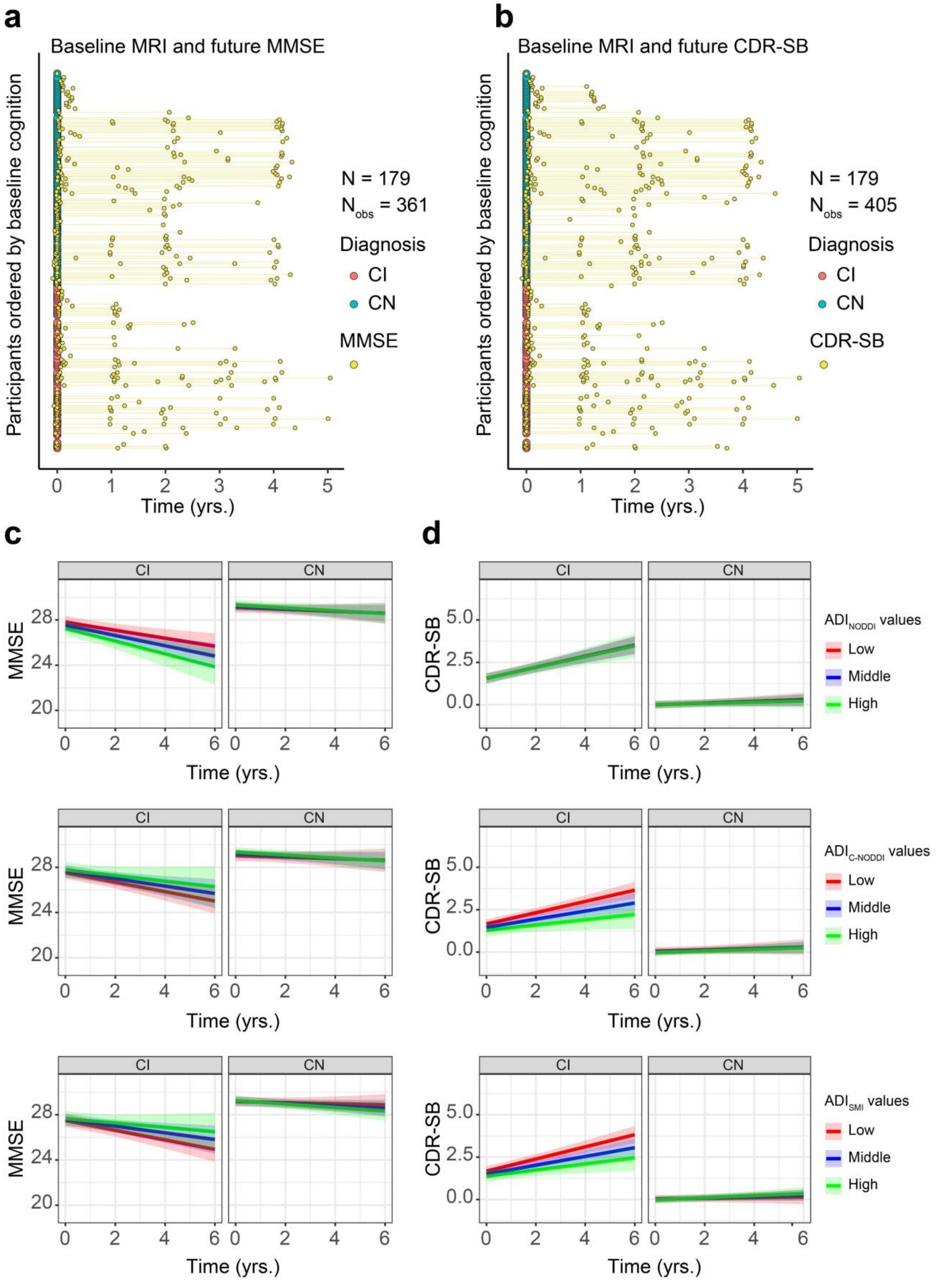
a
Baseline MRI and future MMSE
Participants ordered by baseline cognition
Time (yrs.)
N = 179
N_obs = 361
Diagnosis
CI
CN
MMSE
b
Baseline MRI and future CDR-SB
Participants ordered by baseline cognition
Time (yrs.)
N = 179
N_obs = 405
Diagnosis
CI
CN
CDR-SB
c
d
CI
CN
MMSE
CDR-SB
Time (yrs.)
ADI_NODDI values
ADI_C-NODDI values
ADI_SMI values
Low
Middle
High

Figure S12. Baseline MRI measurements of axonal density/integrity, assessed using the Axonal Density Index (ADI), predict future changes in cognition and function as measured by MMSE and CDR-SB scores. None of the three ADI metrics significantly predicted MMSE decline within the CN or MCI groups. However, both $ADI_{C\text{-}NODDI}$ and ADI_{SMI} predicted that higher baseline ADI was associated with lower function in the MCI group. These results are not conclusive and may reflect limited sensitivity to the smaller differences between CN and MCI due to the modest sample size. Future studies with larger, multi-cohort datasets are underway.

Table S9. Linear mixed-effects model results for Figure 4 without AD subjects.

The model equation is specified as:

$$\text{MMSE/CDR-SB changes}_{\text{ij}} \sim \beta_0 + \beta_{\text{sex}} \times \text{Sex}_{\text{i}} + \beta_{\text{Age}} \times \text{Age}_{\text{ij}} + \beta_{\text{Diagnosis}} \times \text{Diagnosis}_{\text{i}} + \beta_{\text{ADI changes}} \times \text{ADI changes}_{ij} + \beta_{\text{Diagnosis}\times\text{ADI changes}} \times \text{Diagnosis}_{\text{i}} \times \text{ADI changes}_{ij} + \beta_{\text{Baseline ADI}} \times \text{Baseline ADI}_{\text{i}} + \beta_{\text{Diagnosis}\times\text{Baseline ADI}} \times \text{Diagnosis}_{\text{i}} \times \text{Baseline ADI}_{\text{i}} + \text{b}_{\text{i}} + \epsilon_{\text{ij}},$$

where $\text{MMSE/CDR-SB}_{\text{ij}}$ is the longitudinal MMSE or CDR-SB changes for subject i at time point j from subject i's baseline; Age_{ij} is the age at the MRI measurement for subject i at the time point j; Sex_{i} denotes the subject sex; ADI changes_{ij} is the change in ADI from baseline ADI for subject i at time point j; $\text{Baseline ADI}_{\text{i}}$ is the baseline ADI value for subject i; $\text{Diagnosis}_{\text{i}}$ is the cognitive diagnosis either as cognitive normal (CN) or cognitively impaired (CI, which only includes MCI) for subject i; b_{i} is the random intercept for subject i, and ϵ_{ij} is the residual error. Two-way interaction terms between diagnosis and ADI changes were included to examine how changes in ADI from baseline are associated with changes in MMSE/CDR-SB scores. Age_{ij} and ADI_{ij} values were standardized using the baseline mean and standard deviation. ADI $\text{changes}_{\text{ij}}$ were computed as the difference between the standardized ADI at time j and the subject's baseline ADI. Whole brain white matter $\text{ADI}_{\text{NODDI}}$, $\text{ADI}_{\text{C-NODDI}}$, and ADI_{SMI} values were used. Full statistical results are presented below.

	$\text{ADI}_{\text{NODDI}}$ changes associate with MMSE				
	Estimate	Std. Error	df	t value	Pr(>|t|)
(Intercept)	2.74e+01	2.30e-01	1.91e+02	1.19e+02	2.62e-181
Sex-Male	-1.75e-01	2.38e-01	1.76e+02	-7.36e-01	4.63e-01
Age	2.54e-02	1.17e-01	1.99e+02	2.16e-01	8.29e-01
Diagnosis-CN	1.67e+00	2.40e-01	1.86e+02	6.95e+00	6.14e-11
ADI changes	7.59e-01	4.32e-01	1.70e+02	1.76e+00	8.07e-02
ADI baseline	-3.27e-01	1.75e-01	1.88e+02	-1.87e+00	6.30e-02
Diagnosis-CN × ADI changes	-8.32e-01	6.07e-01	1.65e+02	-1.37e+00	1.73e-01
Diagnosis-CN × ADI baseline	4.43e-01	2.32e-01	1.83e+02	1.91e+00	5.80e-02

	$ADI_{C\text{-}NODDI}$ changes associate with MMSE				
	Estimate	Std. Error	df	t value	Pr(>|t|)
(Intercept)	2.75e+01	2.38e-01	1.93e+02	1.16e+02	1.91e-180
Sex-Male	-1.65e-01	2.35e-01	1.74e+02	-7.01e-01	4.84e-01
Age	7.00e-02	1.32e-01	1.97e+02	5.29e-01	5.97e-01
Diagnosis-CN	1.61e+00	2.55e-01	1.89e+02	6.31e+00	1.89e-09
ADI changes	1.21e-01	4.99e-01	1.75e+02	2.42e-01	8.09e-01
ADI baseline	4.81e-02	2.00e-01	1.84e+02	2.40e-01	8.11e-01
Diagnosis-CN × ADI changes	-5.44e-01	5.82e-01	1.73e+02	-9.34e-01	3.52e-01
Diagnosis-CN × ADI baseline	7.90e-02	2.50e-01	1.79e+02	3.16e-01	7.53e-01

	ADI_{SMI} changes associate with MMSE				
	Estimate	Std. Error	df	t value	Pr(>|t|)
(Intercept)	2.75e+01	2.27e-01	1.82e+02	1.21e+02	2.34e-175
Sex-Male	-1.27e-01	2.31e-01	1.73e+02	-5.48e-01	5.84e-01
Age	2.33e-02	1.13e-01	1.95e+02	2.06e-01	8.37e-01
Diagnosis-CN	1.66e+00	2.38e-01	1.74e+02	6.96e+00	6.66e-11
ADI changes	1.40e-01	2.53e-01	1.44e+02	5.52e-01	5.82e-01
ADI baseline	1.85e-01	2.02e-01	1.68e+02	9.17e-01	3.61e-01
Diagnosis-CN × ADI changes	2.86e-01	3.46e-01	1.58e+02	8.27e-01	4.09e-01
Diagnosis-CN × ADI baseline	-1.83e-01	2.43e-01	1.69e+02	-7.53e-01	4.52e-01

	ADI_{NODDI} changes associate with CDR-SB				
	Estimate	Std. Error	df	t value	Pr(>|t|)
(Intercept)	1.71e+00	1.16e-01	1.91e+02	1.47e+01	2.75e-33
Sex-Male	-7.29e-02	1.20e-01	1.83e+02	-6.07e-01	5.45e-01
Age	-3.83e-02	5.83e-02	2.04e+02	-6.56e-01	5.12e-01
Diagnosis-CN	-1.65e+00	1.20e-01	1.88e+02	-1.37e+01	5.19e-30
ADI changes	-5.28e-01	1.79e-01	1.38e+02	-2.95e+00	3.75e-03
ADI baseline	1.28e-02	8.82e-02	1.88e+02	1.45e-01	8.85e-01
Diagnosis-CN × ADI changes	4.51e-01	2.56e-01	1.37e+02	1.76e+00	8.06e-02
Diagnosis-CN × ADI baseline	-5.48e-03	1.16e-01	1.87e+02	-4.73e-02	9.62e-01

	$ADI_{C\text{-}NODDI}$ changes associate with CDR-SB				
	Estimate	Std. Error	df	t value	Pr(>|t|)
(Intercept)	1.59e+00	1.17e-01	1.93e+02	1.37e+01	3.53e-30
Sex-Male	-9.09e-02	1.15e-01	1.84e+02	-7.91e-01	4.30e-01
Age	-1.04e-01	6.41e-02	2.07e+02	-1.62e+00	1.07e-01
Diagnosis-CN	-1.51e+00	1.25e-01	1.91e+02	-1.21e+01	1.55e-25
ADI changes	-8.61e-01	1.96e-01	1.40e+02	-4.38e+00	2.29e-05
ADI baseline	-2.64e-01	1.00e-01	1.88e+02	-2.64e+00	8.94e-03
Diagnosis-CN × ADI changes	8.10e-01	2.33e-01	1.38e+02	3.48e+00	6.69e-04
Diagnosis-CN × ADI baseline	2.18e-01	1.22e-01	1.86e+02	1.78e+00	7.67e-02

	ADI_{SMI} changes associate with CDR-SB				
	Estimate	Std. Error	df	t value	Pr(>|t|)
(Intercept)	1.69e+00	1.13e-01	1.86e+02	1.50e+01	5.75e-34
Sex-Male	-6.01e-02	1.14e-01	1.81e+02	-5.25e-01	6.00e-01
Age	-3.72e-02	5.53e-02	2.04e+02	-6.73e-01	5.01e-01
Diagnosis-CN	-1.63e+00	1.18e-01	1.83e+02	-1.38e+01	2.89e-30
ADI changes	1.60e-01	1.09e-01	1.27e+02	1.46e+00	1.46e-01
ADI baseline	-2.22e-01	9.73e-02	1.81e+02	-2.28e+00	2.38e-02
Diagnosis-CN × ADI changes	-1.65e-01	1.52e-01	1.36e+02	-1.09e+00	2.79e-01
Diagnosis-CN × ADI baseline	2.27e-01	1.19e-01	1.83e+02	1.90e+00	5.84e-02

Diagnosis is referenced to MCI and Sex is referenced to Female. All p-values are not corrected for multiple comparisons.

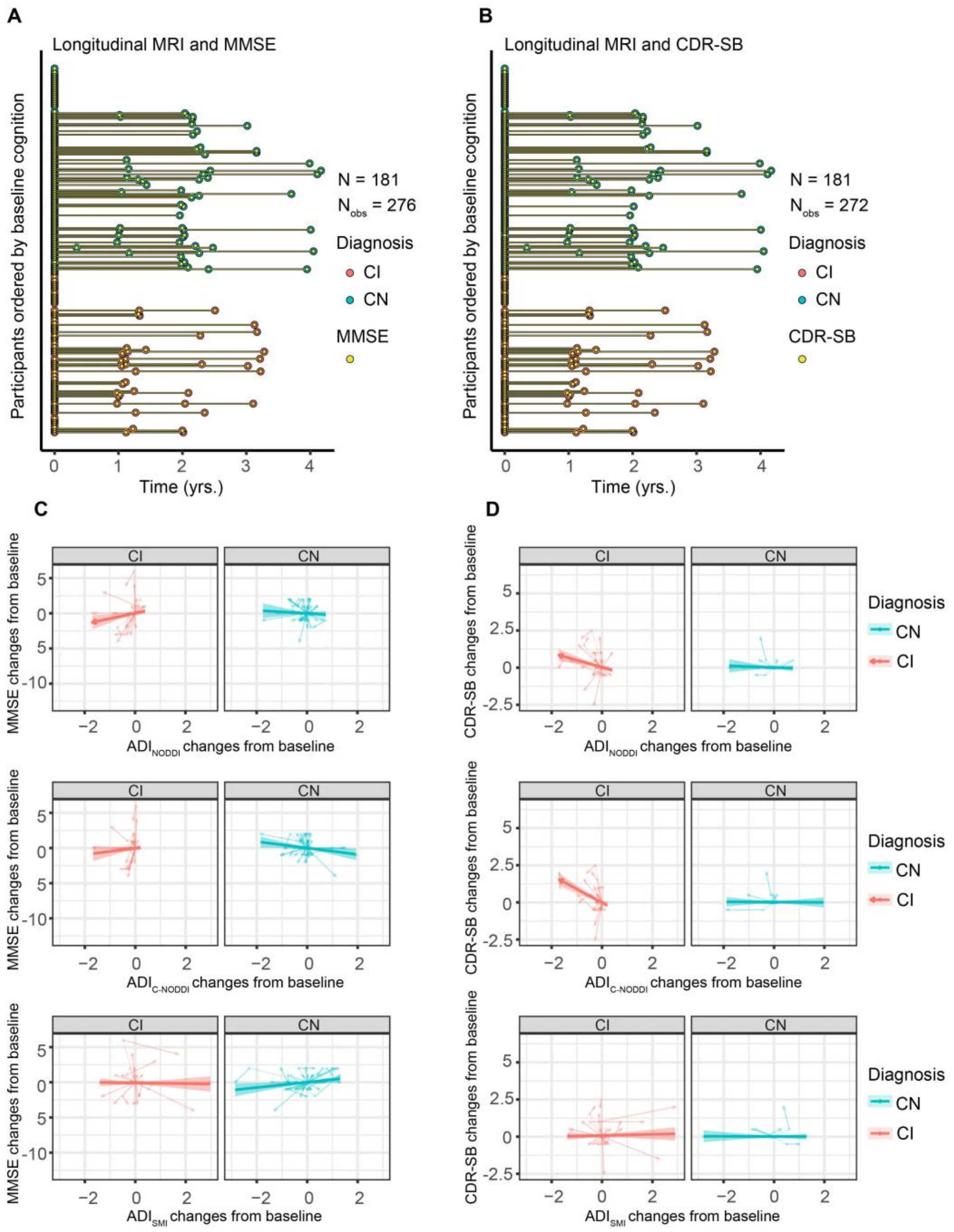

Figure S13. Changes in axonal integrity, measured using the Axonal Density Index (ADI), are associated with changes in cognition and function, assessed by MMSE and CDR-SB scores. Only changes in ADI_{NODDI} significantly associated with MMSE decline within MCI groups. However, decreases in ADI_{NODDI} and $ADI_{C\text{-}NODDI}$ were significantly associated with CDR-SB increases in the MCI group. These results are not conclusive and may reflect limited sensitivity to subtle differences between CN and MCI due to the modest sample size. Future studies involving larger, multi-cohort datasets are underway.

Supplemental Acknowledgments

Michael Weiner, MD (UC San Francisco, Principal Investigator, Executive Committee); Paul Aisen, MD (UC San Diego, ADCS PI and Director of Coordinating Center Clinical Core, Executive Committee, Clinical Core Leaders); Ronald Petersen, MD, PhD (Mayo Clinic, Rochester, Executive Committee, Clinical Core Leader); Clifford R. Jack, Jr., MD (Mayo Clinic, Rochester, Executive Committee, MRI Core Leader); William Jagust, MD (UC Berkeley, Executive Committee; PET Core Leader); John Q. Trojanowki, MD, PhD (U Pennsylvania, Executive Committee, Biomarkers Core Leader); Arthur W. Toga, PhD (USC, Executive Committee, Informatics Core Leader); Laurel Beckett, PhD (UC Davis, Executive Committee, Biostatistics Core Leader); Robert C. Green, MD, MPH (Brigham and Women's Hospital, Harvard Medical School, Executive Committee and Chair of Data and Publication Committee); Andrew J. Saykin, PsyD (Indiana University, Executive Committee, Genetics Core Leader); John Morris, MD (Washington University St. Louis, Executive Committee, Neuropathology Core Leader); Leslie M. Shaw (University of Pennsylvania, Executive Committee, Biomarkers Core Leader); Enchi Liu, PhD (Janssen Alzheimer Immunotherapy, ADNI 2 Private Partner Scientific Board Chair); Tom Montine, MD, PhD (University of Washington) ; Ronald G. Thomas, PhD (UC San Diego); Michael Donohue, PhD (UC San Diego); Sarah Walter, MSc (UC San Diego); Devon Gessert (UC San Diego); Tamie Sather, MS (UC San Diego,); Gus Jiminez, MBS (UC San Diego); Danielle Harvey, PhD (UC Davis;); Michael Donohue, PhD (UC San Diego); Matthew Bernstein, PhD (Mayo Clinic, Rochester); Nick Fox, MD (University of London); Paul Thompson, PhD (USC School of Medicine); Norbert Schuff, PhD (UCSF MRI); Charles DeCArli, MD (UC Davis); Bret Borowski, RT (Mayo Clinic); Jeff Gunter, PhD (Mayo Clinic); Matt Senjem, MS (Mayo Clinic); Prashanthi Vemuri, PhD (Mayo Clinic); David Jones, MD (Mayo Clinic); Kejal Kantarci (Mayo Clinic); Chad Ward (Mayo Clinic); Robert A. Koeppe, PhD (University of Michigan, PET Core Leader); Norm Foster, MD (University of Utah); Eric M. Reiman, MD (Banner Alzheimer's Institute); Kewei Chen, PhD (Banner Alzheimer's Institute); Chet Mathis, MD (University of Pittsburgh); Susan Landau, PhD (UC Berkeley); Nigel J. Cairns, PhD, MRCPath (Washington University St. Louis); Erin Householder (Washington University St. Louis); Lisa Taylor Reinwald, BA, HTL (Washington University St. Louis); Virginia Lee, PhD, MBA (UPenn School of Medicine); Magdalena Korecka, PhD (UPenn School of Medicine); Michal Figurski, PhD (UPenn School of Medicine); Karen Crawford (USC); Scott Neu, PhD (USC); Tatiana M. Foroud, PhD (Indiana University); Steven Potkin, MD UC (UC Irvine); Li Shen, PhD (Indiana University); Faber Kelley, MS, CCRC (Indiana University); Sungeun Kim, PhD (Indiana University); Kwangsik Nho, PhD (Indiana University); Zaven Kachaturian, PhD (Khachaturian, Radebaugh & Associates, Inc and Alzheimer's Association's Ronald and Nancy Reagan's Research Institute); Richard Frank, MD, PhD (General Electric); Peter J. Snyder, PhD (Brown University); Susan Molchan, PhD (National Institute on Aging/ National Institutes of Health); Jeffrey Kaye, MD (Oregon Health and Science University); Joseph Quinn, MD (Oregon Health and Science University); Betty Lind, BS (Oregon Health and Science University); Raina Carter, BA (Oregon Health and Science University); Sara Dolen, BS (Oregon Health and Science University); Lon S. Schneider, MD (University of Southern California); Sonia Pawluczyk, MD (University of Southern California); Mauricio Beccera, BS (University of Southern California); Liberty Teodoro, RN (University of Southern California); Bryan M. Spann, DO, PhD (University of Southern California); James Brewer, MD, PhD (University of California San Diego); Helen Vanderswag, RN (University of California San Diego); Adam Fleisher, MD (University of California San Diego); Judith L. Heidebrink, MD, MS (University of Michigan); Joanne L. Lord, LPN, BA, CCRC (University of Michigan); Ronald Petersen, MD, PhD (Mayo Clinic, Rochester); Sara S. Mason, RN (Mayo Clinic, Rochester); Colleen S. Albers, RN (Mayo Clinic, Rochester); David Knopman, MD (Mayo

Clinic, Rochester); Kris Johnson, RN (Mayo Clinic, Rochester); Rachelle S. Doody, MD, PhD (Baylor College of Medicine); Javier Villanueva Meyer, MD (Baylor College of Medicine); Munir Chowdhury, MBBS, MS (Baylor College of Medicine); Susan Rountree, MD (Baylor College of Medicine); Mimi Dang, MD (Baylor College of Medicine); Yaakov Stern, PhD (Columbia University Medical Center); Lawrence S. Honig, MD, PhD (Columbia University Medical Center); Karen L. Bell, MD (Columbia University Medical Center); Beau Ances, MD (Washington University, St. Louis); John C. Morris, MD (Washington University, St. Louis); Maria Carroll, RN, MSN (Washington University, St. Louis); Sue Leon, RN, MSN (Washington University, St. Louis); Erin Householder, MS, CCRP (Washington University, St. Louis); Mark A. Mintun, MD (Washington University, St. Louis); Stacy Schneider, APRN, BC, GNP (Washington University, St. Louis); Angela Oliver, RN, BSN, MSG ; Daniel Marson, JD, PhD (University of Alabama Birmingham); Randall Griffith, PhD, ABPP (University of Alabama Birmingham); David Clark, MD (University of Alabama Birmingham); David Geldmacher, MD (University of Alabama Birmingham); John Brockington, MD (University of Alabama Birmingham); Erik Roberson, MD (University of Alabama Birmingham); Hillel Grossman, MD (Mount Sinai School of Medicine); Effie Mitsis, PhD (Mount Sinai School of Medicine); Leyla deToledo-Morrell, PhD (Rush University Medical Center); Raj C. Shah, MD (Rush University Medical Center); Ranjan Duara, MD (Wien Center); Daniel Varon, MD (Wien Center); Maria T. Greig, HP (Wien Center); Peggy Roberts, CNA (Wien Center); Marilyn Albert, PhD (Johns Hopkins University); Chiadi Onyike, MD (Johns Hopkins University); Daniel D'Agostino II, BS (Johns Hopkins University); Stephanie Kielb, BS (Johns Hopkins University); James E. Galvin, MD, MPH (New York University); Dana M. Pogorelec (New York University); Brittany Cerbone (New York University); Christina A. Michel (New York University); Henry Rusinek, PhD (New York University); Mony J de Leon, EdD (New York University); Lidia Glodzik, MD, PhD (New York University); Susan De Santi, PhD (New York University); P. Murali Doraiswamy, MD (Duke University Medical Center); Jeffrey R. Petrella, MD (Duke University Medical Center); Terence Z. Wong, MD (Duke University Medical Center); Steven E. Arnold, MD (University of Pennsylvania); Jason H. Karlawish, MD (University of Pennsylvania); David Wolk, MD (University of Pennsylvania); Charles D. Smith, MD (University of Kentucky); Greg Jicha, MD (University of Kentucky); Peter Hardy, PhD (University of Kentucky); Partha Sinha, PhD (University of Kentucky); Elizabeth Oates, MD (University of Kentucky); Gary Conrad, MD (University of Kentucky); Oscar L. Lopez, MD (University of Pittsburgh); MaryAnn Oakley, MA (University of Pittsburgh); Donna M. Simpson, CRNP, MPH (University of Pittsburgh); Anton P. Porsteinsson, MD (University of Rochester Medical Center); Bonnie S. Goldstein, MS, NP (University of Rochester Medical Center); Kim Martin, RN (University of Rochester Medical Center); Kelly M. Makino, BS (University of Rochester Medical Center); M. Saleem Ismail, MD (University of Rochester Medical Center); Connie Brand, RN (University of Rochester Medical Center); Ruth A. Mulnard, DNSc, RN, FAAN (University of California, Irvine); Gaby Thai, MD (University of California, Irvine); Catherine Mc Adams Ortiz, MSN, RN, A/GNP (University of California, Irvine); Kyle Womack, MD (University of Texas Southwestern Medical School); Dana Mathews, MD, PhD (University of Texas Southwestern Medical School); Mary Quiceno, MD (University of Texas Southwestern Medical School); Ramon Diaz Arrastia, MD, PhD (University of Texas Southwestern Medical School); Richard King, MD (University of Texas Southwestern Medical School); Myron Weiner, MD (University of Texas Southwestern Medical School); Kristen Martin Cook, MA (University of Texas Southwestern Medical School); Michael DeVous, PhD (University of Texas Southwestern Medical School); Allan I. Levey, MD, PhD (Emory University); James J. Lah, MD, PhD (Emory University); Janet S. Cellar, DNP, PMHCNS BC (Emory University); Jeffrey M. Burns, MD (University of Kansas, Medical Center); Heather S. Anderson, MD (University of Kansas, Medical Center); Russell H. Swerdlow, MD (University of Kansas, Medical Center); Liana Apostolova, MD

(University of California, Los Angeles); Kathleen Tingus, PhD (University of California, Los Angeles); Ellen Woo, PhD (University of California, Los Angeles); Daniel H.S. Silverman, MD, PhD (University of California, Los Angeles); Po H. Lu, PsyD (University of California, Los Angeles); George Bartzokis, MD (University of California, Los Angeles); Neill R Graff Radford, MBBCH, FRCP (London) (Mayo Clinic, Jacksonville); Francine Parfitt, MSH, CCRC (Mayo Clinic, Jacksonville); Tracy Kendall, BA, CCRP (Mayo Clinic, Jacksonville); Heather Johnson, MLS, CCRP (Mayo Clinic, Jacksonville); Martin R. Farlow, MD (Indiana University); Ann Marie Hake, MD (Indiana University); Brandy R. Matthews, MD (Indiana University); Scott Herring, RN, CCRC (Indiana University); Cynthia Hunt, BS, CCRP (Indiana University); Christopher H. van Dyck, MD (Yale University School of Medicine); Richard E. Carson, PhD (Yale University School of Medicine); Martha G. MacAvoy, PhD (Yale University School of Medicine); Howard Chertkow, MD (McGill Univ., Montreal Jewish General Hospital); Howard Bergman, MD (McGill Univ., Montreal Jewish General Hospital); Chris Hosein, Med (McGill Univ., Montreal Jewish General Hospital); Sandra Black, MD, FRCPC (Sunnybrook Health Sciences, Ontario); Dr Bojana Stefanovic (Sunnybrook Health Sciences, Ontario); Curtis Caldwell, PhD (Sunnybrook Health Sciences, Ontario); Ging Yuek Robin Hsiung, MD, MHSc, FRCPC (U.B.C. Clinic for AD & Related Disorders); Howard Feldman, MD, FRCPC (U.B.C. Clinic for AD & Related Disorders); Benita Mudge, BS (U.B.C. Clinic for AD & Related Disorders); Michele Assaly, MA Past (U.B.C. Clinic for AD & Related Disorders); Andrew Kertesz, MD (Cognitive Neurology St. Joseph's, Ontario); John Rogers, MD (Cognitive Neurology St. Joseph's, Ontario); Dick Trost, PhD (Cognitive Neurology St. Joseph's, Ontario); Charles Bernick, MD (Cleveland Clinic Lou Ruvo Center for Brain Health); Donna Munic, PhD (Cleveland Clinic Lou Ruvo Center for Brain Health); Diana Kerwin, MD (Northwestern University); Marek Marsel Mesulam, MD (Northwestern University); Kristine Lipowski, BA (Northwestern University); Chuang Kuo Wu, MD, PhD (Northwestern University); Nancy Johnson, PhD (Northwestern University); Carl Sadowsky, MD (Premiere Research Inst (Palm Beach Neurology)); Walter Martinez, MD (Premiere Research Inst (Palm Beach Neurology)); Teresa Villena, MD (Premiere Research Inst (Palm Beach Neurology)); Raymond Scott Turner, MD, PhD (Georgetown University Medical Center); Kathleen Johnson, NP (Georgetown University Medical Center); Brigid Reynolds, NP (Georgetown University Medical Center); Reisa A. Sperling, MD (Brigham and Women's Hospital); Keith A. Johnson, MD (Brigham and Women's Hospital); Gad Marshall, MD (Brigham and Women's Hospital); Meghan Frey (Brigham and Women's Hospital); Jerome Yesavage, MD (Stanford University); Joy L. Taylor, PhD (Stanford University); Barton Lane, MD (Stanford University); Allyson Rosen, PhD (Stanford University); Jared Tinklenberg, MD (Stanford University); Marwan N. Sabbagh, MD (Banner Sun Health Research Institute); Christine M. Belden, PsyD (Banner Sun Health Research Institute); Sandra A. Jacobson, MD (Banner Sun Health Research Institute); Sherye A. Sirrel, MS (Banner Sun Health Research Institute); Neil Kowall, MD (Boston University); Ronald Killiany, PhD (Boston University); Andrew E. Budson, MD (Boston University); Alexander Norbash, MD (Boston University); Patricia Lynn Johnson, BA (Boston University); Thomas O. Obisesan, MD, MPH (Howard University); Saba Wolday, MSc (Howard University); Joanne Allard, PhD (Howard University); Alan Lerner, MD (Case Western Reserve University); Paula Ogrocki, PhD (Case Western Reserve University); Leon Hudson, MPH (Case Western Reserve University); Evan Fletcher, PhD (University of California, Davis Sacramento); Owen Carmichael, PhD (University of California, Davis Sacramento); John Olichney, MD (University of California, Davis Sacramento); Charles DeCarli, MD (University of California, Davis Sacramento); Smita Kittur, MD (Neurological Care of CNY); Michael Borrie, MB ChB (Parkwood Hospital); T Y Lee, PhD (Parkwood Hospital); Dr Rob Bartha, PhD (Parkwood Hospital); Sterling Johnson, PhD (University of Wisconsin); Sanjay Asthana, MD (University of Wisconsin); Cynthia M. Carlsson, MD (University of Wisconsin); Steven G. Potkin, MD (University of California, Irvine

BIC); Adrian Preda, MD (University of California, Irvine BIC); Dana Nguyen, PhD (University of California, Irvine BIC); Pierre Tariot, MD (Banner Alzheimer's Institute); Adam Fleisher, MD (Banner Alzheimer's Institute); Stephanie Reeder, BA (Banner Alzheimer's Institute); Vernice Bates, MD (Dent Neurologic Institute); Horacio Capote, MD (Dent Neurologic Institute); Michelle Rainka, PharmD, CCRP (Dent Neurologic Institute); Douglas W. Scharre, MD (Ohio State University); Maria Kataki, MD, PhD (Ohio State University); Anahita Adeli, MD (Ohio State University); Earl A. Zimmerman, MD (Albany Medical College); Dzintra Celmins, MD (Albany Medical College); Alice D. Brown, FNP (Albany Medical College); Godfrey D. Pearlson, MD (Hartford Hosp, Olin Neuropsychiatry Research Center); Karen Blank, MD (Hartford Hosp, Olin Neuropsychiatry Research Center); Karen Anderson, RN (Hartford Hosp, Olin Neuropsychiatry Research Center); Robert B. Santulli, MD (Dartmouth Hitchcock Medical Center); Tamar J. Kitzmiller (Dartmouth Hitchcock Medical Center); Eben S. Schwartz, PhD (Dartmouth Hitchcock Medical Center); Kaycee M. Sink, MD, MAS (Wake Forest University Health Sciences); Jeff D. Williamson, MD, MHS (Wake Forest University Health Sciences); Pradeep Garg, PhD (Wake Forest University Health Sciences); Franklin Watkins, MD (Wake Forest University Health Sciences); Brian R. Ott, MD (Rhode Island Hospital); Henry Querfurth, MD (Rhode Island Hospital); Geoffrey Tremont, PhD (Rhode Island Hospital); Stephen Salloway, MD, MS (Butler Hospital); Paul Malloy, PhD (Butler Hospital); Stephen Correia, PhD (Butler Hospital); Howard J. Rosen, MD (UC San Francisco); Bruce L. Miller, MD (UC San Francisco); Jacobo Mintzer, MD, MBA (Medical University South Carolina); Kenneth Spicer, MD, PhD (Medical University South Carolina); David Bachman, MD (Medical University South Carolina); Elizabether Finger, MD (St. Joseph's Health Care); Stephen Pasternak, MD (St. Joseph's Health Care); Irina Rachinsky, MD (St. Joseph's Health Care); John Rogers, MD (St. Joseph's Health Care); Andrew Kertesz, MD (St. Joseph's Health Care); Dick Drost, MD (St. Joseph's Health Care); Nunzio Pomara, MD (Nathan Kline Institute); Raymundo Hernando, MD (Nathan Kline Institute); Antero Sarrael, MD (Nathan Kline Institute); Susan K. Schultz, MD (University of Iowa College of Medicine, Iowa City); Laura L. Boles Ponto, PhD (University of Iowa College of Medicine, Iowa City); Hyungsub Shim, MD (University of Iowa College of Medicine, Iowa City); Karen Elizabeth Smith, RN (University of Iowa College of Medicine, Iowa City); Norman Relkin, MD, PhD (Cornell University); Gloria Chaing, MD (Cornell University); Lisa Raudin, PhD (Cornell University); Amanda Smith, MD (University of South Floriday: USF Health Byrd Alzheimer's Institute); Kristin Fargher, MD (University of South Floriday: USF Health Byrd Alzheimer's Institute); Balebail Ashok Raj, MD (University of South Floriday: USF Health Byrd Alzheimer's Institute)
